# Scalable high resolution ancestry deconvolution for genomic data

**DOI:** 10.1038/s41467-026-75391-0

**Published:** 2026-07-25

**Authors:** Helgi Hilmarsson, Arvind S. Kumar, Míriam Barrabés, Richa Rastogi, Carlos D. Bustamante, Daniel Mas Montserrat, Alexander G. Ioannidis

**Affiliations:** 1 Stanford University, Institute for Computational and Mathematical Engineering, Stanford, CA USA; 2 Stanford University, Department of Biomedical Data Science, Stanford, CA USA; 3 Cornell University, Department of Computer Science, New York, USA; 4 University of Oklahoma, College of Medicine, Norman, OK USA; 5 Genomics Institute, University of California Santa Cruz, Santa Cruz, CA USA

**Keywords:** Population genetics, Genome informatics

## Abstract

As genome-wide association studies and genetic risk prediction models extend to globally diverse and admixed biobanks, accurate, scalable ancestry deconvolution, also called local ancestry inference (LAI), has become crucial. LAI assigns ancestry to each genomic segment within an individual, enabling studies of population history and ancestry-associated haplotypic effects. Existing LAI methods scale poorly to biobank-scale data, to the distant past, and to large numbers of ancestries. Here, we introduce several independent LAI methods implemented in the Gnomix software suite, achieving higher accuracy and faster computational performance than all existing approaches and with portable models that can be shared without exposing individual-level training data. Gnomix is paired with Gnofix, a swift, scalable phase correction counterpart. We demonstrate performance on worldwide whole-genome data from humans and canids, leveraging high-resolution accuracy to localise ancient New World haplotypes in the Xoloitzcuintli, dating back over 100 generations. Code is available at https://github.com/AI-sandbox/gnomix.

## Abstract (web summary)

The authors present Gnomix, a local ancestry framework that delivers leading accuracy across diverse admixed datasets on both whole-genome and array data with high efficiency, together with Gnofix, its fast phasing-error correction counterpart.

## Introduction

Local ancestry inference (LAI), more descriptively referred to as locus-specific ancestry inference, assigns ancestry labels to genomic segments within admixed individuals, enabling ancestry resolution at fine genomic scales. By explicitly modelling the mosaic-like structure generated by recombination after admixture, LAI enables reconstruction of demographic history^1^ and identification of ancestry-specific genetic effects in genome-wide association studies^2^ and polygenic risk prediction models^3^. These capabilities are increasingly important as genomic datasets expand to include individuals from diverse and admixed populations. Despite growing recognition of the importance of population diversity in large-scale genetic studies^4^, most worldwide populations remain substantially underrepresented^5–7^, in part due to the complexities involved in analysing ancestrally diverse and admixed cohorts. Recent methodological advances have incorporated LAI to improve genetic analyses in admixed populations^8,9^. Such applications place increasing demands on LAI algorithms to operate efficiently, reproducibly, and accurately in large-scale cohorts.

Over the years, several methods for local ancestry inference have been published. SABER^10^, HAPAA^11^, and HAPMIX^12^ used Hidden Markov Models (HMMs). The accuracy and efficiency of these methods were later surpassed by LAMP^13^, a method that applied probability maximisation within a sliding window but could handle only a limited number of ancestries. Increased accuracy was enabled by machine learning classifiers from Support Vector Machines (SVM)^14^ to Random Forests (RF) with a conditional random field (RFMix)^15^; both are still used. Refinements of these themes, including ELAI (HMMs)^16^ and Loter (dynamic programming with template matching and bagging), have been developed since^17^. More recently, neural-network-based approaches have emerged, beginning with LAI-Net^18^, then SALAI-NET^19^, and most recently Orchestra^20^. SALAI-NET integrates reference-based feature extraction with convolutional modelling of local haplotype structure for improved ancestry inference, while the more recent Orchestra borrows SALAI-Net’s approach, but adds transformer-based smoothing. FLARE, another recent method, returns to a more traditional approach via an extended Li and Stephens model^21^. Most recently, Recomb-Mix continued in this vein with a simplified Li and Stephens model formulated as graph optimisation^22^. Apart from these two recent methods, prior methods have been severely challenged by the ballooning numbers of samples in modern biobanks and the large number of sites in increasingly affordable whole-genome sequences. As biobanks consisting of hundreds of thousands of individuals are now common^23–26^, and whole genome sequences of these biobanks are expanding, such limitations of LAI methods need to be addressed.

In this work, we present Gnomix, a flexible collection of several different new local ancestry algorithms, capable of assigning ancestry labels to DNA segments from massive biobanks of either whole-genome or single nucleotide polymorphism (SNP) array data with higher accuracy and swifter execution than competing methods. The framework has two stages: a set of classifiers (base models) that generate initial estimates of the ancestry probabilities within genomic windows, followed by a second stage consisting of another module (smoother) that learns to combine and refine these estimates, significantly increasing accuracy. The framework is highly modular and supports a variety of combinations of classifier and smoother algorithms, enabling the user to select the configuration with a suitable trade-off between compute time and accuracy. We present an in-depth analysis of these configurations and recommend combinations that achieve optimal trade-offs based on available computational resources and specific dataset characteristics. For the base models, we evaluate historically successful LAI classifiers, including random forests and support vector machines, alongside classical machine learning approaches such as logistic regression and gradient boosting. We also introduce a novel generalisation of the string kernel^27^, which we call the covariance reduction string kernel (CovRSK). This method employs a sampling technique to reduce redundant kernel features and acts as a regulariser, improving overall accuracy. For the smoother, we propose a data-driven approach that learns the correlation structure among populations and the distribution of recombination breakpoints directly from the data, without relying on simplified models or assumptions. We describe how sequentially training the base models and smoother on the same data induces a covariate shift in the second stage, and we introduce an alternative training pipeline to mitigate this subtle bias. We evaluate multiple base models – both novel and established – and find that in many cases Extreme Gradient Boosting (XGBoost, XGB) serves as the most effective smoother, outperforming alternatives such as linear convolutional filters (CNNs) and conditional random fields (CRFs). We demonstrate that multiple Gnomix configurations perform competitively with current state-of-the-art LAI methods in both accuracy and speed across diverse datasets. We also introduce Gnofix, a scalable phase correction algorithm that uses a trained Gnomix smoother to phase-segment ancestry probabilities. Such rephasing is essential for some forms of demographic inference, but no other scalable local ancestry method with rephasing exists. Finally, we showcase the combined capabilities of Gnomix and Gnofix in a real-world application by investigating the genomic history of New World dogs^28^.

## Results and discussion

### System overview

We present a supervised method that uses haplotypes from a reference panel of single-ancestry individuals from differentiated population groups to train a model that assigns ancestry estimates to each segment of admixed chromosomes with high accuracy. We encode phased biallelic SNPs using the standard 0/1 representation, where zero denotes the reference allele and one denotes the alternative allele. For diploid organisms, such as humans, maternal and paternal sequences are treated independently and assumed to be correctly locally phased, although long-range phasing errors can be corrected with our Gnofix algorithm, described below. If sequences are unphased, existing statistical phasing algorithms can be applied before running Gnomix. Since we cannot have SNP-level ground-truth ancestry labels for admixed individuals, we simulate admixed progeny from real single-ancestry genomes based on the human recombination map. More details on dataset generation are provided in the Methods section. Our system splits each haplotype into windows containing equal numbers of SNPs and produces one ancestry estimate per window. These initial probability estimates are then refined by the smoothing module to produce the final ancestry predictions.

### Base module

**Fig. 1:**
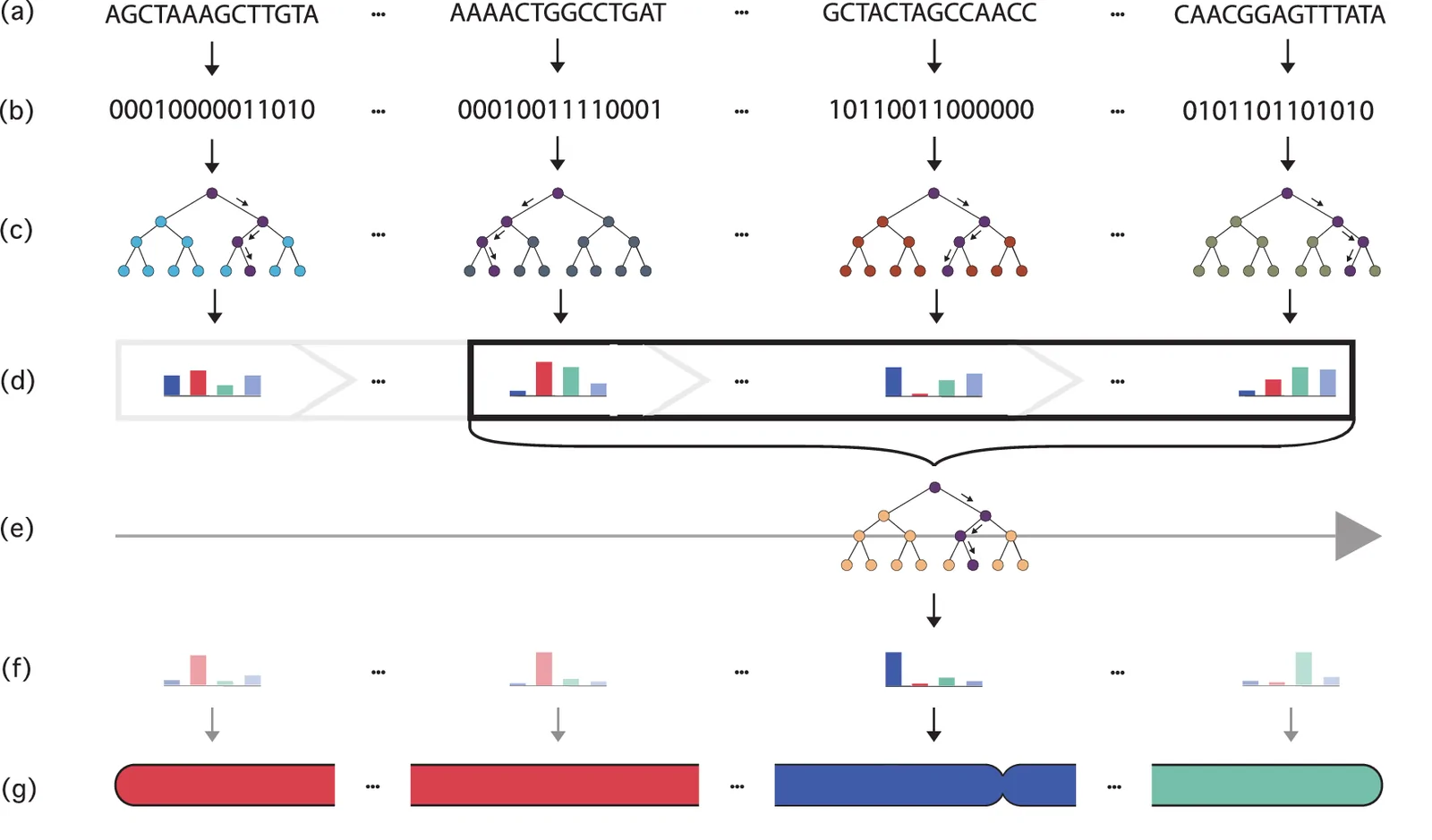
Schematic of the Gnomix algorithm. **a** Genome sequence segmentation. **b** Binary (biallelic) encoding. **c** Local base classifiers' learning procedure. **d** Probability estimates of local base classifiers. **e**, **f** Sliding smoother module trained to aggregate local base model probabilities. **g** Smoothing module outputs final local ancestry predictions along the chromosome.

In the first stage of the system, we produce initial ancestry estimates by assigning one model per window, each learning to map the SNPs within that window to ancestry probabilities (Fig. 1a–d). The 0/1 encoded biallelic SNP sequences are segmented into *W* equally sized windows, and each window is processed by a classifier. Because the learnt classification function is not translation invariant (an identical sequence of zeros and ones might correspond to different ancestries depending on its location in the genome), a different classifier is used for each windowed segment of the input sequence. All window-specific classifiers share the same hyperparameter configuration, but each learns its own set of model parameters. Since neighbouring SNPs are usually inherited together due to linkage, a window-based approach that jointly processes co-located SNPs is well-motivated. The independent treatment of windows enables highly parallelisable training and inference, resulting in significant time reductions that scale linearly with the number of available CPUs. Each model outputs a local ancestry probability estimate, which can be interpreted as an ancestry-aware mapping from translation-variant SNP sequences to translation-invariant probability estimates. These probability estimates can then be processed using convolutional or sliding-window methods in the smoother module.

Optimising the window size of the classifiers results in a classic bias-variance trade-off. As the window size increases, each classifier observes more positions of the chromosome and typically achieves higher accuracy; however, it also produces lower-resolution ancestry estimates, since larger windows necessarily span longer genomic regions. Larger window sizes also increase the number of SNPs processed by each model and therefore increase memory usage. Such low-resolution estimates may subsume short ancestry segments that reflect older admixture events or span ancestry switch points. This creates a conflict during the learning process, as the model must assign a single ancestry label to a window that may contain segments from multiple ancestries. Conversely, small windows produce noisier probability estimates, leaving the task of correcting the predictions to the smoother. Furthermore, genotyping array data, which has relatively low SNP density, performs poorly with small windows because only a limited number of SNPs are available per window. To alleviate these trade-offs, we make use of overlapping windows, introducing the concept of context, *c*. For non-negative context, each base model observes *c* additional SNPs on either side of its assigned window. Thus, for a window size of *w*, each base model processes a total of *w* + 2*c* SNPs. This design allows each model to observe more SNPs without reducing the resolution of ancestry predictions along the genome.

Our system is highly modular, supporting flexible selection of supervised discriminative classifiers for both the base and smoothing modules, enabling replacements that are suited for a given task. While the Gnomix system is designed to support any type of supervised classifier, in this work, we explore the following classifier families:

- Tree-based models: Random Forest (RF), XGBoost (XGB), CatBoost (CB), and Light Gradient Boosting Machine (LGBM). These models are well suited for genomic data, handling high-dimensional, discrete, and missing inputs efficiently and accurately, while capturing non-linear dependencies induced by linkage disequilibrium.
- Linear models: Logistic Regression (LR), Linear Discriminant Analysis (LDA), and Naive Bayes variants – Gaussian Naive Bayes (GNB), Multinomial Naive Bayes (MNB), and Bernoulli Naive Bayes (BNB). These provide fast, interpretable classifiers that exploit allele frequency differences between populations to classify each window.
- Support Vector Machines and K-Nearest Neighbours: SVMs with linear, radial basis function, and string kernels, as well as our novel CovRSK, together with K-Nearest Neighbours (KNN). These similarity-based methods can accurately classify genomic sequences by comparing them to some support (training) sequences of known ancestry. The string kernel and CovRSK are particularly suited to capturing admixture and linkage disequilibrium.

### Smoother module

After the windowed SNPs are processed by the base module, the initial ancestry estimates are refined by a smoothing module (Fig. 1e–g). We introduce a fully data-driven approach that produces refined ancestry predictions using discriminative smoother models trained to correct the errors made by the base module classifiers and to learn the statistical properties of ancestry switches (recombination probabilities) along the chromosome. This is achieved without requiring explicit information about the dates of admixture events (priors on ancestry segment lengths) or other model-based assumptions. Such demographic parameters are often unknown and may vary across individuals within a cohort. Our approach differs from methods such as RFMix^15^, which require explicit specification of time since admixture and use a single constant to represent the transition probability across all population pairs. In contrast, our trained smoother allows transition probabilities to be inferred (implicitly or explicitly, depending on the smoother) for each ancestry combination and to vary across genomic windows, resulting in far more flexible modelling and eliminating the need to specify a single (typically unknown) time since admixture parameter. With the exception of the CRF, which does not use it, our smoothers share a single hyperparameter: the smoothing window size, which determines how many neighbouring base classifier windows are included as input when predicting the final ancestry estimate for a given window.

However, simply passing the training data through the base models to obtain probability estimates and then using these estimates to train the smoothing module introduces a subtle bias, namely a distributional shift at test time:

$$P({B}^{{{{\rm{tr}}}}},{Y}^{{{{\rm{tr}}}}})\ne \,P({B}^{{{{\rm{te}}}}},{Y}^{{{{\rm{te}}}}}).$$

That is, the joint distribution of the base module probabilities, *B*, and labels *Y*, generally differs between training and inference (Supplementary Figs. 1, 2), because the base module is overfitted to the training samples and thus produces overconfident predictions. To avoid this, we split the training data into two sets: one used to train the base module and one used to train the smoothing module. The smoother subset is first passed through the base module to obtain unbiased (not overfit) probability estimates that, together with the corresponding labels, better resemble the joint distribution at test time. These probabilities are then used as input to train the smoother. Although this reduces the amount of data available to each module, training the smoothing module proves to be data efficient, as it can learn across the entire genome, and we observe no performance degradation from the training data reduction (Supplementary Fig. 3). After the smoother has been trained, the base classifiers are retrained on the full training dataset to avoid any loss in base-module accuracy. The full pipeline is detailed in the Methods section.

We explore three types of data-driven smoothers:

- Linear Convolutional Filter (Logistic): A linear convolution followed by softmax activation (equivalent to convolutional multinomial logistic regression) trained with cross-entropy loss learns a smoothing filter that combines neighbouring window probabilities to produce refined predictions. Linearity ensures interpretability, and the learnt weights provide insight into the structure of the training data (see Fig. 3).
- XGBoost: Boosting is well suited to aggregating and refining probabilities. Applied in a sliding-window (convolutional) manner, the same model is shared across windows. Its nonlinearity enables modelling of more complex inter-window dependencies, leading to more accurate predictions.
- Linear-Chain Conditional Random Field: Since CRFs were used in RFMix, we revisit them as smoother functions. Here, transition probabilities are learnt by gradient descent rather than specified through priors.

### Calibration

Gnomix outputs ancestry probability estimates for each window. By selecting the ancestry with the highest probability, an ancestry label is assigned to each window. To interpret these probability estimates as confidence measures, the system must be calibrated so that the predicted probability approximates the true probability for each ancestry label. In other words, predictions assigned a probability of *X*% should be correct *X*% of the time. However, most classifiers do not satisfy this property; therefore, we include a calibration step that transforms the predicted probabilities (smoother module outputs) into calibrated probability estimates. Specifically, we use isotonic regression^29^, a non-parametric method that models the probabilities as a monotonic transformation of the raw predictions. This procedure shifts predicted probabilities closer to their true empirical values for each ancestry label. The calibration module is trained using the base model training subset (data not used to train the smoother) and evaluated on the test dataset. Quantitative evaluation metrics are provided in Supplementary Fig. 4.

### String kernels

In 2000, Lodhi et al.^27^ introduced the string kernel, an inner product in a feature space generated by all subsequences of a given length. For two sequences of length *M*, $x,{x}^{{\prime} }\in {{\mathbb{R}}}^{M}$, the string kernel is given by:

1 $$K(x,{x}^{{\prime} })={\sum}_{k=1}^{M}{S}_{k}={\sum}_{k=1}^{M}{\sum}_{i=1}^{M-k+1}I({x}_{i:i+k}={x}_{i:i+k}^{{\prime} })$$

where *S*_*k*_ is the number of matching subsequences of length *k* where *x* and ${x}^{{\prime} }$ are equal, i.e., the total number of shared *k*-mers. This kernel has proven particularly suitable for genome sequence comparison, including applications to LAI^14,30^. Efficient computation of the string kernel, along with its polynomial generalisation, is described in the Supplementary Discussion and illustrated in Supplementary Figs. 8 and 9.

### String kernel regularisation

Despite its advantages, the string kernel exhibits computational inefficiencies and statistical weaknesses. In particular, by viewing the subsequence equality counts *S*_*k*_ as features generating the kernel feature space, it can be shown that these features are highly correlated under mild assumptions. Let *q* denote the probability that an input feature is identical for two individuals. The kernel features can then be modelled recursively as

$${S}_{k}| {S}_{k-1} \sim \,{\mbox{Binomial}}\,({S}_{k-1},q).$$

The covariance between two kernel features satisfies

$${{{\rm{Cov}}}}({S}_{k},{S}_{k+j})={q}\,^{j}{{{\rm{Var}}}}({S}_{k}),$$

which yields the lower bound

$${\rho }_{{S}_{k},{S}_{k+j}}\ge {q}\,^{j+1}.$$

See Supplementary Eqs. (1)–(3) for details. In genomic sequences, *q* is often close to one, resulting in strong correlations between neighbouring kernel features.

Moreover, the variances of the string kernel features decrease dramatically with the subsequence length *k* (Supplementary Fig. 10), reducing the marginal contribution of longer subsequences and increasing redundancy. Larger subsequences are therefore less likely to influence the similarity measure. Given the high correlation between neighbouring string kernel features, the effectiveness of a given feature also depends strongly on the inclusion of neighbouring features (Supplementary Fig. 11). Motivated by these observations, we introduce a modified version of the string kernel for this application, which we call the covariance-reducing string kernel (CovRSK).

CovRSK uses dynamically weighted sampling to select features so as to reduce redundancy and feature correlation. The sampling procedure starts with an empty set and, in increasing order, adds a feature *k* with probability:

$${\theta }_{\alpha,\beta }(k,{k}^{{\prime} })=(1-{\alpha }^{k-{k}^{{\prime} }+1}){k}^{-\beta }.$$

Here, *k*^−*β*^ reflects the decay in feature variance, and the first term, $(1-{\alpha }^{k-{k}^{{\prime} }+1})$, acts as a discount proportional to the lower bound on the correlation between the last selected feature and the candidate feature, ${\rho }_{{S}_{k},{S}_{k+j}}$. Both terms serve the same purpose: making more redundant features less likely to be selected. The hyperparameters *α*, *β* ∈ [0, 1] control the strength of covariance reduction and the number of features sampled, respectively. It should be noted that CovRSK generalises the original string kernel, which is recovered when *α* = *β* = 0. The method is described in more detail in Algorithm 1.

Including spatially correlated string kernel features not only introduces redundancy but also promotes overfitting to longer matches, making the model sensitive to sequencing errors that disrupt such matches. Motivated by the variance decay and correlation structure of string-kernel features (Supplementary Figs. 10 and 11), we demonstrate that CovRSK acts as a regulariser of the string kernel and improves robustness to noise arising from genotyping call errors (Supplementary Figs. 12 and 13). An example of the sampling procedure is shown in Supplementary Fig. 14.

#### Algorithm 1

CovRSK 

### Experimental results

**Fig. 2:**
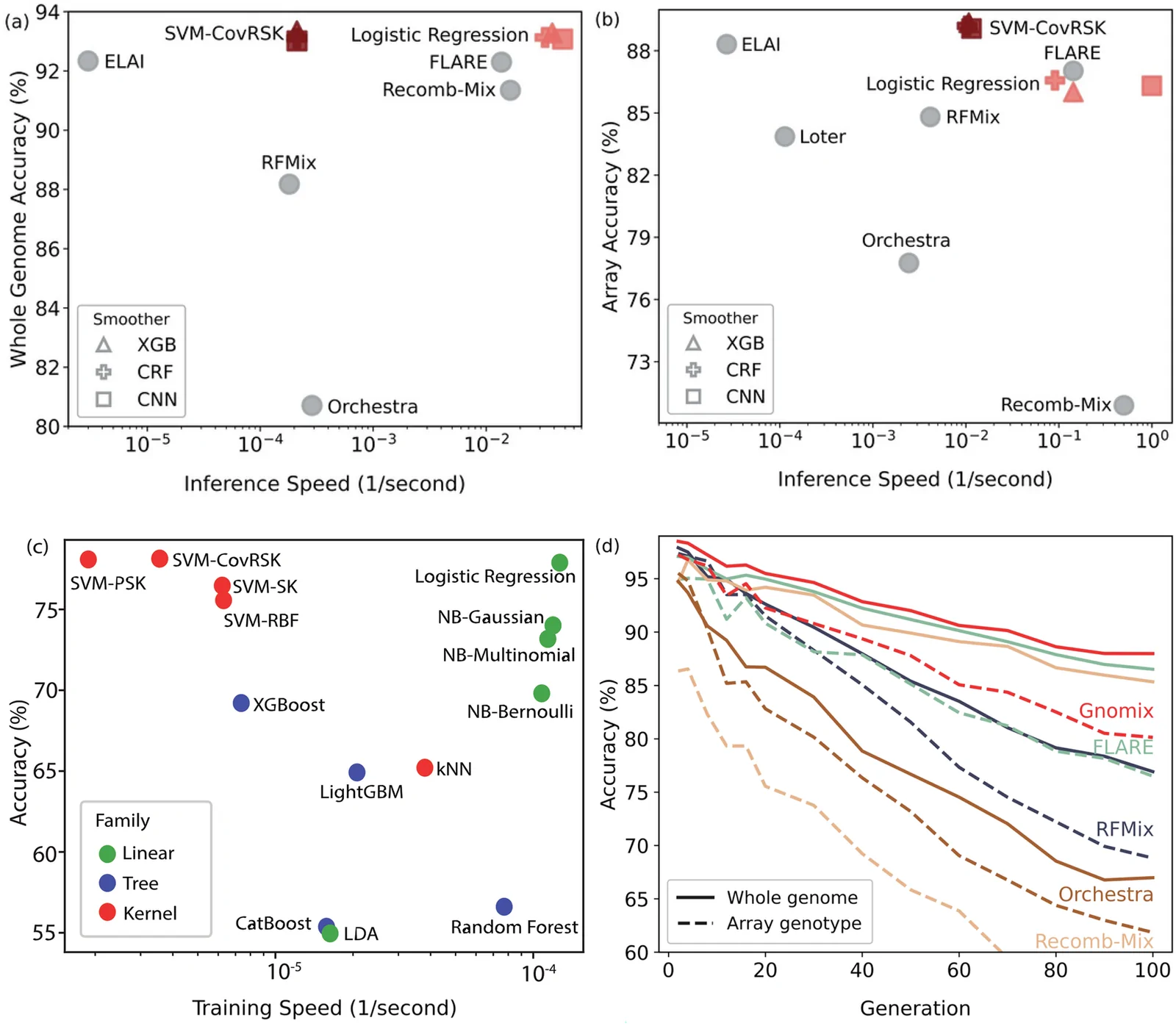
Performance plots. **a** Performance on whole-genome data for the 7-ancestry cohort. Accuracy and inference speed are averaged across all samples and generations. All Gnomix configurations are shaded in dark and light red, corresponding to SVM-CovRSK and Logistic Regression base models, respectively, with the smoother indicated by marker shape, while existing approaches are shaded in grey. Loter could not run on the whole genome due to excessive memory requirements. **b** Performance on array genotyped data for the 7-ancestry cohort. Same analysis as in (**a**), for models trained using only the SNPs found on the UK Biobank Axiom genotyping array. **c** Base model comparison on whole-genome data. Comparison of Gnomix base classifiers by accuracy, training speed, and inference speed on whole-genome data. Green points denote linear models, blue points denote tree-based models, and red points denote kernel methods. A Pareto-optimal frontier is described by SVM-CovRSK and logistic regression, representing the high-accuracy and high-speed extremes, respectively. **d** Whole genome (solid lines) and array (dashed lines) accuracy into the past. The accuracy of our best Gnomix algorithm, CovRSK SVM with XGBoost smoother, is compared against RFMix, FLARE, Recomb-Mix, and Orchestra as a function of the number of generations since admixture. Larger numbers of generations since admixture generate more ancestry switches and shorter average ancestry segments, leading to a more challenging problem. Gnomix achieves the highest accuracy across both whole-genome and array data, with performance advantages becoming more pronounced for admixture further in the past.

For thoroughness and a fair comparison to existing methods, we generated multiple continental-admixed datasets with varying numbers of ancestral populations, which we denote as the Latin American, 5-ancestry, and 7-ancestry cohorts (Supplementary Tables 1 and 2). For each cohort, we report classification accuracy, training time, and inference time across all model configurations. The primary experiments were conducted using whole-genome sequence data from chromosome 20. In addition, we subsampled variants corresponding to positions represented on a commonly used genotyping array (specifically, the UK Biobank) to generate a second benchmark dataset (array genotyped). We compare all of our results with the most widely used current LAI methods, including RFMix (Random Forests)^15^, ELAI (HMMs)^16^, Loter (dynamic programming)^17^, FLARE (HMMs)^21^, Orchestra (neural-network)^20^, and Recomb-Mix (graph optimisation)^22^. We first evaluated a broad set of base classifiers and selected SVM-CovRSK and logistic regression for the main experiments, as they provided the best accuracy and accuracy–speed trade-off, respectively (Fig. 2c). Across datasets and resolutions, Gnomix delivers the strongest accuracy overall. On whole-genome data, all Gnomix configurations achieve the highest or near-highest accuracy while remaining computationally competitive. On array data, CovRSK configurations achieve the best accuracy, while the logistic regression configuration remains highly accurate with much faster inference. These trends are consistent across all datasets and resolutions (Table 1). Detailed benchmarking results (covering accuracy, log-loss, base-model performance, and training and inference times across all configurations and datasets) are provided in Supplementary Tables 4–9. In some benchmarks, ELAI and Loter could simply not be run due to their prohibitively high computational requirements.

**Table 1:** Benchmarking performance across datasets and resolutions

	Whole genome						Array genotype					
	Latin American		5-ancestry		7-ancestry		Latin American		5-ancestry		7-ancestry	
Model	Accuracy (%)	Time (s)	Accuracy (%)	Time (s)	Accuracy (%)	Time (s)	Accuracy (%)	Time (s)	Accuracy (%)	Time (s)	Accuracy (%)	Time (s)
CovRSK-crf	97.63	1933	95.16	5004	93.10	4731	96.55	32	91.85	86	89.20	97
CovRSK-xgb	97.70	1932	**95.40**	5001	**93.35**	4727	**96.67**	31	**92.06**	83	**89.35**	93
CovRSK-cnn	97.29	1931	95.07	4997	93.01	4722	96.36	29	91.72	79	89.07	87
logreg-crf	97.69	14	95.19	23	93.13	30	96.23	8	90.08	15	86.57	11
logreg-xgb	97.79	13	**95.40**	20	93.28	26	96.30	7	89.99	13	86.01	7
logreg-cnn	97.29	**12**	95.09	**17**	93.07	**21**	95.90	6	90.00	9	86.31	**1**
rfmix	95.94	1594^*^	90.76	4261^*^	88.18	5518^*^	95.58	82^*^	90.28	196^*^	84.81	241^*^
elai	–	–	–	–	92.34	334003^*^	–	–	–	–	88.32	37219^*^
loter	–	–	–	–	–	–	–	–	–	–	83.86	8840^*^
FLARE	97.74	26	94.92	57	92.30	73	95.69	4	90.24	7	87.03	7
Orchestra	90.35	1526	87.47	3436	80.71	3481	90.81	158	83.17	266	77.76	410
RecombMix	**97.92**	22	94.76	48	91.35	61	90.88	**1**	75.57	**1**	70.89	2

Classification accuracy (%) and inference time (seconds) are reported for Gnomix configurations and existing LAI methods across three simulated admixed cohorts – Latin American, 5-ancestry, and 7-ancestry – using both whole-genome and array-genotype data. Boldface indicates the best value in each metric column. Asterisks (*) in the runtime columns denote combined training and inference time for methods that do not support standalone inference from previously trained models. Separate training times for all methods are reported in Supplementary Tables 4–9. Missing accuracy values indicate benchmarks in which ELAI or Loter could not complete due to prohibitively long computational runtimes.

Figure 2c compares the accuracy, training time, and inference time of the different Gnomix base classifiers on the whole-genome development dataset (Supplementary Tables 1 and 2): RF, CB, XGB, LGBM, KNN, SVM with radial basis function (SVM-RBF), SVM with string kernel (SVM-SK), SVM with polynomial string kernel (SVM-PSK), SVM with CovRSK (SVM-CovRSK), LDA, LR, MNB, BNB, and GNB. SVM with CovRSK achieves the highest accuracy, although with long training and inference times. Polynomial string kernel and logistic regression follow closely in accuracy, with logistic regression being the fastest. Logistic regression surpasses other linear models, including LDA and Naive Bayes, and has the fastest training and inference time of all models. The gap between Naive Bayes, which assumes independence between features, and logistic regression, which does not, highlights the importance of accounting for the correlation (linkage) between SNPs. Similarly, the superior performance of string kernels over purely linear models indicates the importance of modelling local sequence dependencies, particularly in lower-resolution array data. Tree-based methods exhibit a wide range of accuracies and training/inference times. RF performs substantially worse than boosting methods, while XGBoost provides the most accurate predictions among the tree-based methods, at the cost of higher training and inference time. Overall, kernel and linear methods outperform tree-based approaches, and linear models offer considerably lower computational cost than tree-based or kernel models. Based on these results, we highlight two Gnomix base classifiers with different accuracy/speed trade-offs: SVM with CovRSK as the most accurate – though slower – classifier, and logistic regression as an accurate, interpretable, and computationally efficient alternative.

Figure 2a (whole genome) and Fig. 2b (genotyped array data) compare accuracy and inference speed on the 7-ancestry cohort, with accuracy averaged across all samples and generations. These panels include Gnomix configurations combining different smoothing modules (CRF, CNN, and XGBoost) with either SVM-CovRSK or logistic regression as base classifiers, together with recent and popular LAI methods: RFMix, Loter, ELAI, FLARE, Orchestra, and Recomb-Mix. We observe that the CNN smoother is generally the fastest, while XGBoost typically achieves the highest accuracy, particularly in combination with CovRSK and on feature-dense whole-genome data. Additionally, while CovRSK is substantially slower than logistic regression on whole-genome data, this gap narrows on genotyped array data. Across both whole genome and array settings, our CovRSK-XGBoost achieves the highest accuracy overall. While this configuration can require longer training time than RFMix in some settings, lighter configurations such as logistic regression with CRF achieve near-matched accuracy with substantially higher throughput (Supplementary Tables 4–9). All Gnomix configurations substantially surpass RFMix and Orchestra in accuracy at both resolutions. Gnomix exceeds FLARE in accuracy with both CovRSK and with logistic regression configurations, while offering faster inference than FLARE when using the logistic regression configuration paired with any smoother (CNN, CRF, or XGBoost). Gnomix also exceeds Recomb-Mix in average accuracy while maintaining highly competitive inference speeds, with the Gnomix advantage becoming especially pronounced as the number of ancestries increases, a regime in which Recomb-Mix struggles (Table 1). On array data, CovRSK configurations of Gnomix achieve the highest accuracy overall across all competitors, while logistic regression configurations remain competitive (surpassed only slightly by FLARE) and retain the fastest or near-fastest inference times. In contrast, Recomb-Mix performance degrades dramatically on array data. Training times for all methods are reported in Supplementary Tables 4–9. Importantly, once trained on a set of references, Gnomix models can be reused on new cohorts with the same variants, amortising the initial training cost and rendering the models, in this sense, even faster computationally than those of methods that must be re-trained whenever they are run.

We note that CovRSK provides a significant improvement over logistic regression on genotyping array data, whereas on whole-genome data both methods perform similarly. This suggests that with enough SNPs-per-window – that is, sufficient input features for each base classifier – linear models such as logistic regression perform extremely well, while more computationally complex non-linear techniques, such as CovRSK, provide only marginal additional improvement. In contrast, when SNP density is lower, as in array data, non-linear models that generate richer feature spaces, such as CovRSK, provide a significant improvement in accuracy.

Robustness to the generation time since admixture is another important aspect of LAI methods. The generation time will determine the length distribution of the segments originating from each ancestry group, so it is important that methods can properly deal with a wide range of generation times and length distributions. Fig. 2d compares our top-performing configuration (CovRSK SVM with XGBoost smoother) against RFMix, FLARE, Orchestra, and Recomb-Mix over a wide range of generation times. (These are the only alternative methods that combine decent accuracy with practical run times on large, whole-genome datasets, as shown above.) Solid lines denote whole-genome data, and dashed lines denote array-genotyped data. As expected, accuracy decreases across all methods as the generation number increases, making ancestry segments shorter. However, Gnomix shows the slowest decline in both whole genome and array accuracy, maintaining the highest accuracy into the past across all methods and remaining above 90% accuracy well beyond 100 generations (approximately 3000 years ago for humans). RFMix and Orchestra decrease rapidly in accuracy as generation number increases, while FLARE and Recomb-Mix decline more gradually on whole-genome data. On array data, however, Recomb-Mix shows a sharp loss of accuracy with generation. Notably, Gnomix performance on array data is so accurate that it surpasses the performance even of RFMix and Orchestra on feature-rich whole-genome data.

**Fig. 3:**
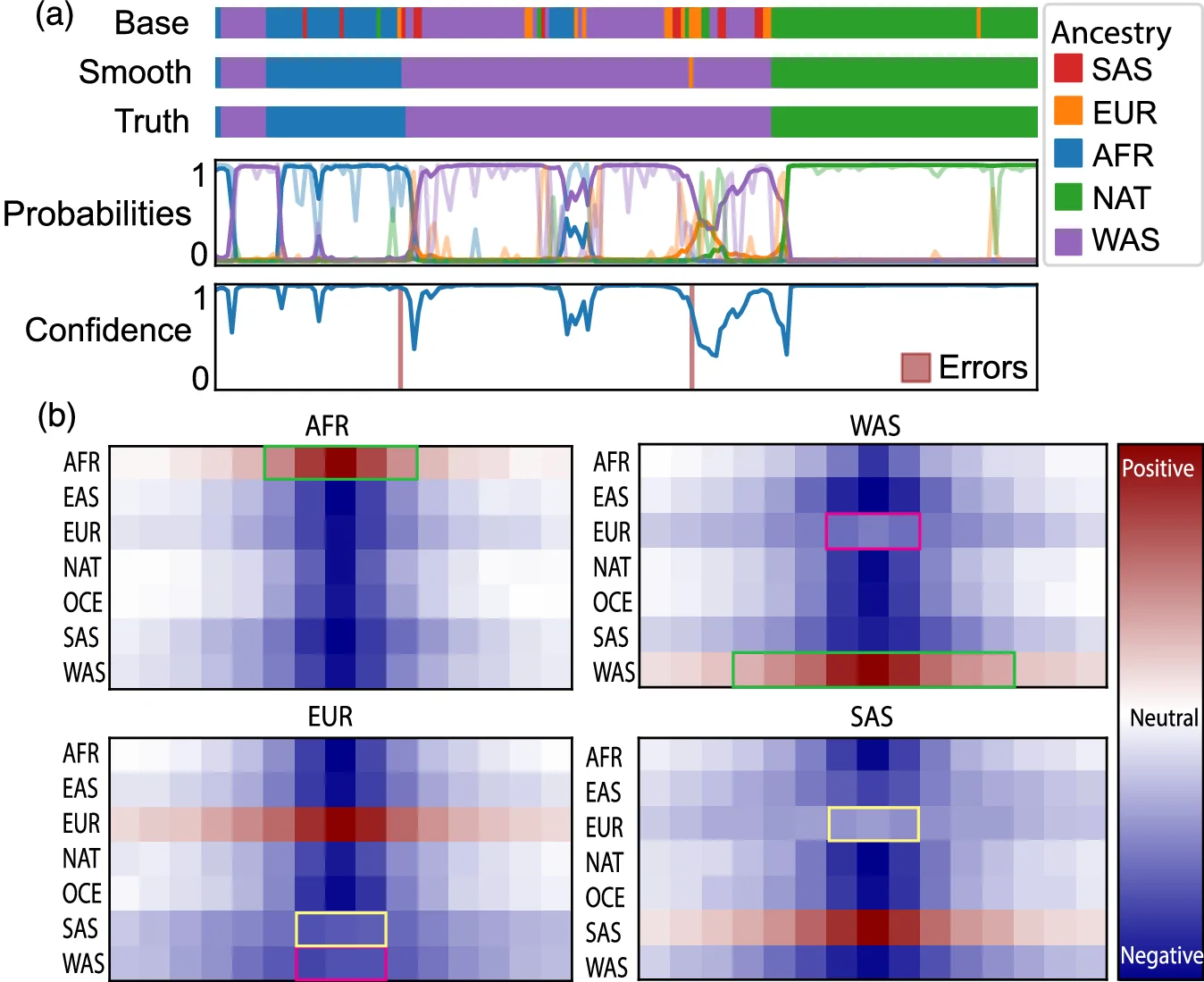
Effect of population structure on learnt models. **a** Predicted probabilities and errors of Gnomix on a sample admixed haplotype. Base and smoother probabilities use the same ancestry colours, with base probabilities faded. The model makes two errors: one next to an ancestry switch point and one where a small West Asian (WAS) segment is classified as European (EUR). **b** Weights learnt by the convolutional smoother for base-model ancestry predictions, with ancestries on the vertical axis, windows on the horizontal axis, and target ancestries shown in the plot titles. Red and blue indicate positive and negative importance, respectively. The model is trained on seven populations: African (AFR), East Asian (EAS), European (EUR), Indigenous American (NAT), Oceanian (OCE), South Asian (SAS), and West Asian (WAS). The African smoother needs few surrounding African window probabilities (green rectangle, upper left), reflecting clearer differentiation from other ancestries, whereas the West Asian smoother requires broader context because WAS is less well differentiated from EUR (green rectangle, upper right). Shared introgression (perhaps Early Farmer^31^) between West Asian and European ancestries results in only weakly negative weights for European probabilities in the West Asian smoother (pink box, upper right). Consequently, the model must integrate information across a broader span of neighbouring windows to accumulate sufficient discriminatory signal. A similar pattern is observed for European and South Asian ancestries (yellow box, lower right), likely reflecting shared Indo-European-related introgression^32^. More generally, closely related ancestries require longer ancestry segments for reliable differentiation.

Furthermore, while some closely related populations, such as West Asian and European, pose a significant challenge for methods like RFMix (Supplementary Fig. 6), Gnomix handles these far better because its base models and non-linear data-driven smoothers are more accurate (Fig. 3). In addition to being able to handle multiple generation time regimes (Fig. 2d) and related ancestries, Gnomix is also robust to the population set on which it is trained. To assess the importance of the specific samples used to construct the reference panel, we created an additional Latino dataset in which the European populations used for training differed from those used for testing. That is, Southern European populations, including Iberian and Italian, were used for testing and Northern European populations, including Finns and British, were used for training. In this heterogeneous setting, which is often encountered in practice, Gnomix achieved 97.55% accuracy compared to the nearly identical 97.69% in our main experimental configuration, which had used identical European subpopulations in both training and testing. This negligible difference indicates that Gnomix is robust to moderate reference panel mismatch and does not overfit to particular subpopulation structures. The same cannot be said for the other methods.

### Population structure is reflected in the learnt models

The effect of the smoother for an example haplotype is shown in Fig. 3a. Here, the model makes errors on two segments that both – in their own way – shed light on the typical errors that can occur. On the left, the model has difficulty determining the switch points (the boundary between two ancestries). Indeed, by looking at Supplementary Fig. 5, it is clear that model accuracy increases rapidly with distance from a switch point, with most errors occurring at or around these points. The error on the right is a West Asian segment wrongly classified as European. This phenomenon appears again in the discussion of Fig. 3b below, where the similarity of these two related populations plays a role in confusing the classifier. Indeed, given these two ancestries’ shared historical introgression from early farmers in the Middle East, this European prediction might reflect genuine shared ancestry. When observed closely, the independent base probabilities appear to be of similar magnitude for both West Asian and European in this segment, further supporting this hypothesis.

Analysing the weights learnt by the linear convolutional model, which indicate how the smoother module aggregates information from the base models’ outputs, we gain insight into the data-driven smoother in Fig. 3b. By learning a linear filter in a data-driven way, the magnitude of its weights can be interpreted as a measure of feature importance. The filter weights show that, when predicting ancestry in a window, the smoother relies strongly on the base models’ priors. There is a strong positive correlation between base model probability estimates and the smoother predictions of a given ancestry (weights coloured red). Furthermore, the spread of positively correlated windows is larger for populations that are less well separated, like West Asian (WAS) and European (EUR), compared with population labels that are well separated, such as African (AFR). Examining the centre columns of the European (EUR) filter, we find that a high EUR base probability is positively correlated with being predicted European (red), but negatively correlated (dark blue) with being predicted AFR (African), OCE (Oceanian), EAS (East Asian), and NAT (Indigenous American). Interestingly, EUR is less negatively correlated (lighter blue) with predicting West Asian (WAS) and South Asian (SAS) than with any of the other ancestries. This likely reflects shared historical introgression events with Europeans, namely the spread of early Near Eastern farmers in the case of WAS^31^, and the spread of Indo-European languages in the case of SAS^32^. This effect is not observed between South and West Asians in our dataset, suggesting that the common ancestor between European and West Asian references, namely Neolithic Anatolian farmers, differs from that between South Asian and European references, namely Indo-European speakers.

The presence of such shared population histories adds complexity to this inference task and can help explain the success of nonlinear smoothers. We also note that choosing reference samples and label names in this context is not always simple and depends on the time period being investigated. Although some continental populations have long been separated, diverging through independent genetic drift^33^, no human population is an “island" without historical introgression, admixture, and shared ancestry with others^34^. Care must therefore be exercised when choosing label names for genetic clusters to avoid conflating them with socially constructed ethnicities^35^, whose boundaries can vary rapidly across genetic space and across time via language shifts, religious conversions, and the formation of newly shared (or divided) identities during modern nation-building.

### Phasing error correction: Gnofix

Because most sequencing technologies in use are unable to assign an accurate parental chromosome source to each sequencing read in a diploid genome, several statistical algorithms have been developed to assign each variant to its correct parental haplotype. These techniques, known as phasing, take advantage of observed correlations between neighbouring variants in databases of reference populations. Some of the most commonly used methods include Beagle^36^ and SHAPEIT^37^. While these tools are highly accurate locally – that is, the probability of two neighbouring variants being correctly phased is very high – they occasionally fail, producing swaps between the two parental sequences. Thus, the probability that two distant variants are correctly phased converges to random chance as their separation increases. Fortunately, such errors can often be corrected with LAI. Given the additional information present in ancestry calls along the genome, such sporadic switch errors become apparent. The most commonly used phasing error correction method using LAI that can handle multiple ancestries is found in RFMix (version 1, but not version 2). Such long-range ancestry-block phasing is crucial when investigating demographic models that use contiguous ancestry block lengths (tracts) as an input^38^.

We propose a novel method, Gnofix, that employs a trained smoother from the Gnomix module to find the most likely phase assignment of an individual’s two haplotypes. This is done by using the confidence of the ancestry estimates for a given sequence as a proxy for the probability that the sequence comes from the observed distribution of human haplotypes. Starting with window-based ancestry probability estimates for an individual’s maternal and paternal haplotypes (phased using an existing algorithm), these probabilities are leveraged to iteratively swap segments between the two haplotypes until the most probable configuration is recovered. Such input probabilities can be obtained from many LAI models, including RFMix^15^, LAMP^13^, LAI-Net^18^ and, of course, Gnomix.

**Fig. 4:**
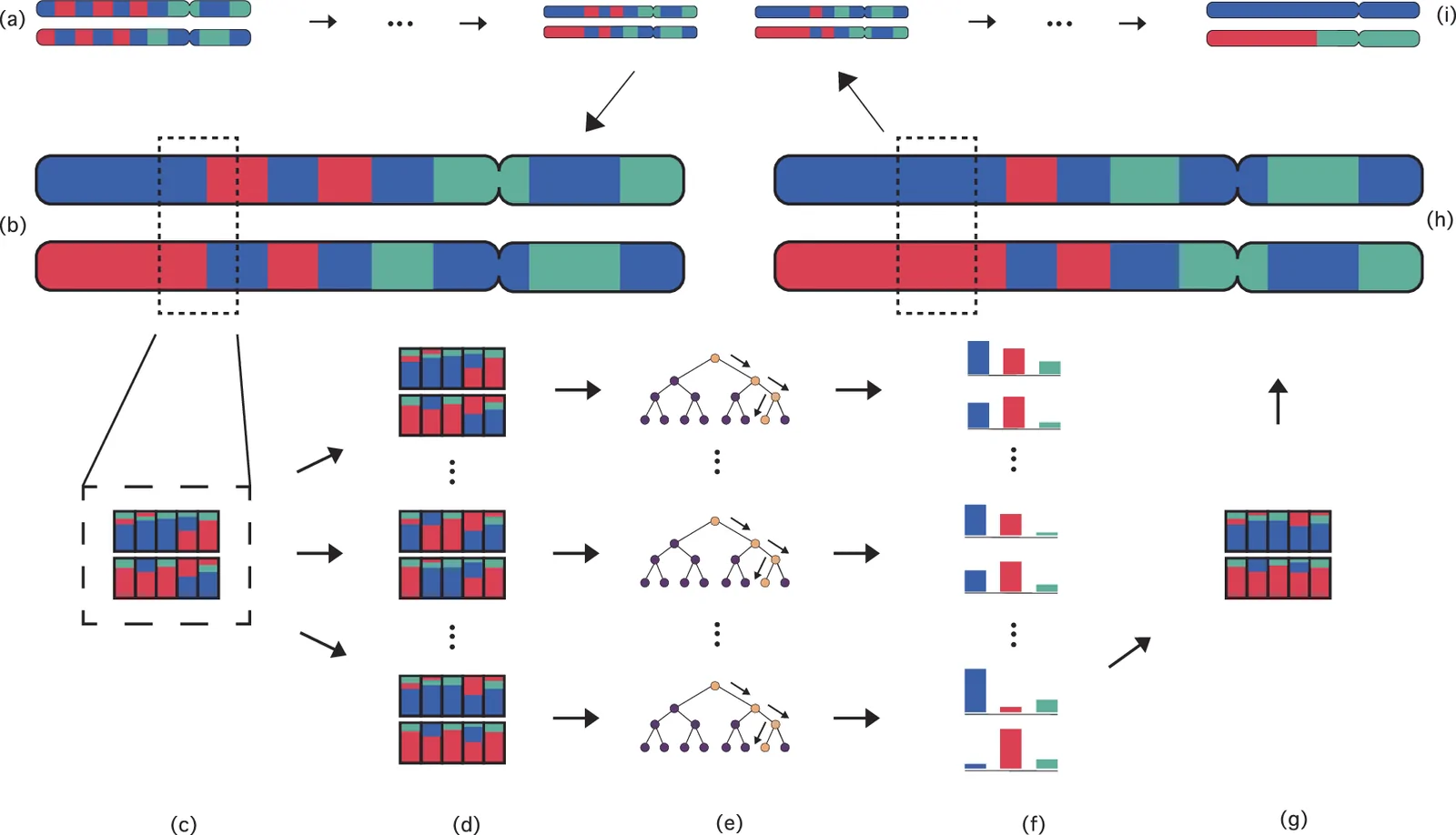
Gnofix phase correction algorithm. **a** Input consisting of maternal and paternal haplotypes containing phasing errors. The colours denote different ancestry predictions. **b** Appearance of the haplotypes at a given state and after some phasing errors have been corrected. The dashed box marks the scope centred on the current window. **c** Probabilities of each ancestry at each window within the scope are extracted. **d** Collections of swaps are proposed. **e**, **f** Proposals are passed through the smoother to obtain predictions and confidence. **g** The proposal with the highest confidence is chosen. **h** Windows in scope are returned back to the haplotypes. **i** Output maternal and paternal haplotypes containing no phasing errors.

The algorithm starts from one side of the chromosome and, during one iteration, works its way to the other. At each step, it defines its scope as a set of local consecutive windows with the same length as the trained Gnomix smoother size. It then extracts the ancestry probabilities within this scope and explores different local phasing alternatives by exploring potential combinations of swaps between the two parental sequences. This results in a collection of swap combinations, each treated as a proposal for the true sequence. The collection need not contain the complete set of all possible swap combinations, however. Indeed, doing so would create redundancy, since neighbouring scopes have a large overlap for swap proposals. In addition, for any sizeable smoother size, exhaustive enumeration of all swap combinations would be intractable. Thus, the collection of swap combinations is restricted to include only swaps at – or closely around – the centre, where the smoother is most receptive; this reduces the number of proposals dramatically. Once extracted, these proposals are passed into the smoother to obtain predictions for the centre window and – more importantly – prediction confidences. The proposal with the highest confidence is then selected, and the scope is shifted along the haplotype to analyse the next position. Once the full chromosomal sequence has been traversed, the scope is shifted back to the start, and the next iteration begins. Iterations continue until convergence or until a chosen upper bound is reached. A general outline is shown in Algorithm 2, and the process is visualised in Fig. 4.

#### Algorithm 2

Gnofix 

**Fig. 5:**
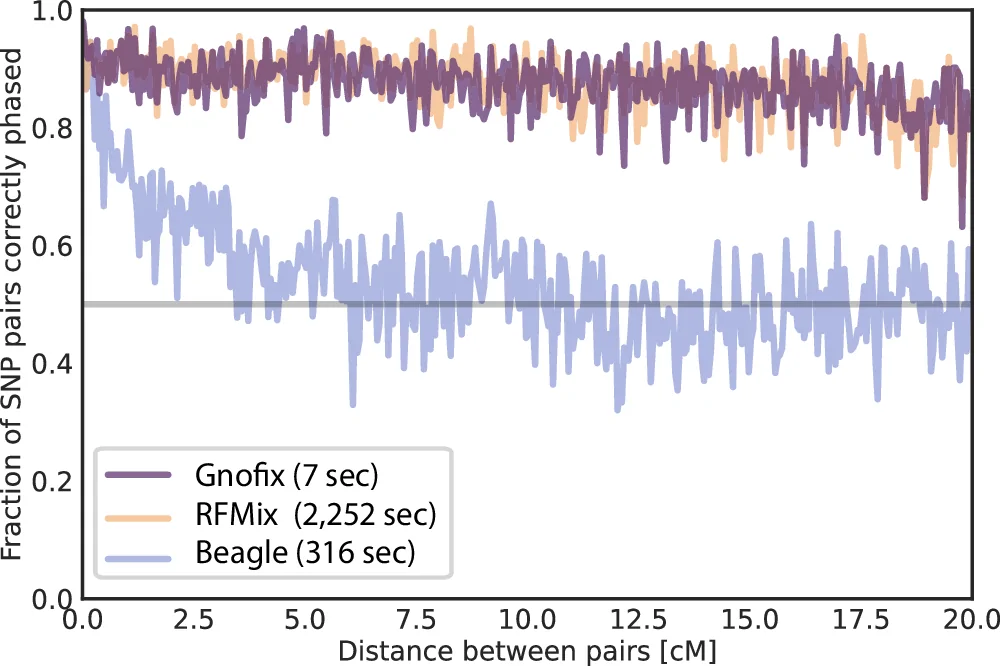
Re-phasing performance. Fraction of SNP pairs correctly phased as a function of genetic distance between pairs in the Latin American phasing dataset. Gnofix achieves accuracy comparable to RFMix while running substantially faster, and both methods maintain high long-range phasing accuracy compared with Beagle alone; indeed, the latter approaches the random-phasing baseline of 0.5 at larger distances.

We compare the performance of Gnofix to Beagle phasing and to rephasing using RFMix by plotting the fraction of SNP pairs correctly phased as a function of genetic distance between them, where SNP pairs are sampled randomly from heterozygous ancestry regions (homozygous ancestry regions cannot be phase-corrected using local ancestry). When the data is not well phased, we expect this fraction to decrease with genetic distance and converge to 0.5 (random chance). We perform this comparison on a Latin American phasing dataset created by simulating European, Native American, and African three-way admixture. RFMix and Gnofix show similar phase-correction accuracy (Fig. 5); however, their run times differ by several orders of magnitude. Beagle and RFMix take about 5 and 37 minutes, respectively, to run on this dataset, whereas Gnofix takes only 7 seconds. Such acceleration becomes crucial for large biobank datasets, for which RFMix-style phase correction becomes infeasible. See Supplementary Algorithm 4, Supplementary Table 3, Supplementary Table 10, and Supplementary Fig. 7 for details on the phasing dataset construction and additional runtime analyses.

### Application to new world dogs

As a real-world demonstration of the efficiency and robustness of our approach, we trained a Gnomix model on whole-genome sequences from 191 canids from Plassais et al.^28^ to annotate canid species and breed along the genome (see Methods for more details). We then applied it to 531 dog genomes from the same study and finally used Gnofix to correct phasing errors.

**Fig. 6:**
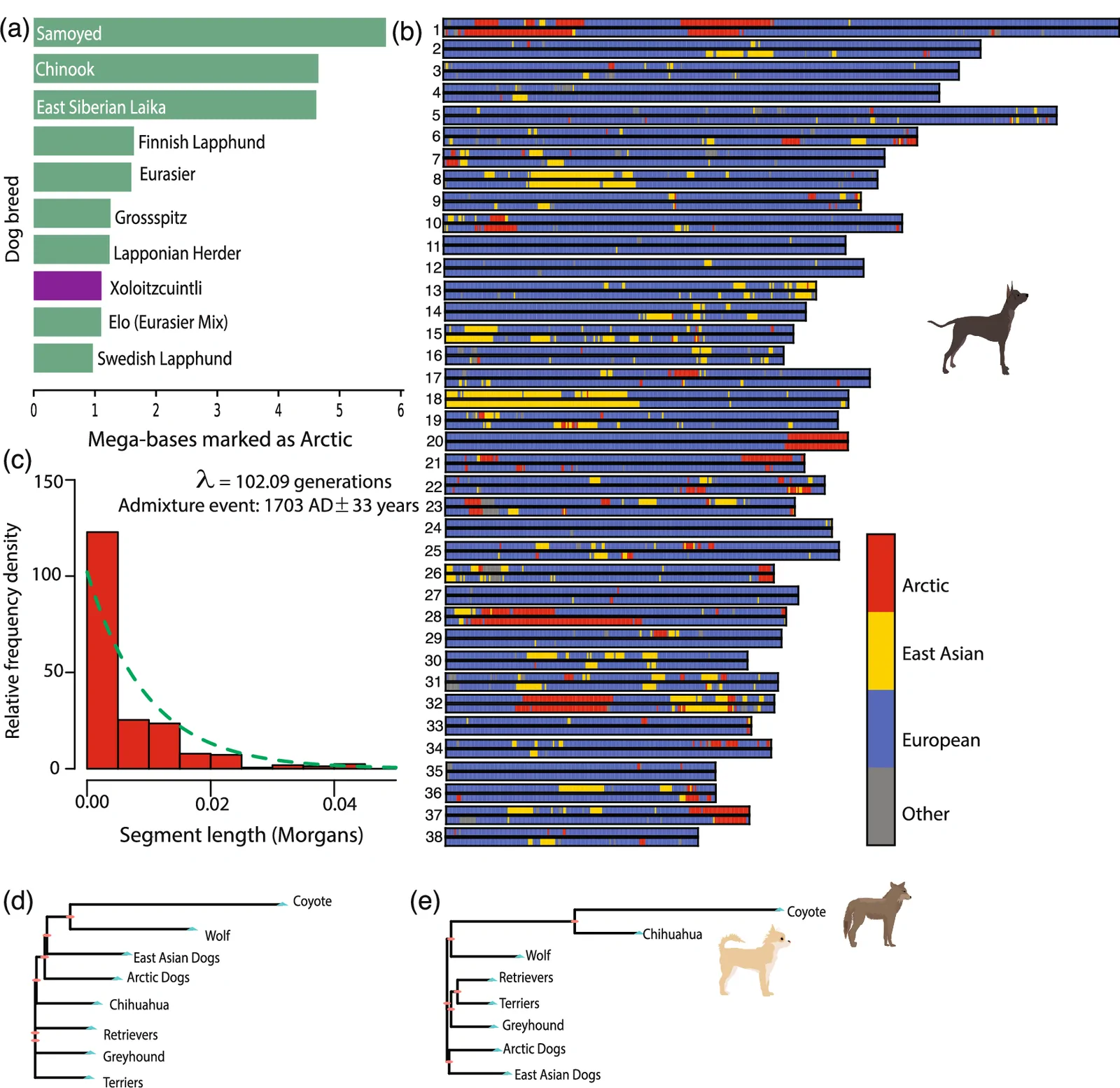
Gnomix applied to dogs reveals ancient New World admixture. **a** The ten breeds with the highest total Arctic segment ancestry. **b** Chromosome painting of a Xoloitzcuintli (Xolo) dog. **c** Segment length distribution of Arctic segments in the Xolo. **d** Neighbour-joining tree for chromosome 1 across various canids using the average number of pairwise differences (*π*). **e** Neighbour-joining tree of *π* between coyote segments identified in the chihuahua by local ancestry inference versus sequences of other canids.

We sorted the local ancestry calls from each genome by the total length of Arctic-breed segments called per sample and visualise the top ten dogs (Fig. 6a). We observe that all of these dog breeds, except for the Xoloitzcuintli (Xolo), belong to Arctic breeds or breeds known to be admixed with them (Spitz). Similarity in ancestry between Arctic breeds and Native American dogs has been noted previously; indeed, pre-contact Native American dogs lie closest to modern Arctic breeds in trees constructed from ancient genomes^39^. Thus, Arctic local ancestry models can also detect ancestry segments deriving from Native American dogs. The segments identified in the Mexican Xoloitzcuintli, which is said to have descended from pre-Columbian Mesoamerican dogs, may therefore originate from the pre-Columbian dogs kept by ancient Mesoamerican cultures (Fig. 6b). The total length of Arctic ancestry segments that we identify in the Xolo amounts to 4.03% of its genome, which matches prior global ancestry estimates placing the Xolo’s pre-Columbian ancestry at approximately 3%^40^. To our knowledge, we are the first to identify this pre-Columbian ancestry at the genomic segment level.

Under a standard recombination model, local ancestry segment lengths follow an approximately exponential distribution^38^, with the rate parameter directly related to time since admixture. Fitting an exponential curve to the distribution of the Arctic genomic ancestry segments in the Xolo (Fig. 6c), and using a dog generation time of 3 years^41^, gives an average admixture time of 306 years. This implies an average admixture date in Mexico between European and pre-Columbian dogs of 1703 AD  ± 33 years, closely matching the estimated admixture date between European and pre-Columbian peoples in Mexico of 1746 AD (8.6 generations with 30 years/generation), reported by Price et al. using a comparable single-pulse admixture model^42^.

Another signature of New World dog ancestry comes from genetic introgression with the coyote^40^, a related canid (Fig. 6d) found only in the Americas. There is evidence that pre-Columbian cultures cross-bred coyotes with dogs^39^, and we indeed find shared genomic segments between coyotes and the chihuahua, another breed with links to the Americas. In Fig. 6e we display a neighbour-joining tree for the average number of pairwise differences (*π*) of sites called “coyote” in a chihuahua’s chromosome compared against other dogs.

The capacity of Gnomix to identify and annotate short, ancient chromosomal segments in whole genomes with high resolution is unmatched by existing local ancestry algorithms (see Fig. 2d) and here allows us to unravel the history of New World ancestry in Mexican dog breeds at generation times (one hundred generations) that in humans would correspond to demographic events occurring more than 3000 years ago. In addition to surpassing the capabilities of current LAI algorithms, our method also requires only minutes – rather than days – to run on whole genomes.

## Methods

### Human datasets

We source our worldwide human whole-genome data from three publicly available projects: 1000 Genomes^43^, Human Genome Diversity Project (HGDP)^40^, and Simons Genome Diversity Project (SGDP)^44^. We bring all data to human genome build 37 using liftOver with UCSC chain files, retain only biallelic sites, and phase using Beagle 5.1^36^ with default parameters, without a reference panel and using a sex-averaged genetic map. Second-degree and closer relatives are removed using KING. We then filter these samples to obtain 1,380 single-ancestry samples by running unsupervised ADMIXTURE clustering^45^, defining single-ancestry individuals as those with ≥95% assignment probability to a single ancestry cluster. These steps constitute the primary preprocessing and quality control prior to applying the method, while variant harmonisation between the reference panel and query samples may also be required. In general, we would not recommend linkage disequilibrium pruning of the reference panel, as Gnomix is designed to leverage local haplotype structure and characteristic correlation patterns between nearby SNPs. In addition, denser variant sets enable the use of smaller windows and improve ancestry resolution. Accordingly, we recommend using as many high-quality, well-harmonised SNPs as practical for training. From the resulting set of single-ancestry samples, eight well-supported ancestry clusters were selected by cross-validation: East Asian (EAS), European (EUR), Indigenous American (NAT), Oceanian (OCE), South Asian (SAS), West Asian (WAS), Sub-Saharan African (AFR), and African Hunter and Gatherer (AHG). We retained only the first seven ancestry groups for our experiments, since AHG did not have enough training samples in our dataset for robust benchmarking. Reference sample size requirements depend on genetic divergence between populations. For ancestries separated by larger *F*_ST_ values or longer divergence times, fewer reference samples are typically sufficient due to stronger allele frequency differentiation. Conversely, closely related populations require larger reference panels to capture subtle haplotype structure differences.

**Fig. 7:**
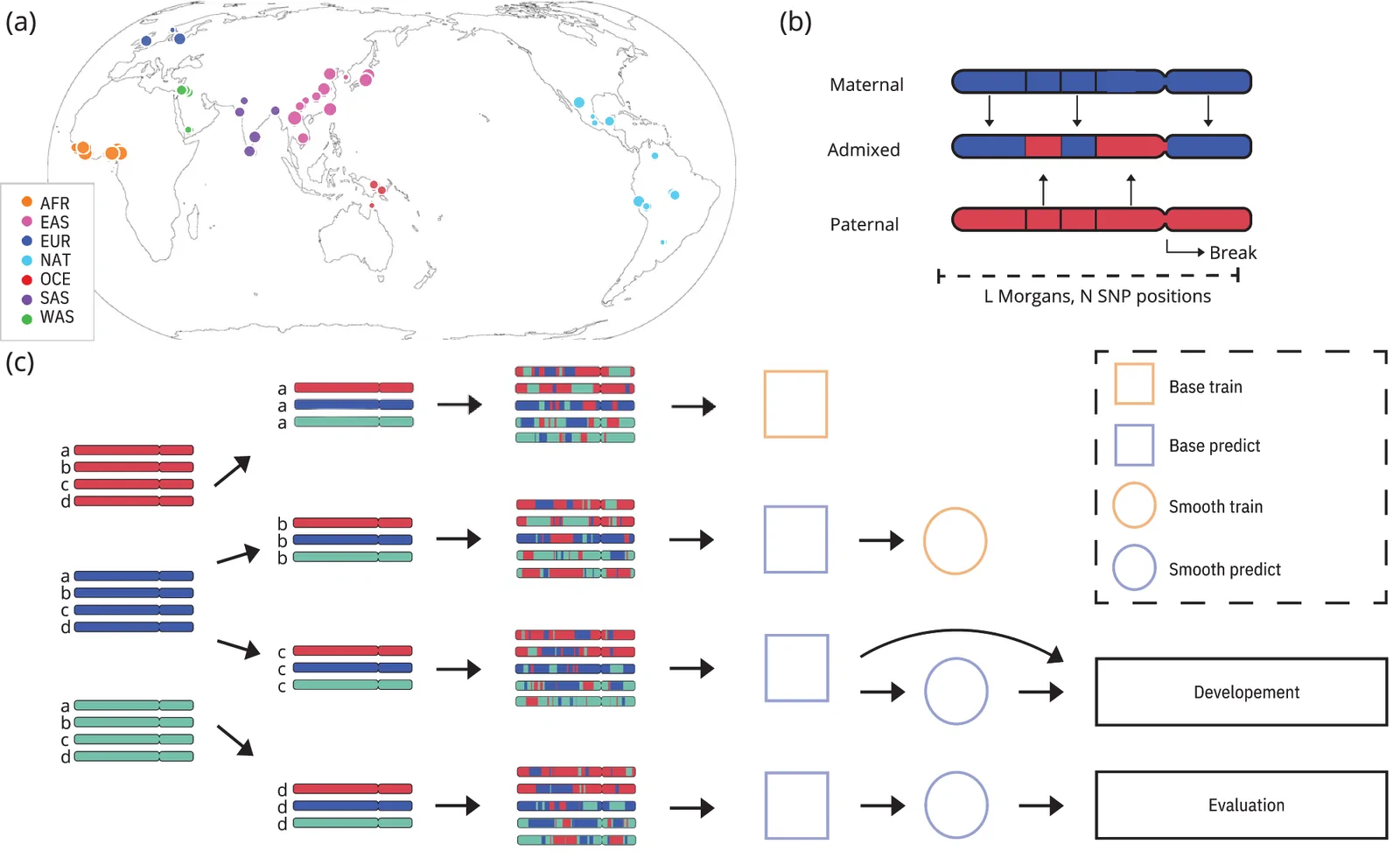
Origin and generation of human genome datasets. **a** Map of the distribution of single ancestry individuals used for dataset generation: African (AFR), East Asian (EAS), European (EUR), Indigenous American (NAT), Oceanian (OCE), South Asian (SAS), and West Asian (WAS). The base map was adapted from wikimedia^49^ and is used under the Creative Commons Attribution-Share Alike 4.0 International license. **b** Simulated chromosomal crossovers between the maternal and paternal chromosomes of a sample. The number of breakpoints is drawn from a Poisson distribution with a rate parameter dependent on the generations since admixture, and break loci are drawn uniformly along the chromosome. **c** Dataset generation via simulation and splitting. The labelled single-ancestry data is split into independent sets, and admixed individuals are simulated separately from each set to produce labelled admixed individuals. The base training data is used to train the base models, and the smooth training data is used to train the smoothing module independently. Similarly, the validation and test datasets are used to develop and evaluate the model, respectively.

For analysing and evaluating LAI models, genetic sequences from real admixed individuals cannot be used, as the ancestry switch points along their chromosomes cannot be known without sequencing a pedigree (trios). We therefore simulate admixed individuals from sequenced single-ancestry individuals (founders) from various populations (Fig. 7). Using these real individuals’ sequences, we simulate descendants of admixed ancestry using locus-specific recombination rate parameters from the human genetic map^15^. In brief, following Karavani et al.^46^, the number of switch points is modelled as a Poisson random variable parameterised by the chromosome length in Morgans. Recombination positions are simulated uniformly along the chromosome using the genetic map. The simulation process can be seen as a “select-and-stitch" procedure, where a simulated individual inherits each segment of their chromosome from a subset of founders, and the generation since admixture determines the statistical properties of segment lengths. This select-and-stitch process allows for much more lightweight computation than complete generation-wise recursive simulation of the matching explicitly, while also avoiding founder effects in admixed samples that arise from such recursion with small population sizes per generation.

Gnomix development was performed on a development (dev) dataset consisting of chromosome 22 of simulated admixed individuals drawn from an even distribution of founders from six populations, including African, East Asian, European, Indigenous American, South Asian, and West Asian. 70% of the founders were used for training, 12.5% for validation, and 17.5% for testing. All splits contained admixed individuals of generations 2, 4, 6, 8, 12, 16, 24, 32, 48, and 64. On the dev dataset, we compared various machine learning models for the base classifiers and smoother, where the hyperparameters of the individual models were selected by performing a grid search. We identified values for key Gnomix-specific parameters – window size, smoother window size, and context – that performed well across settings. While in theory these parameters could be application-specific, in practice we find our models to be largely robust to these particular parameters on a wide variety of ancestries.

For the benchmarking datasets, we split the founders randomly into training founders (90%) and testing founders (10%). The Latin American dataset included founders with European (EUR), Indigenous American (NAT), and African (AFR) ancestry. The 5-ancestry dataset used founders from the same populations as the Latin American dataset, but added South Asian (SAS) and East Asian (EAS). The 7-ancestry dataset further added Oceanian (OCE, referred to as Papuan in some publications) and West Asian (WAS, anchored in the Middle East). See Supplementary Table 2 for a more detailed breakdown. Each model had access to the training founders and was evaluated on a simulated admixed test set containing three times the number of samples as the testing founders. This corresponds to 182 (Latin American), 378 (5-ancestry), and 406 (7-ancestry) admixed diploid test individuals. The generations used for testing were 2, 4, 8, 12, 16, 20, 30, 40, 50, 60, 70, 80, 90, and 100, with individuals equally split across these generations.

The window size for Gnomix was one thousand SNPs for whole-genome data and thirty SNPs for the array data, and a context of the same size was used. The smoother size was 75 windows, and the model output was calibrated with isotonic regression. These parameters were selected based on performance on the validation set (further model hyperparameters are provided in the Supplementary Methods). The simulated admixed individuals used for training were from generations 0, 2, 4, 8, 16, 24, 32, 48, 64, 72, and 100, and each population sampling probability was weighted to provide class balance. The default settings were used for RFMix (v2), ELAI, and Loter. All benchmarking experiments were conducted using 32 CPUs with 256 GB of RAM. The computational cost of Gnomix scales approximately linearly with the number of windows; moderate datasets ( ~ 10,000 samples) can be run on modest resources, while larger datasets (~100,000 samples) benefit from increased parallelisation and memory.

### Phasing evaluation datasets

We simulate the phasing datasets of eighth-generation admixed individuals using Supplementary Algorithm 4 and then, after converting to genotypes, use Beagle 5.1 for phasing. The Latin American phasing dataset was simulated from the validation founders with an even distribution of Indigenous American, African, and European ancestry: eight founders from each population. 67 founders from each population were used for training. Gnofix used a smoother trained on this dataset with a window size of 0.2 cM. It considered swap collections containing a single swap within five or fewer windows away from the centre. Lastly, we iterated through scopes where the base module probabilities were discontinuous. Since RFMix version 2 cannot perform phasing error correction, we used RFMix version 1 with the default parameters and a window size also set to 0.2 cM.

### Canid datasets

For training Gnomix on canid whole genomes, phased first using Beagle^36^, we used the following groupings as founders (sample numbers for each type in brackets): Coyote [3], Wolf (Grey Wolf [45] and Iberian Wolf [1]), Basenji [4], Tibetan Mastiff [10], Border Collie [15], Bull Terrier [9], German Shepherd [15], Greyhound [9], Hound (Afghan Hound [3] and Saluki [3]), Retriever (Labrador [24] and Golden [20]), Arctic (Alaskan Husky [2], Siberian Husky [4], Greenland Dog [1], and Alaskan Malamute [3]), and East Asian (Shiba Inu [2], Chinese Shar-Pei [2], Jindo [1], New Guinea Singing Dog [4], Chongqing Dog [1], Xiasi Dog [1], and Chow Chow [4]). These groups were chosen based on the neighbour-joining tree in Plassais et al. in order to ensure broad coverage from each clade of the tree^28^. With these groupings, we trained a Gnomix model for each of the 38 canid autosomes with the following configuration: logistic regression base, XGBoost smoother, and a 0.2 cM window size. We obtained an overall validation accuracy of 90.63%. This lower accuracy can be attributed to the single-digit numbers of whole-genome training references available for many of the groupings (see above). Our >90% accuracy despite such a limited number of references illustrates the ability of Gnomix to perform even when labelled training data is scarce, a situation often encountered.

To produce the tree in Fig. 6, we used neighbour-joining^47^ with a pairwise distance matrix constructed using the average number of pairwise differences (*π*) between various breeds as a dissimilarity measure^48^. The standard error for the number of generations since admixture was computed by performing one thousand bootstrap replicates and refitting the exponential each time using maximum likelihood.

### Reporting summary

Further information on research design is available in the Nature Portfolio Reporting Summary linked to this article.

## Supplementary information

Supplementary Information

Reporting Summary

Transparent Peer Review file

## Data Availability

The human genome datasets used in this study for admixture simulations and reference panels are publicly available through the Human Genome Diversity Project (HGDP) at https://www.internationalgenome.org/data-portal/data-collection/hgdp, the Simons Genome Diversity Project (SGDP) at https://www.simonsfoundation.org/simons-genome-diversity-project/, and the 1000 Genomes Project at https://www.internationalgenome.org. A merge of the 1000 Genomes Project with HGDP can be found at https://gnomad.broadinstitute.org/downloads#v3-hgdp-1kg. The canid genome dataset used in this study is available from the NCBI BioProject database under accession code PRJNA448733 at https://www.ncbi.nlm.nih.gov/bioproject/PRJNA448733. All data supporting our findings are available in the article, its Supplementary Information files, or the repositories listed above.

## Code availability

The code for Gnomix is available at https://github.com/AI-sandbox/gnomix, along with a user guide, documentation, pre-trained models, and instructions for training on reference datasets of choice.
