# Supplementary material for: Scalable high resolution ancestry deconvolution for genomic data
Source: Nat Commun. 2026 Jul 25;17:9070. doi: 10.1038/s41467-026-75391-0 (PMC13518915; doi:10.1038/s41467-026-75391-0)
Supplement: Supplementary file 1 [file 41467_2026_75391_MOESM1_ESM.pdf]

Supplementary Information

Scalable High Resolution Ancestry Deconvolution for Genomic Data

Helgi Hilmarsson[1,†], Arvind S. Kumar[1,†], Míriam Barrabés[2,†], Richa Rastogi[3], Carlos D. Bustamante[2,4], Daniel Mas Montserrat[2], and Alexander G. Ioannidis[1,2,5,*]

[1]Stanford University, Institute for Computational and Mathematical Engineering, Stanford, CA, 94305, USA
[2]Stanford University, Department of Biomedical Data Science, Stanford, CA, 94305, USA
[3]Cornell University, Department of Computer Science, New York, 10044, USA
[4]University of Oklahoma, College of Medicine, Norman, OK, 73019, USA
[5]Genomics Institute, University of California Santa Cruz, Santa Cruz, CA, 95060, USA
[†]These authors contributed equally to this work
[*]Corresponding author: ioannidis@stanford.edu

Supplementary Methods

Dataset composition

We generated four simulated datasets for method development and benchmarking: Latin American, 5-ancestry, 7-ancestry, and development (dev). The Latin American dataset contains African (AFR), European (EUR), and Indigenous American (NAT) ancestry groups. The 5-ancestry dataset additionally includes South Asian (SAS) and East Asian (EAS) ancestries, whereas the 7-ancestry dataset further incorporates Oceanian (OCE) and West Asian (WAS) ancestries. The dev dataset contains AFR, EUR, NAT, SAS, EAS, and WAS ancestries and was used exclusively for method development and hyperparameter optimisation. The Latin American, 5-ancestry, and 7-ancestry datasets were used as benchmark datasets. The dev dataset was reserved for model development and parameter tuning and was generated using genomic sequences from a chromosome distinct from those used in the benchmark datasets.

Details on the number of single-ancestry founder individuals used for each simulated dataset are summarised in Supplementary Table 1, including both the ancestry-level counts per dataset and the corresponding population-level breakdown of reference samples. The corresponding population-level composition of the founder panels is provided in Supplementary Table 2.

Supplementary Table 1. Simulated datasets and founder counts by ancestry. Chromosome and founder counts used to generate the Latin American, 5-ancestry, 7-ancestry, and development (dev) simulated admixed datasets. Founder counts are reported by ancestry group: African (AFR), European (EUR), Indigenous American (NAT), South Asian (SAS), East Asian (EAS), Oceanian (OCE), and West Asian (WAS). Size reports the total number of simulated admixed individuals, including training and testing samples. Total reports the total number of single-ancestry founder samples across ancestry groups.

			Number of Founders							
Dataset	Size	chr	AFR	EUR	NAT	SAS	EAS	OCE	WAS	Total
Latin American	2421	20	362	146	71	-	-	-	-	579
5-ancestry	5086	20	362	146	71	162	469	-	-	1210
7-ancestry	5420	20	362	146	71	162	469	17	63	1290
Dev	1614	22	112	112	112	112	112	-	112	672

Supplementary Table 2. Population-level breakdown of founder individuals. Population-level composition of the single-ancestry founder reference panels used to generate the simulated admixed datasets. Counts are reported within each ancestry group: African (AFR), East Asian (EAS), European (EUR), Indigenous American (NAT), Oceanian (OCE), South Asian (SAS), and West Asian (WAS).

Superpopulation	Population	Size	Superpopulation	Population	Size
AFR	Mandenka	11	NAT	Mexican-American	1
	Yoruba	121		Surui	8
	Mende	79		Chane	1
	Gambian Mandinka	60		Karitiana	11
	Esan	91		Piapoco	2
EAS	Southern Han Chinese	101		Maya	9
	Dai Chinese	82		Mixe	3
	Kinh Vietnamese	26		Quechua	2
	Han Chinese	107		Pima	13
	Japanese	110		Mixtec	1
	Yi	3	OCE	Australian	2
	Miao	9		Papuan Highlands	9
	Dai	8		Papuan Sepik	6
	She	10	SAS	Telugu	69
	Naxi	4		Bengali	2
	Tujia	9		Punjabi	6
EUR	Norwegian	1		Sri Lankan	70
	USA-European (CEPH)	63		Gujarati	15
	British	82	WAS	Palestinian	21
NAT	Colombian	3		Bedouin	19
	Zapotec	2		Druze	22
	Peruvian	15		Yemenite Jewish	1

Model hyperparameters

Hyperparameters for all models were selected via grid search on the development dataset:

- Base models. Logistic regression uses L2 regularisation with $C = 3$, the liblinear solver, and a maximum of 1000 iterations. The XGBoost base model uses 20 trees, maximum depth 4, learning rate 0.1, $\lambda = 1$, and $\alpha = 0$. Support vector machines with CovRSK use an SVM classifier with probability estimates enabled, where CovRSK is used as the kernel function.

- Smoothing models. The XGBoost smoother uses 100 trees, maximum depth 4, learning rate 0.1, $\lambda = 1$, and $\alpha = 0$, with a multinomial soft-probability objective and mlogloss evaluation metric. The CRF smoother uses a linear-chain CRF with the L-BFGS optimiser, a maximum of 10000 iterations, and all possible transitions and states enabled. The CNN smoother consists of a 1D convolutional layer with reflection padding and dropout with rate 0.5, and is trained using the Adam optimiser with learning rate 0.001.

Algorithms for string kernel computation

In this section, we list algorithms for computing the standard string kernel and its covariance-reducing extension. Supplementary Algorithm 1 provides a detailed, lower-level description of the string kernel computation with triangular numbers. Supplementary Algorithm 2 gives the dynamic programming (DP) version, and Supplementary Algorithm 3 gives the DP version customised for CovRSK.

Supplementary Algorithm 1: Detailed algorithm for string kernel with triangular numbers

---

Input: Feature vectors x, $x' \in \mathbb{R}^M$
`/* Find all contiguous matches and their lengths */`
$\Lambda \leftarrow \{\}$, $\lambda \leftarrow 0$
for $i \in \{1, 2, \ldots, M\}$ **do**
  if $x_i == x'_i$ **then**
    $\lambda \leftarrow \lambda + 1$
  else
    $\Lambda \leftarrow \Lambda \cup \{\lambda\}$
    $\lambda \leftarrow 0$
  end
end
`/* Sum up the triangular numbers of the matches */`
$K \leftarrow \sum_{\lambda \in \Lambda} T(\lambda)$
Result: String kernel similarity, $K(x, x')$

Supplementary Algorithm 2: String kernel with triangular numbers and DP

Input: Feature vectors x, $x' \in \mathbb{R}^M$
$K \leftarrow 0, \lambda \leftarrow 0$
for $i \in \{1, 2, \ldots, M\}$ **do**
  if $x_i == x'_i$ **then**
    $\lambda \leftarrow \lambda + 1$
    $K \leftarrow K + \lambda$
  else
    $\lambda \leftarrow 0$
  end
end
Result: String kernel similarity, $K(x, x')$

Supplementary Algorithm 3: DP version of CovRSK

Input: Feature vectors x, $x' \in \mathbb{R}^M$ and k-mer lengths, k
$\not\Bbbk_k \leftarrow$ one_hot_encode(k)
$\varkappa \leftarrow 0, \lambda \leftarrow 0, a \leftarrow 0$
for $i \in \{1, 2, \ldots, M\}$ **do**
  if $x_i == x'_i$ **then**
    $\lambda \leftarrow \lambda + 1$
    $a \leftarrow a + \not\Bbbk_k[\lambda]$
    $\varkappa \leftarrow \varkappa + a$
  else
    $\lambda \leftarrow 0$, $a \leftarrow 0$
  end
end
Result: CovRSK similarity, $\varkappa(x, x')$

Phasing error correction dataset generation

We use a dataset of simulated admixed individuals with known local ancestry labels to create a dataset containing phasing errors. Since each segment from the single-ancestry dataset is correctly phased up to its ancestry, we can create an instance of a correctly ancestry phased individual by collapsing the simulated admixed individuals created from them to unphased genotypes and then applying standard phasing software (here Beagle 5.1). This procedure is described in Supplementary Algorithm 4 and produces the evaluation dataset that we refer to as the Latin American phasing dataset. The simulated individuals in this dataset model modern Latin American individuals with eight generations since the onset of admixture among Indigenous American, African, and European ancestries. We also apply the same procedure to construct the continental phasing dataset; the composition of the single-ancestry founder individuals used for this dataset is reported in Supplementary Table 3.

Supplementary Algorithm 4: Simulating data containing phasing errors

Input:

- Dataset S of phased haplotypes from single ancestry individuals
- Standard phasing algorithm $C : \tilde{x} \mapsto x$

$S_1, S_2 \leftarrow \text{split}(S)$

Simulate haplotypes from admixed individuals, x_2, from S_2

Pairwise shuffle SNPs of x_2 to remove prior phasing: $\tilde{z}_2 \leftarrow P(x_2)$

Phase $\tilde{z}_2$ with S_1 as reference panel: $z_2 \leftarrow C(\tilde{z}_2|S_1)$

Result:

- Haplotypes of admixed individuals containing phasing errors, z_2
- Corresponding haplotypes with correct phasing, x_2

Supplementary Table 3. Single-ancestry founder composition for the phasing error correction dataset. Counts of single-ancestry founder individuals used to generate the phasing error correction dataset. Founder counts are reported for African (AFR), African Hunter-Gatherer (AHG), East Asian (EAS), European (EUR), Indigenous American (NAT), Oceanian (OCE), South Asian (SAS), and West Asian (WAS) ancestry groups.

	AFR	AHG	EAS	EUR	NAT	OCE	SAS	WAS
Continental phasing	363	18	366	119	53	15	135	56

Supplementary Discussion

More on smoother data

The training set is split into two parts, one for training the base model and one for the smoother, to avoid distributional shift. Supplementary Figs. 1 and 2 show how the estimated probabilities differ between the training data and the independent validation dataset. These results are shown for the development dataset using XGBoost as the base model. It is clear that the estimated probabilities on the training data are sharper. That is, these probabilities tend to have much higher confidence (maximum predicted probability).

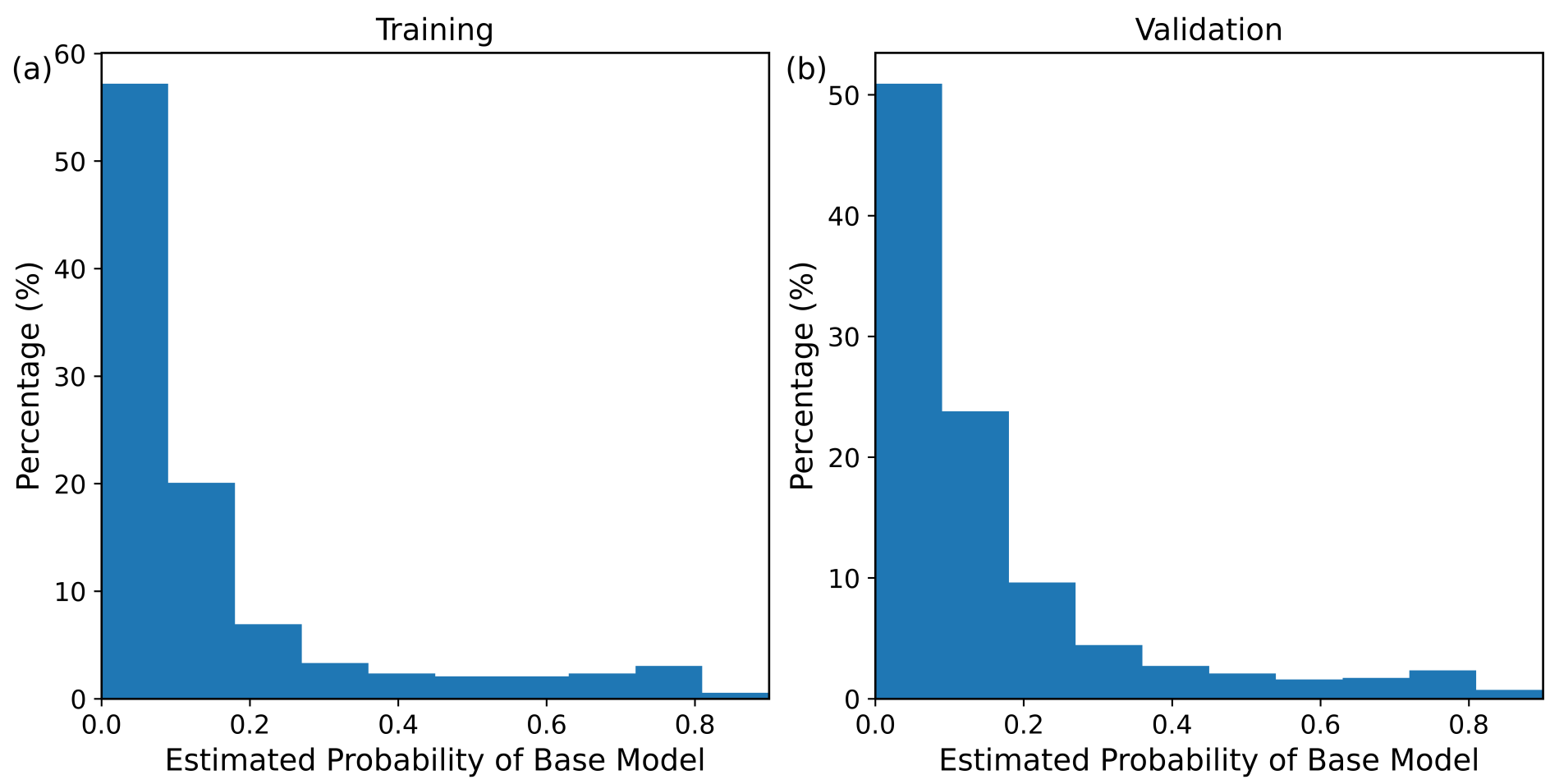

Supplementary Fig. 1. Distribution of maximum predicted probabilities for training and validation individuals. **a** Distribution of maximum ancestry probabilities predicted by the base model for training individuals. **b** Distribution of maximum ancestry probabilities predicted by the same model for independent validation individuals.

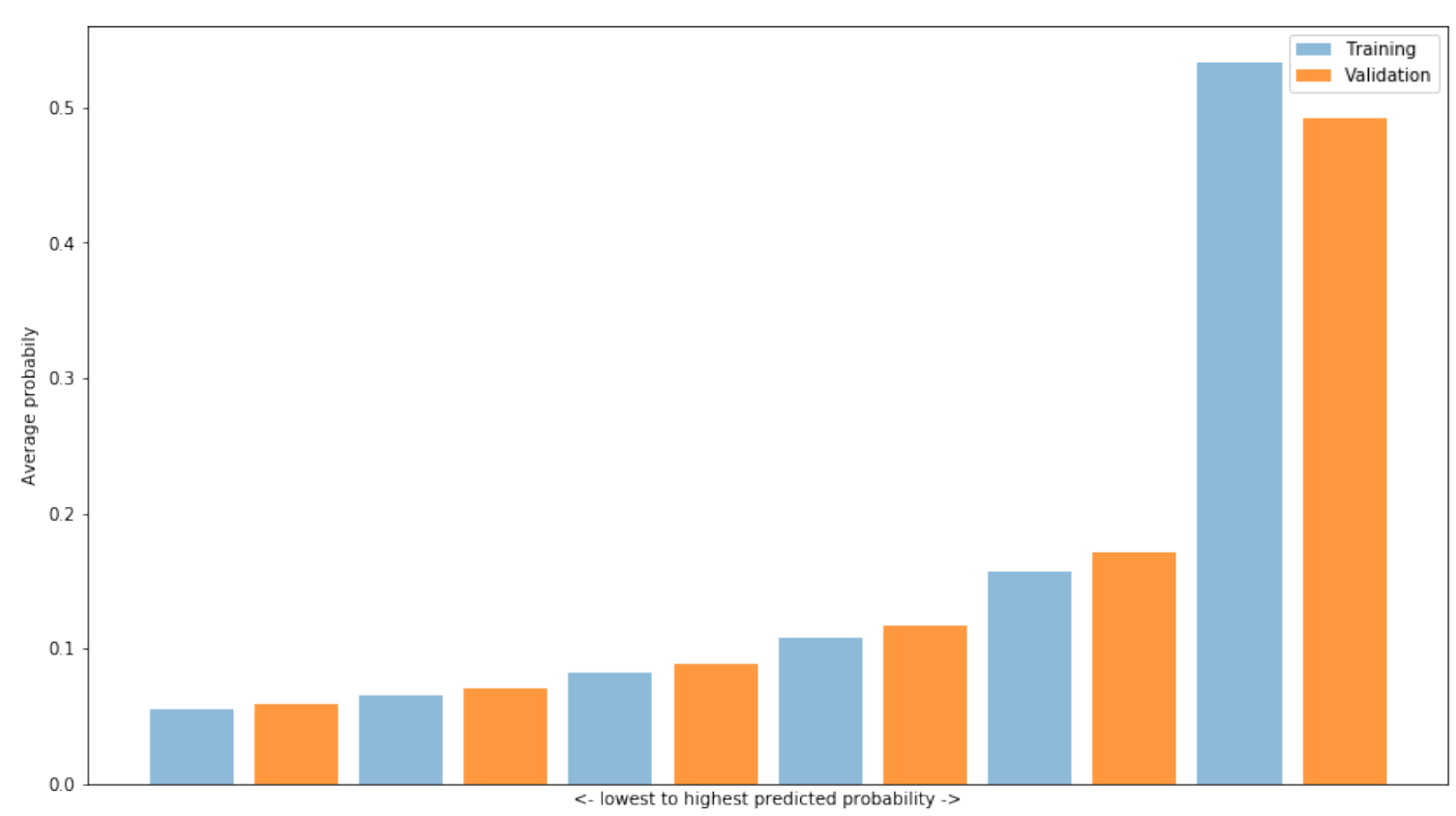

Supplementary Fig. 2. Average ranked ancestry probabilities for training and validation individuals. Bars show the average ancestry probabilities ranked from lowest to highest for training (blue) and validation (orange) individuals.

Furthermore, we argue that the smoothing module is not adversely affected by the reduction in training data, as it only requires a small fraction of the available samples to perform well, as shown in Supplementary Fig. 3. The dataset is the same as above, with XGBoost used as the smoothing module.

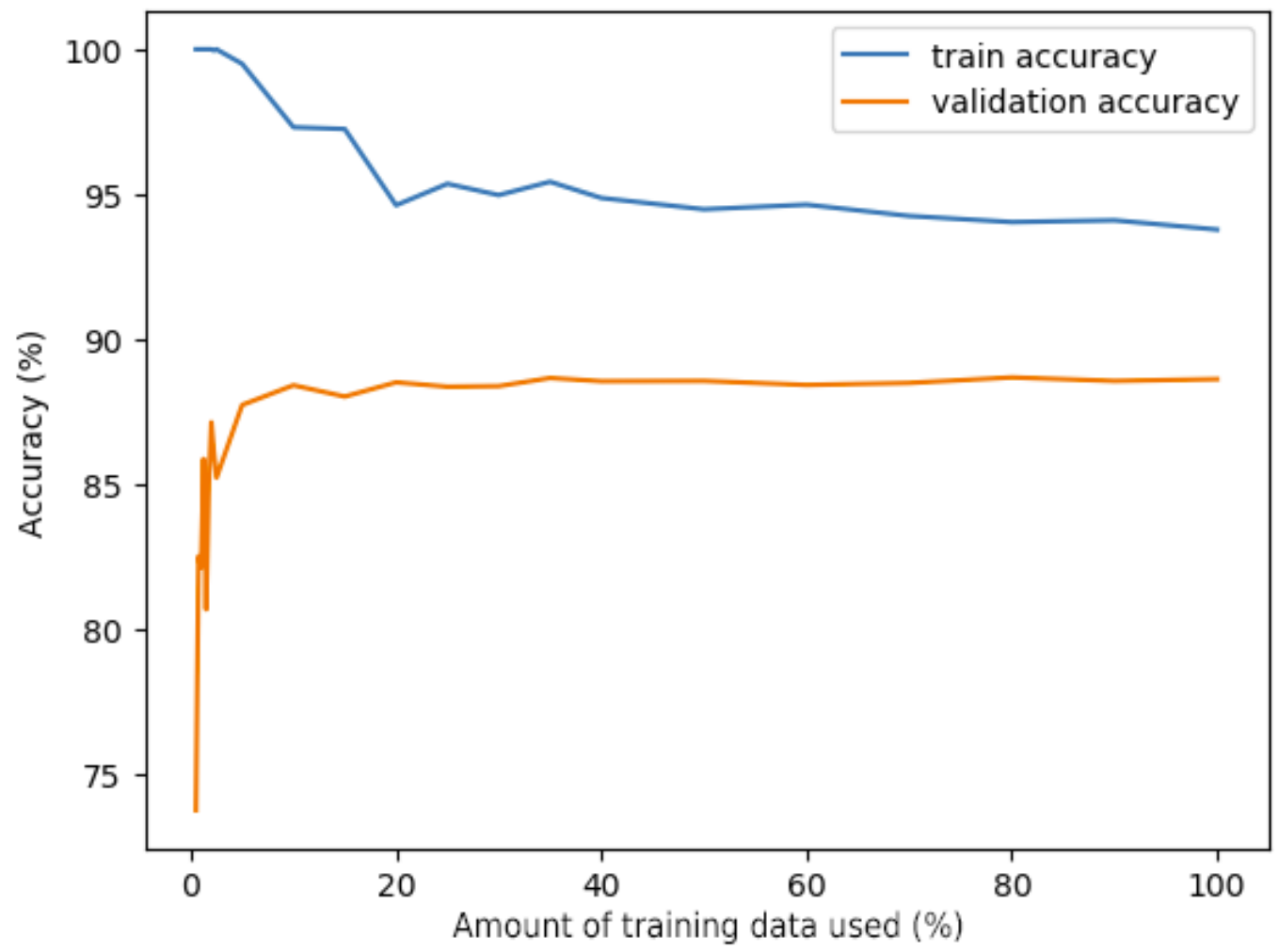

Supplementary Fig. 3. Smoother training saturation. Training (blue) and validation (orange) accuracy of the XGBoost smoothing module as a function of the proportion of available training data used for smoother training. Validation accuracy improves rapidly with the first few percent of samples and then approaches a plateau, indicating that the smoother is highly data-efficient and requires only a relatively small subset of the available data to achieve near-optimal performance.

Empirical studies of calibration

For quantitative evaluation, we compare log-loss and calibration error [1], and find that both improve as a result of calibration. A comparison of the probabilities before and after calibration can be seen in the reliability plots in Supplementary Fig. 4. The figure shows the estimated probabilities and their respective true probabilities for two ancestries (West Asian and Indigenous American). The mismatch between estimated and true probabilities is larger before calibration (dashed lines) than after (solid lines). For this dataset, the calibration error was 0.0257 before calibration and 0.0145 after. The log-loss decreased from 0.3349 to 0.3249.

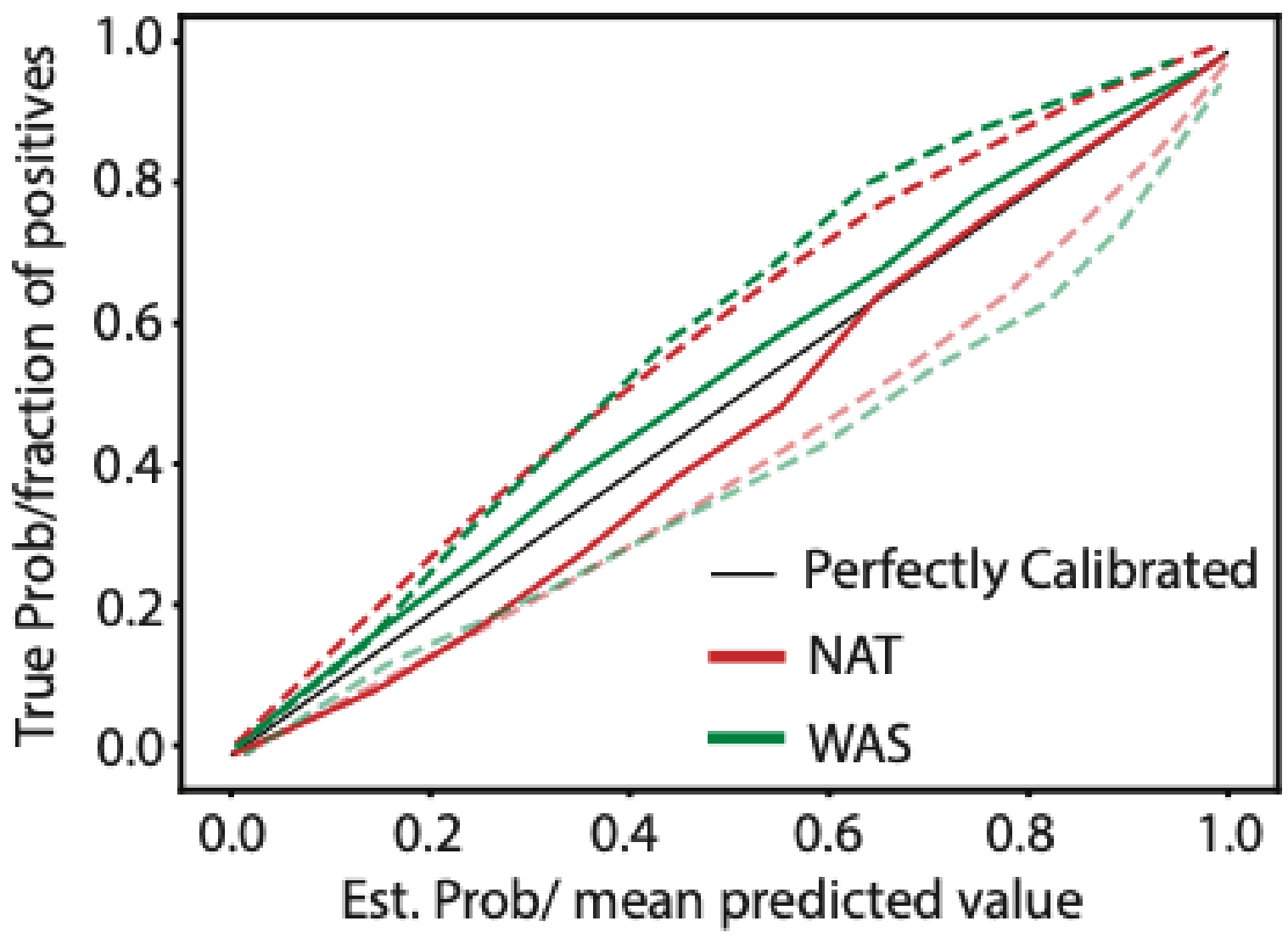

Supplementary Fig. 4. Reliability plots before and after calibration. Reliability curves comparing estimated mean predicted probabilities with the corresponding empirical fraction of positives for Indigenous American (NAT, red) and West Asian (WAS, green) ancestry predictions. Dashed lines show uncalibrated probabilities, and solid lines show probabilities after calibration. The black diagonal indicates perfect calibration; curves closer to this line indicate better calibration. The uncalibrated curves are mirrored across the diagonal to visually illustrate the centrality of the calibrated ones.

Benchmarking results

We benchmark Gnomix configurations and competing methods on three human datasets (Latin American, 5-ancestry, and 7-ancestry) using both whole genome and genotyping array data (UK Biobank Axiom SNPs). Results are reported in Supplementary Tables 4–6 for whole genome data and Supplementary Tables 7–9 for array data. Reported metrics include classification accuracy, log loss, base-model accuracy, training time, and inference time. Accuracy and log loss values are averaged across samples from all simulated generations. Note that ELAI and Loter are only benchmarked with the 7-ancestry dataset due to their extremely high computational requirements.

Supplementary Table 4. 7-ancestry (whole genome). Classification accuracy, log loss, base-model accuracy, training time, and inference time. Boldface indicates the best value in each column. Dashes (–) in inference times indicate methods that cannot perform inference using previously trained models.

model	accuracy (%)	log loss	base accuracy (%)	training time (s)	inference time (s)
CovRSK-crf	93.10	0.21	**88.17**	11392	4731
CovRSK-xgb	**93.35**	**0.19**	**88.17**	13200	4727
CovRSK-cnn	93.01	0.22	**88.17**	11379	4722
logreg-crf	93.13	0.20	87.52	635	30
logreg-xgb	93.28	0.20	87.52	2436	26
logreg-cnn	93.07	0.21	87.52	655	**21**
rfmix	88.18	-	-	5518	-
elai	92.34	-	-	334003	-
FLARE	92.30	-	-	320	73
Orchestra	80.71	-	-	149574	3481
RecombMix	91.35	-	-	**146**	61

Supplementary Table 5. 5-ancestry (whole genome). Classification accuracy, log loss, base-model accuracy, training time, and inference time. Boldface indicates the best value in each column. Dashes (–) in inference times indicate methods that cannot perform inference using previously trained models.

model	accuracy (%)	log loss	base accuracy (%)	training time (s)	inference time (s)
CovRSK-crf	95.16	0.14	**91.50**	11519	5004
CovRSK-xgb	**95.40**	0.13	**91.50**	12608	5001
CovRSK-cnn	95.07	0.14	**91.50**	11549	4997
logreg-crf	95.19	0.14	91.40	433	23
logreg-xgb	**95.40**	**0.12**	91.40	1344	20
logreg-cnn	95.09	0.14	91.40	465	**17**
rfmix	90.76	-	-	4261	-
FLARE	94.92	-	-	222	57
Orchestra	87.47	-	-	160898	3436
RecombMix	94.76	-	-	**91**	48

Supplementary Table 6. Latin American (whole genome). Classification accuracy, log loss, base-model accuracy, training time, and inference time. Boldface indicates the best value in each column. Dashes (–) in inference times indicate methods that cannot perform inference using previously trained models.

model	accuracy (%)	log loss	base accuracy (%)	training time (s)	inference time (s)
CovRSK-crf	97.63	0.07	97.52	3241	1933
CovRSK-xgb	97.70	**0.06**	97.52	3399	1932
CovRSK-cnn	97.29	0.08	97.52	3252	1931
logreg-crf	97.69	**0.06**	**97.55**	76	14
logreg-xgb	97.79	**0.06**	**97.55**	230	13
logreg-cnn	97.29	0.08	**97.55**	86	**12**
rfmix	95.94	-	-	1594	-
FLARE	97.74	-	-	**50**	26
Orchestra	90.35	-	-	39642	1526
RecombMix	**97.92**	-	-	67	22

Supplementary Table 7. 7-ancestry (array genotype). Classification accuracy, log loss, base-model accuracy, training time, and inference time. Boldface indicates the best value in each column. Dashes (–) in inference times indicate methods that cannot perform inference using previously trained models.

model	accuracy (%)	log loss	base accuracy (%)	training time (s)	inference time (s)
CovRSK-crf	89.20	**0.33**	**77.97**	324	97
CovRSK-xgb	**89.35**	**0.33**	**77.97**	2470	93
CovRSK-cnn	89.07	0.35	**77.97**	324	87
logreg-crf	86.57	0.40	67.90	1402	11
logreg-xgb	86.01	0.42	67.90	3792	7
logreg-cnn	86.31	0.41	67.90	1555	**1**
rfmix	84.81	-	-	241	-
elai	88.32	-	-	37219	-
loter	83.86	-	-	8840	-
FLARE	87.03	-	-	21	7
Orchestra	77.76	-	-	18205	410
RecombMix	70.89	-	-	**2**	2

Supplementary Table 8. 5-ancestry (array genotype). Classification accuracy, log loss, base-model accuracy, training time, and inference time. Boldface indicates the best value in each column. Dashes (–) in inference times indicate methods that cannot perform inference using previously trained models.

model	accuracy (%)	log loss	base accuracy (%)	training time (s)	inference time (s)
CovRSK-crf	91.85	0.24	**81.61**	241	86
CovRSK-xgb	**92.06**	**0.23**	**81.61**	1307	83
CovRSK-cnn	91.72	0.24	**81.61**	272	79
logreg-crf	90.08	0.29	74.46	37	15
logreg-xgb	89.99	0.29	74.46	887	13
logreg-cnn	90.00	0.29	74.46	70	9
rfmix	90.28	-	-	196	-
FLARE	90.24	-	-	17	7
Orchestra	83.17	-	-	14870	266
RecombMix	75.57	-	-	**4**	**1**

Supplementary Table 9. Latin American (array genotype). Classification accuracy, log loss, base-model accuracy, training time, and inference time. Boldface indicates the best value in each column. Dashes (–) in inference times indicate methods that cannot perform inference using previously trained models.

model	accuracy (%)	log loss	base accuracy (%)	training time (s)	inference time (s)
CovRSK-crf	96.55	0.10	**93.86**	67	32
CovRSK-xgb	**96.67**	**0.09**	**93.86**	233	31
CovRSK-cnn	96.36	0.11	**93.86**	79	29
logreg-crf	96.23	0.11	91.68	12	8
logreg-xgb	96.30	0.10	91.68	168	7
logreg-cnn	95.90	0.12	91.68	23	6
rfmix	95.58	-	-	82	-
FLARE	95.69	-	-	7	4
Orchestra	90.81	-	-	9128	158
RecombMix	90.88	-	-	**1**	**1**

Error analysis

Accuracy and confidence versus distance from ancestry switch points

While model predictions are important, it is also necessary to understand signs of uncertainty in the predicted estimates. In Supplementary Fig. 5, we show how the model is more likely to make mistakes near ancestry switch points (break points). This explains in part the decay of accuracy as generation time since admixture increases. With higher generation time, we have more frequent breakpoints leading to a more difficult problem.

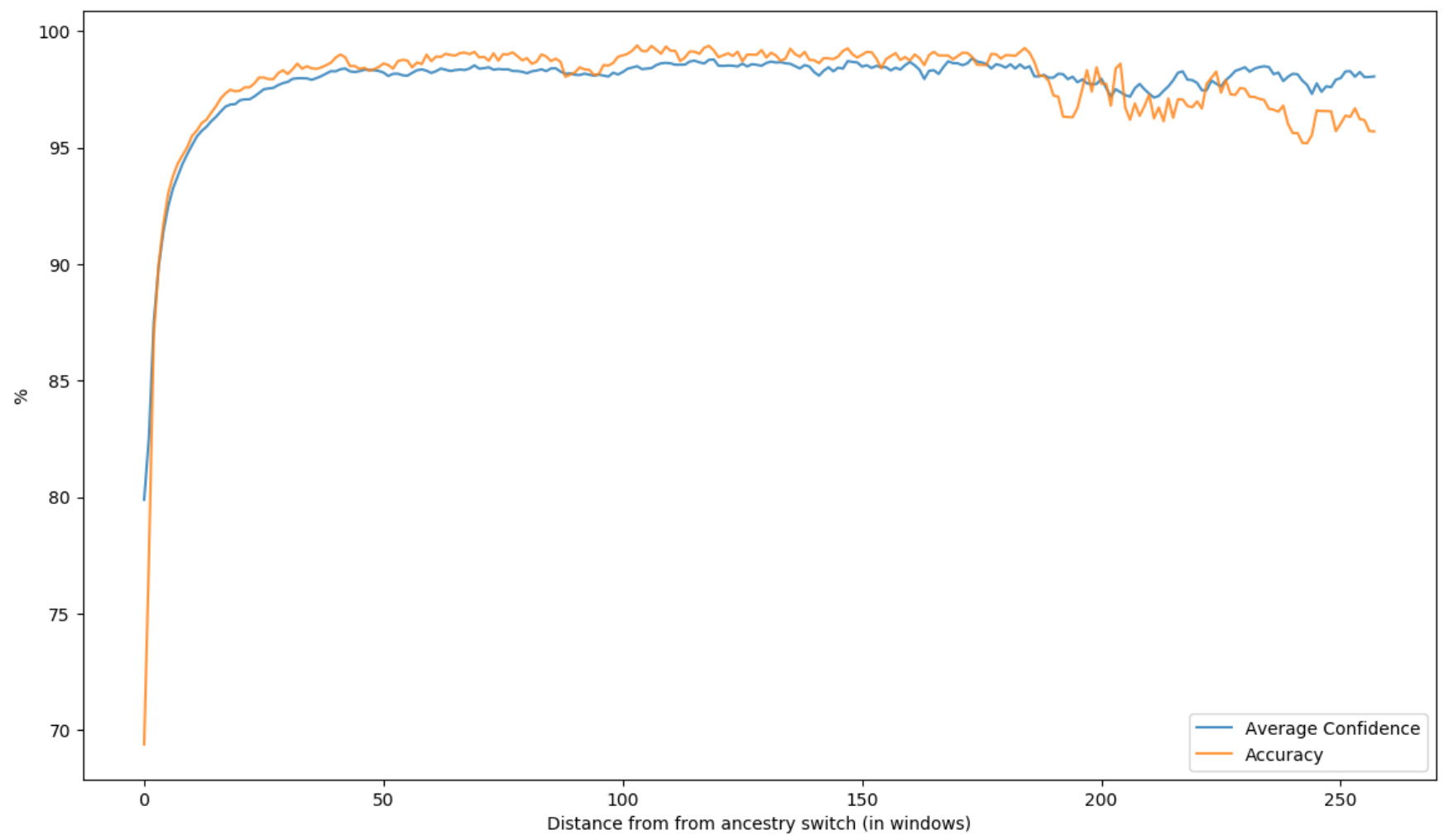

Supplementary Fig. 5. Accuracy and confidence as a function of distance from ancestry switch points. Validation accuracy and average prediction confidence as a function of distance from the nearest ancestry switch point, measured in genomic windows. Accuracy and confidence are lowest near switch points, where local ancestry transitions make classification more uncertain, and increase rapidly with distance from the breakpoint. This indicates that prediction errors are concentrated near ancestry transitions, partly explaining why inference becomes more difficult as the number of ancestry breakpoints increases.

Confusion matrices for Gnomix and RFMix

In Supplementary Fig. 6 we plot confusion matrices for Gnomix and RFMix on generation 64 validation data in the dev dataset, using a shared colour scale to facilitate direct comparison. It is clear that some population pairs present a harder classification task than others, for instance, the neighbouring European (EUR) and West Asian (WAS) ancestries. The two methods make similar types of errors, but Gnomix exhibits fewer misclassifications overall.

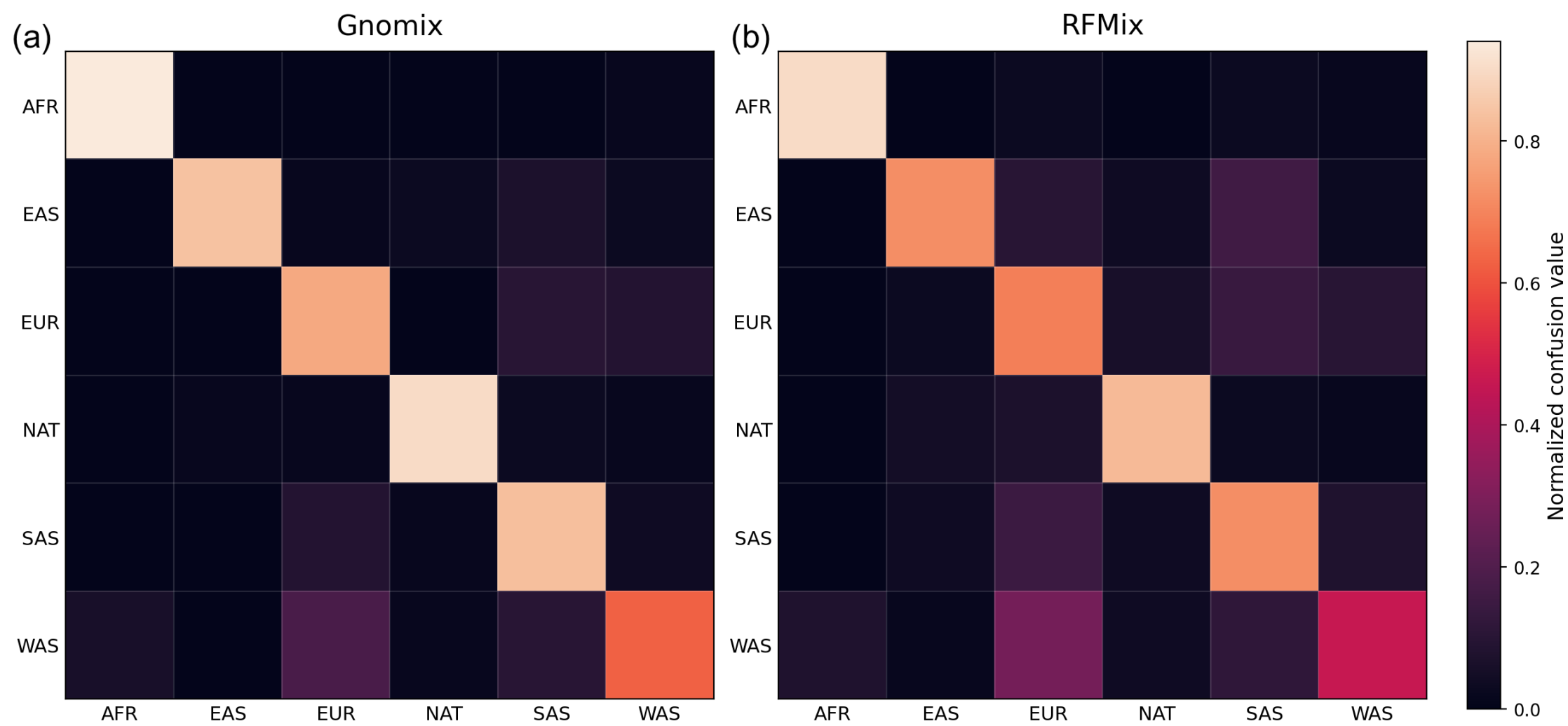

Supplementary Fig. 6. Confusion matrices for Gnomix and RFMix on generation 64 validation data. Normalised confusion matrices for **a** Gnomix and **b** RFMix on generation 64 validation samples from the development dataset, shown with a shared colour scale for direct comparison. Ancestries include African (AFR), East Asian (EAS), European (EUR), Indigenous American (NAT), South Asian (SAS), and West Asian (WAS). Both methods show similar patterns of confusion across ancestries, but Gnomix exhibits fewer off-diagonal misclassifications overall.

Runtime analysis of phasing error correction

RFMix's phasing error correction algorithm is hard to time separately, since its phase correction and ancestry estimation are intertwined. A lower bound on the correction time can be estimated by measuring RFMix's run-time for performing LAI without phase correction and then subtracting this from its run-time for performing combined LAI and phase correction. When the number of ancestries becomes large (e.g., more than five), the run-time is hard to exhaustively measure for many individuals. Comparative run-times for Beagle, RFMix, and Gnofix are summarised in Supplementary Table 10. As we can see in Supplementary Fig. 7, the lower bound on the phase correction run-time for RFMix increases superlinearly with the number of ancestries. Indeed, at just over 10 ancestries, the phase correction in RFMix becomes infeasible for any sizeable number of query individuals.

Supplementary Table 10. Run-times for phasing and phasing error correction. Run-times are reported in seconds for Beagle 5.1 phasing and for phase-correction methods on the Latin American and continental datasets. RFMix run-times correspond to a lower-bound estimate of phase-correction time, computed by subtracting the run-time for local ancestry inference without phase correction from the run-time for combined local ancestry inference and phase correction. Values in parentheses report relative speed-up compared to RFMix. Boldface indicates the fastest phase-correction method for each dataset.

	Latin American	continental
Beagle 5.1	316	800
RFMix	2,252 s (1×)	172,800 s (1×)
Gnofix	**7 s** (321.7×)	**98 s** (1,763.3×)

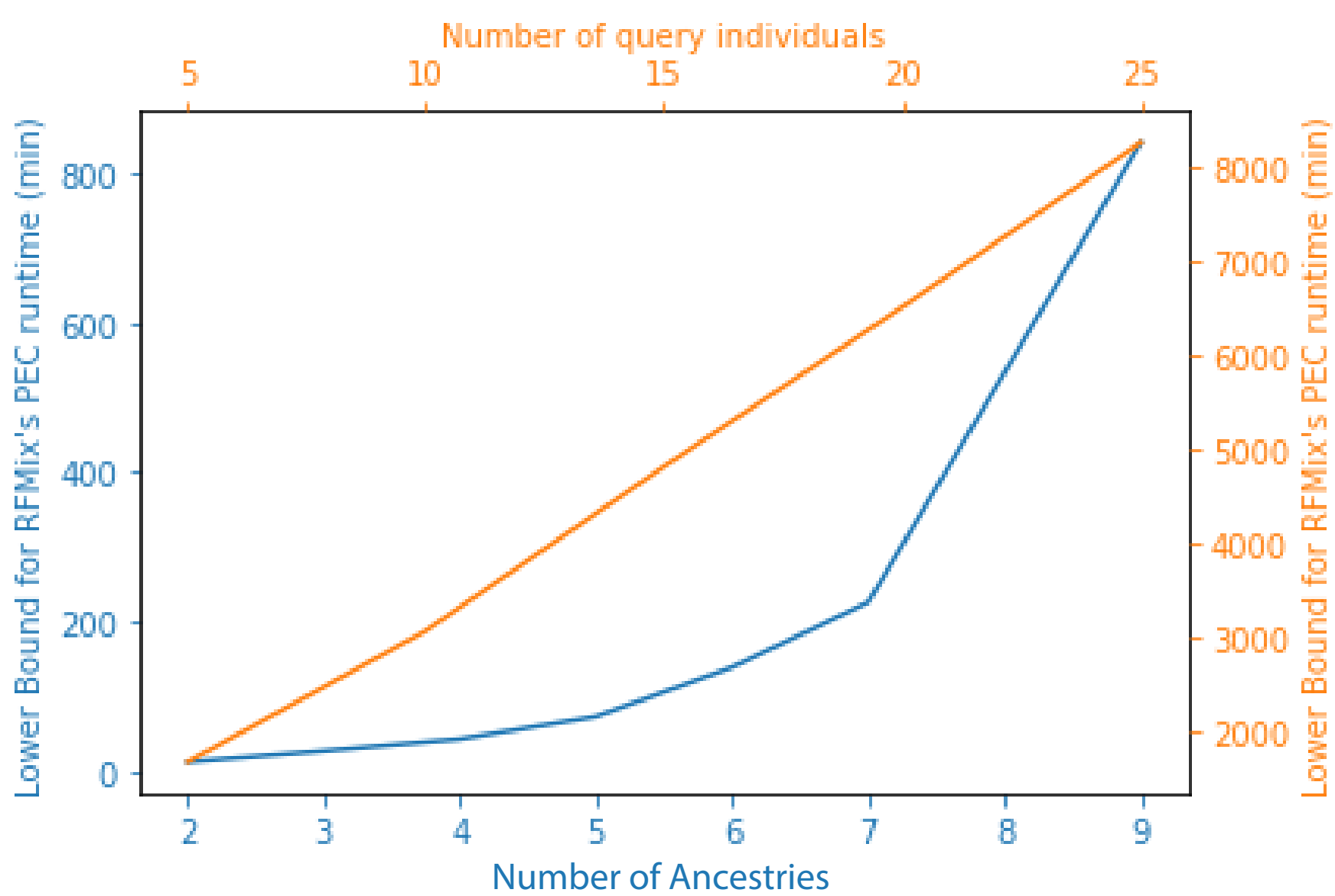

Supplementary Fig. 7. RFMix phase-correction run-time scaling. Lower-bound estimates of RFMix phase-correction run-time as a function of the number of ancestries and query individuals. The blue curve shows run-time as the number of ancestries increases, with reference panel size fixed at 240 and one query individual. The orange curve shows run-time as the number of query individuals increases, with reference panel size fixed at 350 and the number of ancestries fixed at three. Run-time increases approximately linearly with the number of query individuals but superlinearly with the number of ancestries.

String kernel analysis

Efficient computation of the string kernel

From the canonical string kernel equation in the main text (Equation (1)), which involves an inner product between two sequence vectors, it is evident that a naive computation scales quadratically with sequence length. However, unless the two inputs are a perfect match, it is unnecessary to examine all subsequences. If a subsequence of length k starting at a given position does not match, then no subsequence of length $k' > k$ starting at the same position can match either. Thus, a dynamic programming (DP) algorithm that extends matches only after shorter matches have been confirmed is appropriate. This naturally leads to a recursive structure, where checks for longer matches are performed only after all shorter matches at the same positions have been identified. Without care, however, such an approach may still result in quadratic complexity. In the worst case, when the two sequences match exactly, one must compute

$$M + (M-1) + \cdots + 1 = \frac{M(M+1)}{2} \in O(M^2)$$

additions, one for each matching k-mer.

A more efficient approach follows from the realisation that each contiguous matching subsequence μ contributes independently to the total similarity,

$$K(x, x') = \sum_{\mu} T(\lambda_\mu),$$

where λ_μ is the length of match μ, and $T(\lambda)$ is the number of k-mer matches contained within a contiguous match of length λ. The number of k-mer matches within such a segment is the triangular number of its length,

$$T(\lambda) = \sum_{k=1}^{\lambda} k = \frac{\lambda(\lambda+1)}{2}.$$

This observation yields a linear time algorithm for computing the string kernel, shown in Supplementary Algorithm 5 and illustrated in Supplementary Fig. 8. Computing the kernel for a data matrix of n individuals requires n^2 such similarity evaluations. For further optimisation, we implemented a DP version of Supplementary Algorithm 5 that can be vectorised across samples and adapted for efficient computation of CovRSK.

Supplementary Algorithm 5: String kernel with triangular numbers pseudo-code

Input: Feature vectors x, x'

`/* Find all contiguous matches in` x `and` x'`,` μ`, and their lengths,` λ_μ `*/`

`/* For each match, compute its triangular number,` $T(\lambda_\mu)$ `*/`

`/* Sum up the output values,` $K(x, x') = \sum_\mu T(\lambda_\mu)$ `*/`

Result: String kernel similarity, $K(x, x')$

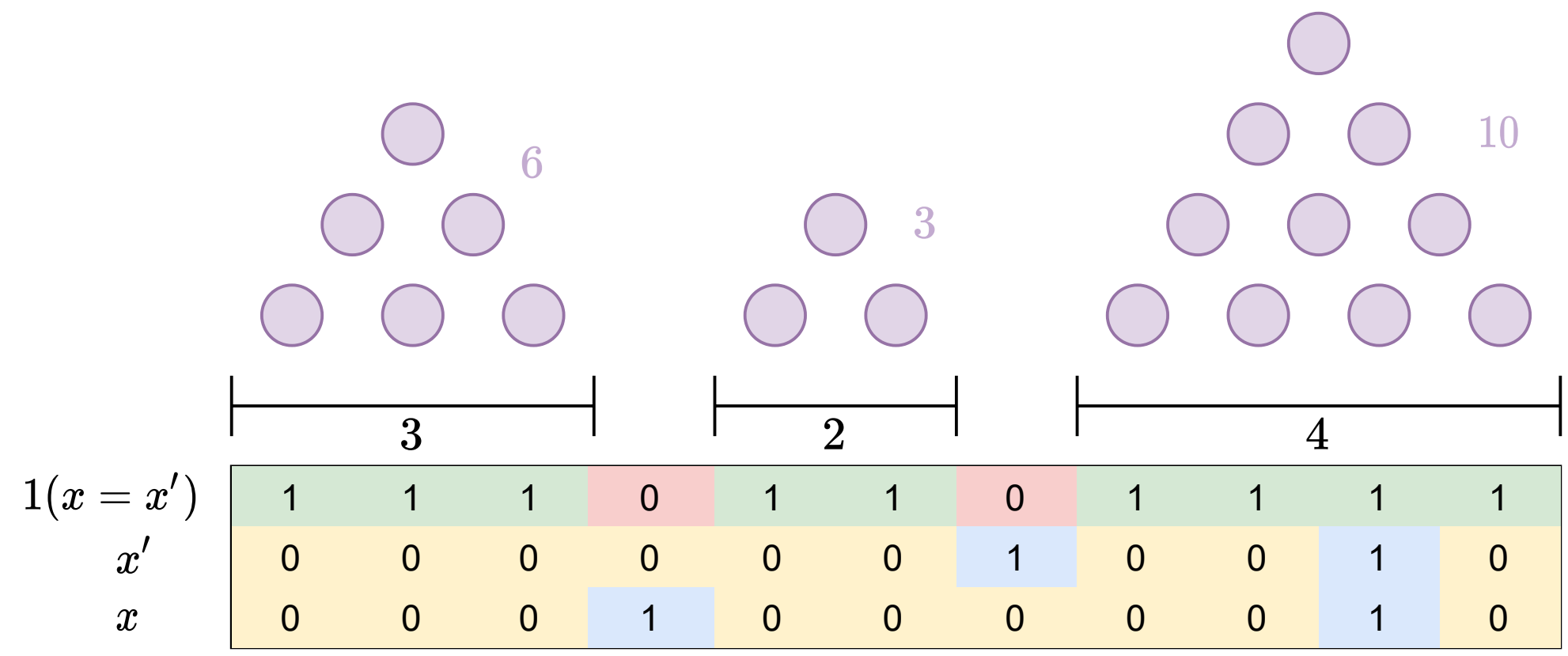

$$K(x, x') = T_3 + T_2 + T_4 = \frac{3(3+1)}{2} + \frac{2(2+1)}{2} + \frac{4(4+1)}{2} = 6 + 3 + 10 = 19$$

Supplementary Fig. 8. String kernel computation using triangular numbers. Illustration of the triangular-number formulation for efficient string kernel computation for two binary sequences x and x', each of length 11. Contiguous matching segments between x and x' are identified. A matching segment of length λ contains $T(\lambda) = \lambda(\lambda+1)/2$ matching contiguous k-mers across all $k \leq \lambda$. In this example, the sequences contain three matching segments of lengths 3, 2, and 4, contributing $T(3) = 6$, $T(2) = 3$, and $T(4) = 10$, respectively. Summing these contributions yields the string kernel value $K(x, x') = 19$ without explicitly enumerating every matching k-mer.

String kernel generalisation and kernel comparison

The string kernel computation can be viewed as a summation over polynomials,

$$K(x, x') = \sum_{\mu} g(\lambda),$$

where we identify all contiguous matches μ of the input sequences, determine their lengths λ, evaluate the polynomial $g(\lambda)$ at each length, and sum the results. In the canonical case,

$$g(\lambda) = T(\lambda) = \frac{\lambda(\lambda + 1)}{2} = \frac{1}{2}\lambda^2 + \frac{1}{2}\lambda.$$

This raises the question of whether this specific polynomial is optimal, or whether alternative functions may be better suited to the task. For instance, the Hamming kernel – historically popular in bioinformatics [2] – given by

$$H(x, x') = \sum_{i=1}^{M} I(x_i = x'_i),$$

can be computed in the same manner using the trivial linear polynomial $g(\lambda) = h(\lambda) = \lambda$. Intuitively, the choice of polynomial controls the contribution of longer matches relative to shorter ones. In the case of the Hamming kernel, longer matching sequences have no more importance than shorter ones, whereas the canonical string kernel weights sequence length contributions quadratically. This observation motivates a generalisation of the string kernel in which the triangular number polynomial $T(\lambda)$ is replaced by an arbitrary polynomial $g(\lambda)$. We refer to this generalisation as the polynomial string kernel and describe it in Supplementary Algorithm 6.

We investigate the optimality of the string kernel empirically in Supplementary Fig. 9. Specifically, we plot the validation accuracy of an SVM using different instances of the polynomial string kernel defined by monomials with degrees between one and two. We observe that neither the Hamming kernel, $g(\lambda) = \lambda$, nor the string kernel, $g(\lambda) = \frac{\lambda(\lambda+1)}{2} = \frac{1}{2}\lambda^2 + \frac{1}{2}\lambda \approx \lambda^2$, is optimal. Instead, the optimal degree for a monomial instance of the Polynomial string kernel is approximately $g(\lambda) = \lambda^{1.2}$, nearly matching the accuracy of CovRSK.

Supplementary Algorithm 6: Polynomial string kernel pseudo-code

Input: Feature vectors x, x' and a polynomial g

/* Find all contiguous matches in x and x', μ, and their lengths, λ_μ */

/* For each match, compute a polynomial of the lengths, $g(\lambda_\mu)$ */

/* Sum up the output values, $K_g(x, x') = \sum_\mu g(\lambda_\mu)$ */

Result: Polynomial String kernel similarity, $K_g(x, x')$

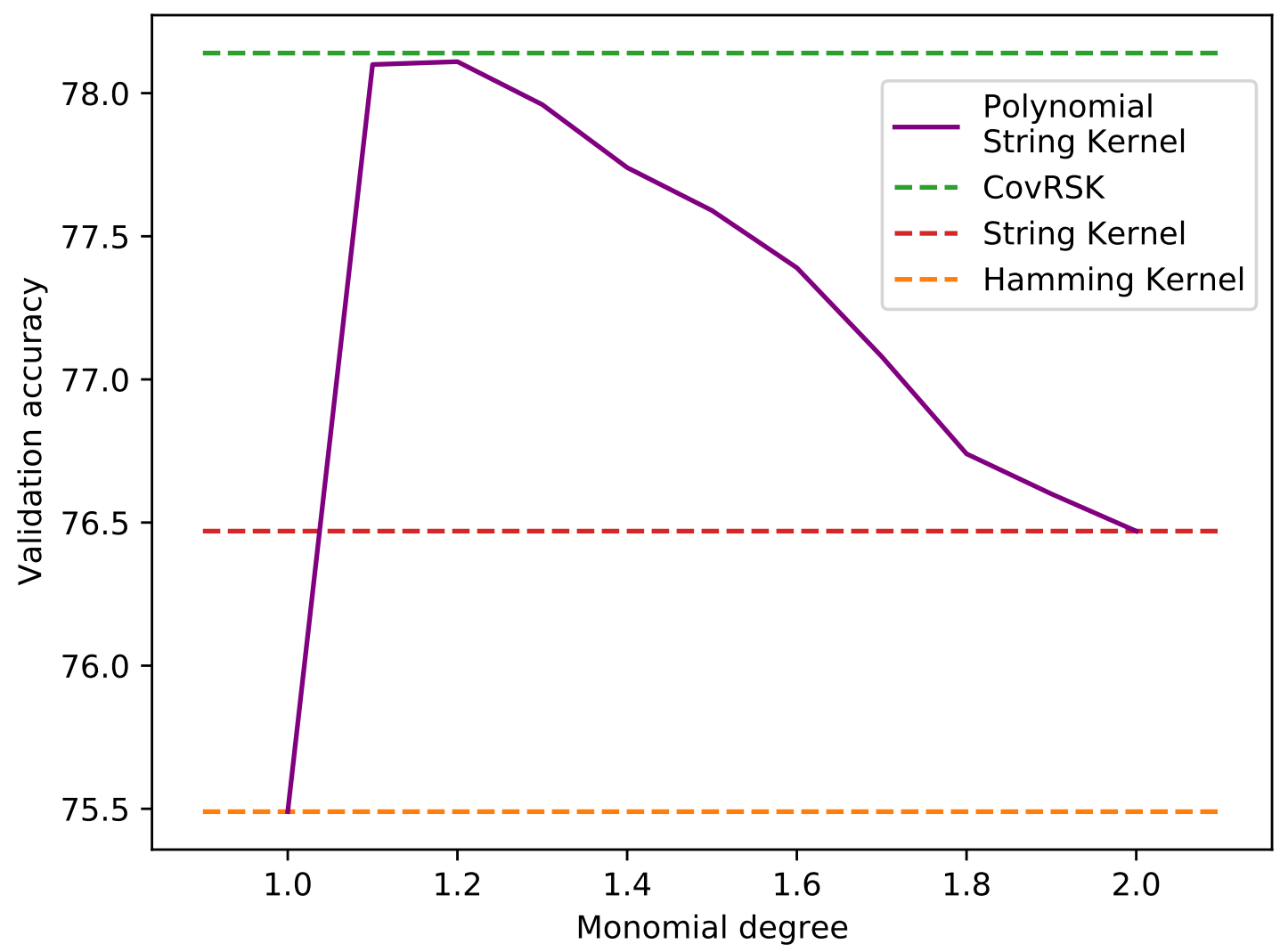

Supplementary Fig. 9. Comparison of polynomial string kernels. Validation accuracies of SVMs on the development dataset as a function of the monomial degree of the polynomial string kernel. Horizontal lines indicate the accuracies achieved by the Hamming kernel, the canonical string kernel, and CovRSK. The optimal monomial degree lies between 1 and 2, suggesting that neither the Hamming kernel nor the canonical string kernel is optimal. CovRSK achieves the highest accuracy overall, closely followed by the best-performing polynomial string kernel.

CovRSK theory and extended experiments

String kernel features as binomial random variables

Assuming that the input features (SNPs in this case) are binary, as well as independent and identically distributed,

$$p(x_i = 1) = p(x_i' = 1) = p \quad \forall i = 1, \ldots, M,$$

then the probability that a given feature is the same for two individuals is

$$q = p(x_i = x_i') = p^2 + (1-p)^2.$$

The event that a given subsequence of length 1 is equal for two individuals is clearly Bernoulli distributed and thus the number of subsequences of length 1 is binomial, $S_1 \sim$ Binomial(M,q) where M is sequence length. The expected value and variance are then given by

$$\mathbb{E}[S_1] = Mq, \quad \mathrm{Var}(S_1) = Mq(1-q).$$

Given our assumptions, two subsequences of length k will be equal with probability q given that the first $k-1$ elements are equal and thus,

$$S_k \mid S_{k-1} \sim \mathrm{Binomial}(S_{k-1}, q) \tag{1}$$

with expected value and variance,

$$\mathbb{E}[S_k \mid S_{k-1}] = qS_{k-1}, \quad \mathrm{Var}(S_k \mid S_{k-1}) = S_{k-1}q(1-q)$$

and therefore,

$$\mathbb{E}[S_k] = \mathbb{E}[\mathbb{E}[S_k|S_{k-1}]] = \mathbb{E}[qS_{k-1}] = q\mathbb{E}[S_{k-1}] = \ldots = q^{k-1}\mathbb{E}[S_1] = q^k M$$

and

$$\mathbb{E}[\mathrm{Var}(S_k \mid S_{k-1})] = \mathbb{E}[S_{k-1}q(1-q)] = q(1-q)\mathbb{E}[S_{k-1}] = q^k(1-q)M.$$

We visualise the last expression (normalised by the sequence length) in Supplementary Fig. 10.

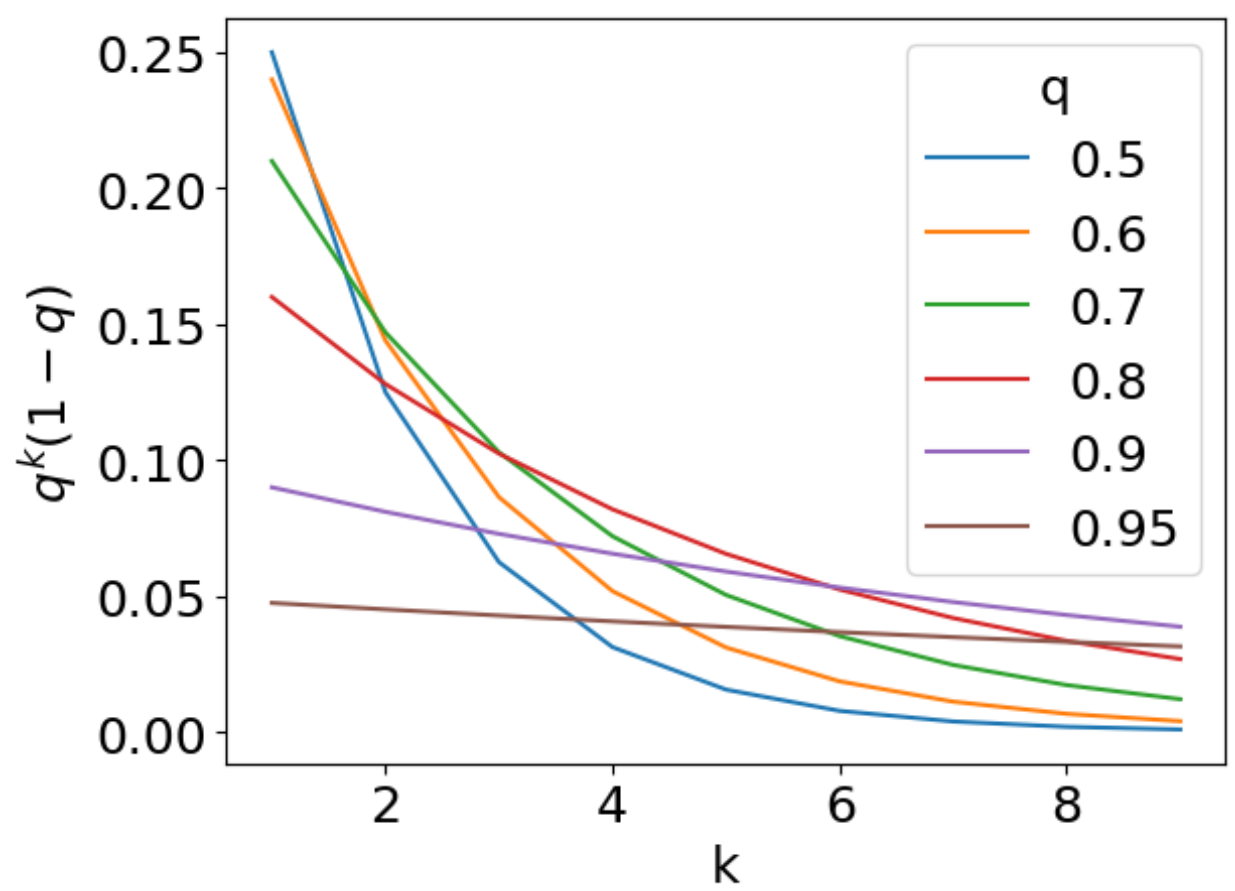

Supplementary Fig. 10. Expected conditional variance of string kernel features. Expected conditional variance of the number of matching subsequences of length k, normalised by sequence length M. Curves show $q^k(1-q)$ for match probabilities $q \in \{0.5, 0.6, 0.7, 0.8, 0.9, 0.95\}$, where $q = p^2 + (1-p)^2$ is the probability that two features match. The exponential decay with increasing k indicates that longer matching subsequences become progressively less variable under the null model.

String kernel feature covariance

By modelling the string kernel features as binomials, the expected value is given by

$$\mathbb{E}[S_k|S_{k-1}] = qS_{k-1}.$$

The covariance between adjacent string kernel features can therefore be computed using the law of total expectation,

$$\begin{aligned}
\mathrm{Cov}(S_k, S_{k+1}) &= \mathbb{E}[S_k S_{k+1}] - \mathbb{E}[S_k]\mathbb{E}[S_{k+1}] \\
&= \mathbb{E}[S_k \mathbb{E}[S_{k+1} \mid S_k]] - q\mathbb{E}[S_k]^2 \\
&= q\mathbb{E}[S_k^2] - q\mathbb{E}[S_k]^2 \\
&= q\left(\mathbb{E}[S_k^2] - \mathbb{E}[S_k]^2\right) \\
&= q\,\mathrm{Var}(S_k).
\end{aligned}$$

Using the recurrence in Supplementary Equation (1), this is easily extended to obtain the covariance between two arbitrary string kernel features,

$$\mathrm{Cov}(S_k, S_{k+j}) = q^j\,\mathrm{Var}(S_k). \tag{2}$$

String kernel feature correlation

By modelling the string kernel features as binomial random variables, the expected value is given by

$$\mathbb{E}[S_k|S_{k-1}] = qS_{k-1}.$$

As shown in Supplementary Equation (2), the covariance between two string kernel features is

$$\mathrm{Cov}(S_k, S_{k+j}) = q^j\,\mathrm{Var}(S_k).$$

A lower bound on the correlation can then be computed using the law of total variance,

$$\begin{aligned}
\rho_{S_k,S_{k+j}} &= \frac{\mathrm{Cov}(S_k, S_{k+j})}{\sqrt{\mathrm{Var}(S_k)\,\mathrm{Var}(S_{k+j})}} \\
&= q^j\sqrt{\frac{\mathrm{Var}(S_k)}{\mathrm{Var}(S_{k+j})}} \\
&= q^j\sqrt{\frac{\mathbb{E}[\mathrm{Var}(S_k \mid S_{k-1})] + \mathrm{Var}(\mathbb{E}[S_k \mid S_{k-1}])}{\mathrm{Var}(S_{k+j})}} \\
&= q^j\sqrt{\frac{\mathbb{E}[\mathrm{Var}(S_k \mid S_{k-1})] + \mathrm{Var}(qS_{k-1})}{\mathrm{Var}(S_{k+j})}} \\
&= q^j\sqrt{\frac{\mathbb{E}[\mathrm{Var}(S_k \mid S_{k-1})] + q^2\,\mathrm{Var}(S_{k-1})}{\mathrm{Var}(S_{k+j})}} \\
&\geq q^j\sqrt{\frac{q^2\,\mathrm{Var}(S_{k-1})}{\mathrm{Var}(S_{k+j})}} \\
&= q^{j+1}
\end{aligned} \tag{3}$$

which is visualised in Supplementary Fig. 11.

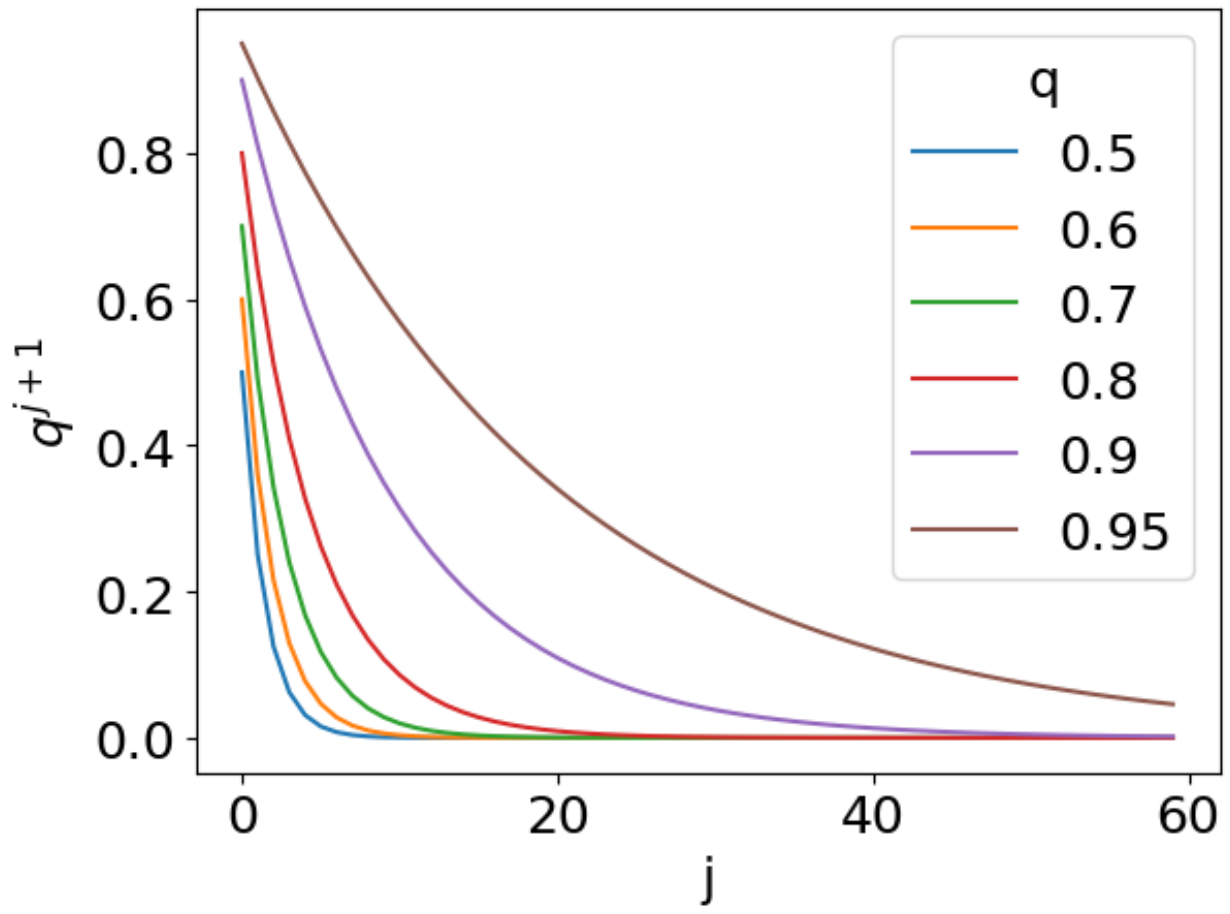

Supplementary Fig. 11. Lower bound on string kernel feature correlation. Lower bound on the correlation between string kernel features separated by j subsequence lengths under the independent Bernoulli model. Curves show q^{j+1} for match probabilities $q \in \{0.5, 0.6, 0.7, 0.8, 0.9, 0.95\}$, where $q = p^2 + (1-p)^2$ is the probability that two features match. The lower bound decreases exponentially with feature distance j.

String kernel thought experiment

To provide more insight, we demonstrate a key difference between CovRSK and the standard string kernel using an example, which is visualised in Supplementary Fig. 12. Sample b shares only 50% of its SNPs with the example reference sample (the first half matches the reference exactly), while sample e shares 99.5% of its SNPs with the reference sample (but has shorter contiguous subsequences of exact match than the first half of sample b). Sample e has the highest CovRSK similarity to the reference sample, but the string kernel ranks sample b to be the most similar. This is due to the accumulation of the correlated string kernel features. As the contiguous sequence match gets longer, the number of total k-mers matching grows by the length of the contiguous match (one for each length, from one to the full contiguous match length) meaning that the match's overall contribution to the similarity score grows as $O(n^2)$ with its length. As a result of this nonlinearity, the long contiguous sequence match of sample b contributes more than the sum of the many – but shorter – sequence matches of sample e. This characteristic of the string kernel is not necessarily undesirable and might in some cases even be desired. However, given the stochastic nature of genetic inheritance and potential for noisy sequencing or genotyping, one cannot expect sequences of related individuals in a population to share the exact same contiguous sequence, even though the two individuals may have inherited the contiguous sequence identically-by-descent from a common ancestor. It is more likely that the sequences of the two individuals' genomes stemming from the same population will have very low variance in general. Therefore, the stochasticity of the contiguous length of the shared sequence, along with the quadratically increasing influence of segment length on the string kernel, could potentially lead to overfitting to the training samples. In general, CovRSK is competitive with the standard string kernel for best accuracy, beating all other methods, with the top spot trading (narrowly) between them depending on the smoother used; however, in all cases, the CovRSK is far faster than the regular string kernel.

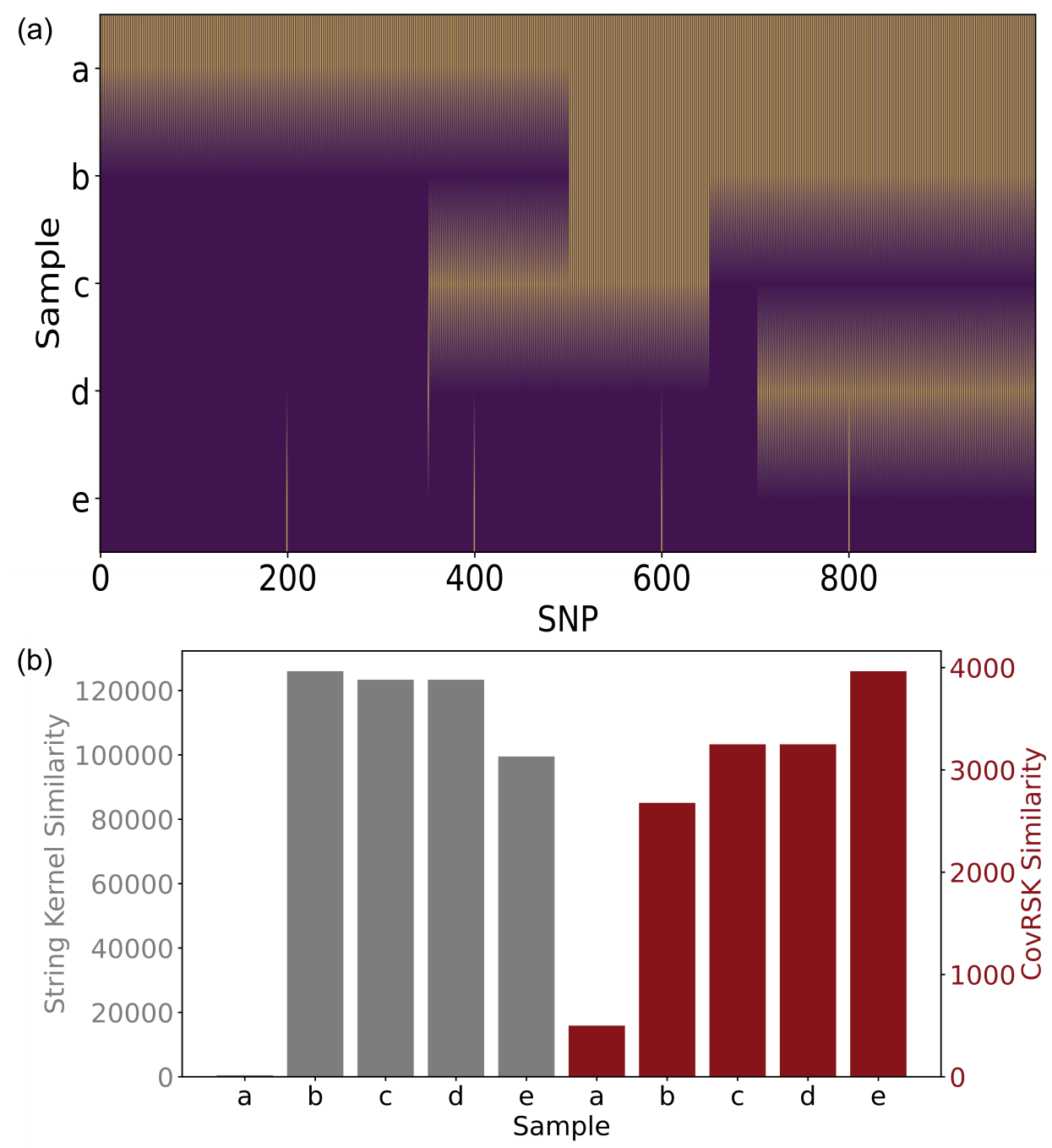

Supplementary Fig. 12. CovRSK thought experiment. a Toy examples of SNP-level matches to a reference sample, where purple indicates matching SNPs and yellow indicates mismatches. Sample b contains one long contiguous matching segment, whereas sample e has many shorter matching segments despite a higher overall SNP match rate. **b** Similarity scores between each sample and the reference sample computed using the standard string kernel (grey) and CovRSK with $\alpha = 0.9$ and $\beta = 0.5$ (red).

Empirical comparison of CovRSK and the standard string kernel

In Supplementary Fig. 13, we show a comparison between CovRSK and the string kernel similar to the example above, but this time with real samples. Again, the samples are coloured so that purple represents SNPs matching the reference sample and yellow denotes mismatches. The only sample actually sharing ancestry with the reference sample is sample four. We notice how CovRSK correctly returns that sample as the most similar, while the string kernel incorrectly scores sample seven higher.

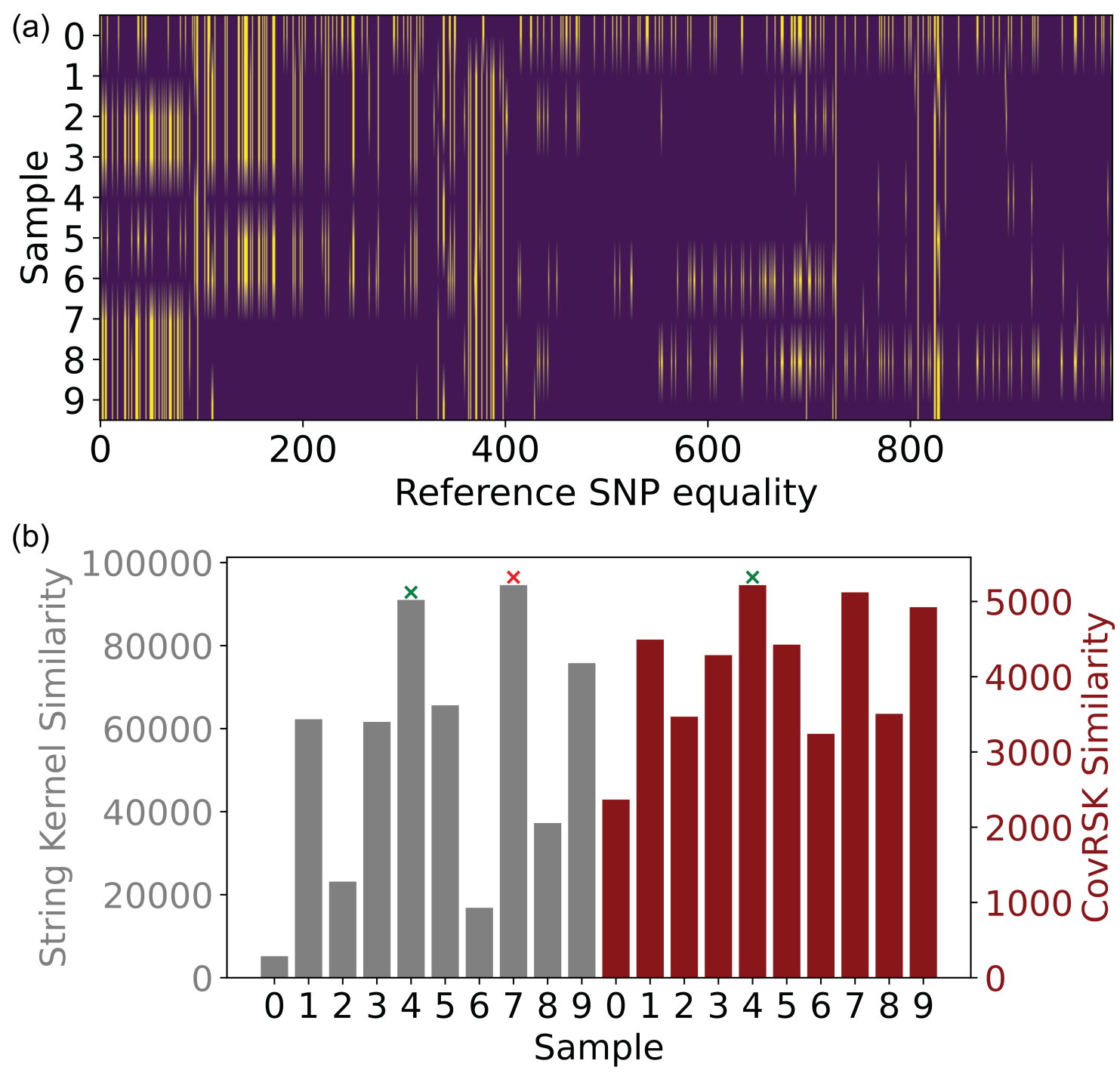

Supplementary Fig. 13. Empirical comparison of standard string kernel and CovRSK similarity. a SNP-level matches between a reference sample and ten empirical samples from the 7-ancestry dataset, where purple indicates SNPs matching the reference sample and yellow indicates mismatches. Only sample 4 has the same ancestry as the reference. **b** Similarity scores between each sample and the reference sample computed using the standard string kernel (grey) and CovRSK with $\alpha = 0.9$ and $\beta = 0.5$ (red). CovRSK assigns the highest similarity to sample 4, whereas the standard string kernel assigns the highest similarity to sample 7.

To provide insight into the CovRSK sampling algorithm, an instance on a sequence of length 300 is visualised in Supplementary Fig. 14. The solid blue line represents the probability of sampling a k SNP sequence given the sampling history. The dashed line represents the decaying probability without covariance reduction. As the subsequences get larger, the sampling probability decreases in general. When a k-length sequence is sampled, the probability of sampling the next higher k drops and slowly rises as the distance increases.

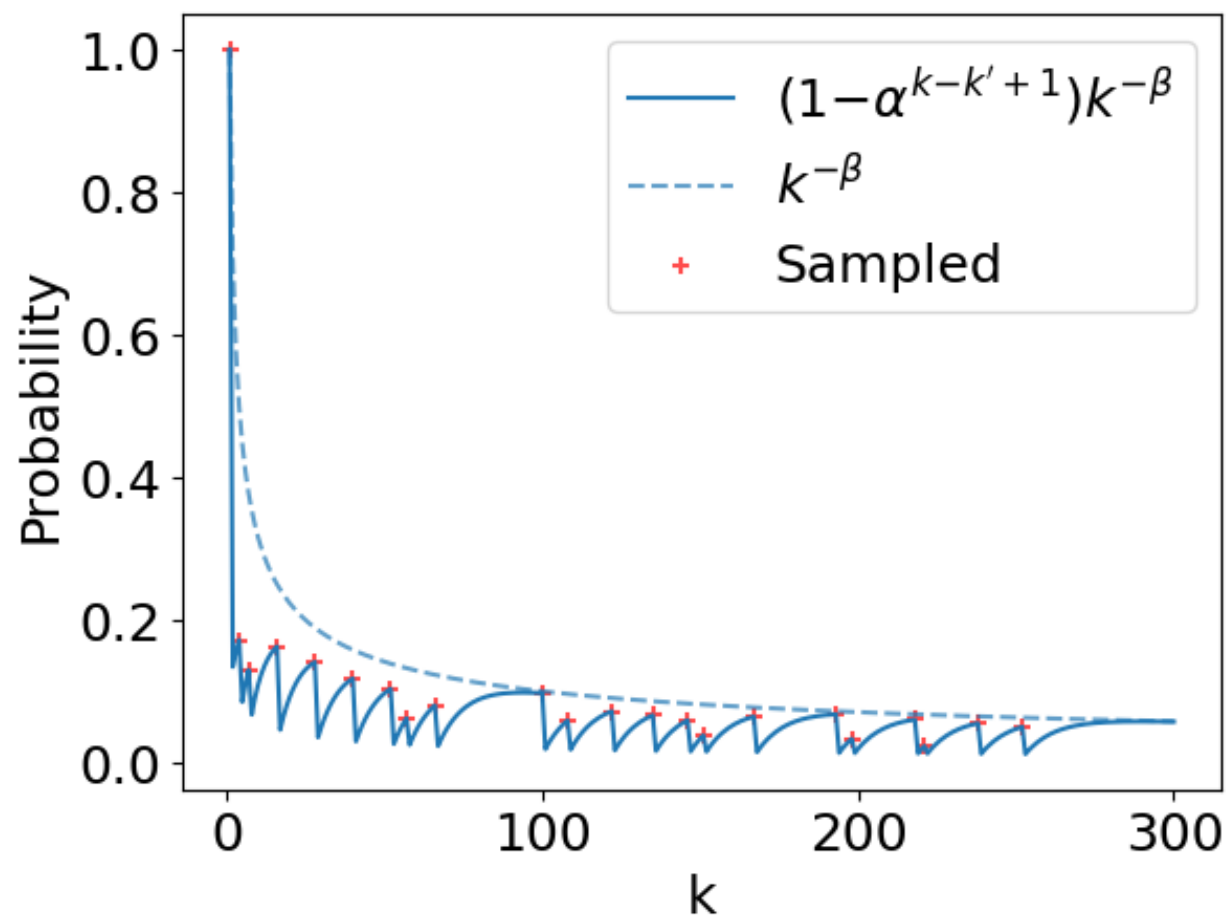

Supplementary Fig. 14. CovRSK feature sampling probabilities. Example of CovRSK feature sampling for a sequence of length 300 using $\alpha = 0.9$ and $\beta = 0.5$. The solid blue curve shows the probability of sampling a k SNP sequence given the sampling history, and red markers indicate sampled subsequence lengths. The dashed curve shows the baseline decay $k^{-\beta}$ without covariance reduction.

Supplementary References

[1] Kumar, A., Liang, P. & Ma, T. Verified uncertainty calibration. In *Proceedings of the 33rd International Conference on Neural Information Processing Systems*, 3792–3803 (2019).

[2] Pérez-Reche, F. J., Rotariu, O., Lopes, B. S., Forbes, K. J. & Strachan, N. J. Mining whole genome sequence data to efficiently attribute individuals to source populations. *Scientific reports* **10**, 1–16 (2020).
