# Supplementary material for: Scalable high resolution ancestry deconvolution for genomic data
Source: Nat Commun. 2026 Jul 25;17:9070. doi: 10.1038/s41467-026-75391-0 (PMC13518915; doi:10.1038/s41467-026-75391-0)
Supplement: Supplementary file 2 [file 41467_2026_75391_MOESM2_ESM.pdf]

nature portfolio

Corresponding author(s): Alexander G. Ioannidis

Last updated by author(s): Jun 3, 2026

Reporting Summary

Nature Portfolio wishes to improve the reproducibility of the work that we publish. This form provides structure for consistency and transparency in reporting. For further information on Nature Portfolio policies, see our Editorial Policies and the Editorial Policy Checklist.

Statistics

For all statistical analyses, confirm that the following items are present in the figure legend, table legend, main text, or Methods section.

n/a	Confirmed	
☐	☒	The exact sample size (*n*) for each experimental group/condition, given as a discrete number and unit of measurement
☐	☒	A statement on whether measurements were taken from distinct samples or whether the same sample was measured repeatedly
☒	☐	The statistical test(s) used AND whether they are one- or two-sided<br>*Only common tests should be described solely by name; describe more complex techniques in the Methods section.*
☒	☐	A description of all covariates tested
☐	☒	A description of any assumptions or corrections, such as tests of normality and adjustment for multiple comparisons
☐	☒	A full description of the statistical parameters including central tendency (e.g. means) or other basic estimates (e.g. regression coefficient) AND variation (e.g. standard deviation) or associated estimates of uncertainty (e.g. confidence intervals)
☒	☐	For null hypothesis testing, the test statistic (e.g. *F*, *t*, *r*) with confidence intervals, effect sizes, degrees of freedom and *P* value noted<br>*Give P values as exact values whenever suitable.*
☒	☐	For Bayesian analysis, information on the choice of priors and Markov chain Monte Carlo settings
☒	☐	For hierarchical and complex designs, identification of the appropriate level for tests and full reporting of outcomes
☒	☐	Estimates of effect sizes (e.g. Cohen's *d*, Pearson's *r*), indicating how they were calculated

Our web collection on statistics for biologists contains articles on many of the points above.

Software and code

Policy information about availability of computer code

Data collection	Human genomic datasets used in this study were obtained from publicly available sources. Genomic data were downloaded directly from the respective repositories.
Data analysis	UCSC liftOver: genome coordinate harmonization to build 37<br>Beagle v5.1: haplotype phasing<br>KING: removal of related individuals (up to second degree)<br>ADMIXTURE: unsupervised clustering to identify single-ancestry individuals<br>Gnomix (https://github.com/AI-sandbox/gnomix/): local ancestry inference and Gnofix phasing correction<br>RFMix (v2): local ancestry inference benchmarking<br>ELAI (https://github.com/haplotype/elai): local ancestry inference benchmarking<br>Loter (https://github.com/bcm-uga/Loter): local ancestry inference benchmarking<br>FLARE (https://github.com/browning-lab/flare): local ancestry inference benchmarking<br>Orchestra (https://github.com/omicsedge/orchestra-paper): local ancestry inference benchmarking<br>Core libraries: numpy, pandas, scikit-learn, scikit-allel, scipy, xgboost, matplotlib, seaborn, tqdm (versions in requirements.txt)

For manuscripts utilizing custom algorithms or software that are central to the research but not yet described in published literature, software must be made available to editors and reviewers. We strongly encourage code deposition in a community repository (e.g. GitHub). See the Nature Portfolio guidelines for submitting code & software for further information.

Data

Policy information about availability of data

All manuscripts must include a data availability statement. This statement should provide the following information, where applicable:

- Accession codes, unique identifiers, or web links for publicly available datasets
- A description of any restrictions on data availability
- For clinical datasets or third party data, please ensure that the statement adheres to our policy

1000 Genomes Project: https://www.internationalgenome.org
Human Genome Diversity Project (HGDP): https://www.internationalgenome.org/data-portal/data-collection/hgdp
Simons Genome Diversity Project (SGDP): https://www.simonsfoundation.org/simons-genome-diversity-project/

All genomic data were downloaded directly from these repositories and processed as described in the Methods.

Research involving human participants, their data, or biological material

Policy information about studies with human participants or human data. See also policy information about sex, gender (identity/presentation), and sexual orientation and race, ethnicity and racism.

Reporting on sex and gender	This work did not involve recruitment of human participants. Demographic information (including sex where available) is derived from publicly available datasets (1000 Genomes Project, HGDP, SGDP), as described in the original studies.
Reporting on race, ethnicity, or other socially relevant groupings	Population labels correspond to genetic ancestry groups defined for analysis purposes (e.g., African, European, East Asian, etc.) and are based on reference population structure from publicly available datasets. These labels reflect genetic clustering rather than socially constructed categories.
Population characteristics	All analyses were conducted using publicly available genomic data from the 1000 Genomes Project, HGDP, and SGDP. Sample characteristics, including geographic origin and population labels, are described in the original publications of these datasets.
Recruitment	No participants were recruited for this study. All data were obtained from publicly available resources.
Ethics oversight	All genomic data used are publicly available and were collected under the ethical approvals described in the original studies (1000 Genomes Project, HGDP, SGDP). No additional ethical approval was required for this secondary analysis.

Note that full information on the approval of the study protocol must also be provided in the manuscript.

Field-specific reporting

Please select the one below that is the best fit for your research. If you are not sure, read the appropriate sections before making your selection.

☒ Life sciences ☐ Behavioural & social sciences ☐ Ecological, evolutionary & environmental sciences

For a reference copy of the document with all sections, see nature.com/documents/nr-reporting-summary-flat.pdf

Life sciences study design

All studies must disclose on these points even when the disclosure is negative.

Sample size	No statistical methods were used to predetermine sample size. The study uses selected publicly available genomic reference datasets (e.g., 1000 Genomes Project, HGDP, SGDP). We sought to include sufficient sample to power statistical comparisons.
Data exclusions	Variants were restricted to biallelic SNPs. Samples underwent standard genomic QC:<br><br>Removal of second-degree and closer relatives using KING<br>Filtering to retain single-ancestry individuals defined as ≥95% assignment probability to a single ancestry cluster (via ADMIXTURE)<br>Harmonization to a common genome build (GRCh37)<br>Exclusion of ancestry groups with insufficient sample size
Replication	Random seeds were fixed for all stochastic components, including data simulation and model training, where applicable. This ensures that repeated runs produce identical results across all analyses.
Randomization	Founder individuals were randomly split into training and testing sets, and simulated admixed individuals were generated by random sampling of ancestry segments and recombination breakpoints. Cohorts were defined by ancestry composition, with balanced representation across generations since admixture.
Blinding	Blinding was not applicable, as analyses were conducted on simulated and publicly available genomic data with predefined labels, and no subjective assessment or manual group assignment was involved.

Reporting for specific materials, systems and methods

We require information from authors about some types of materials, experimental systems and methods used in many studies. Here, indicate whether each material, system or method listed is relevant to your study. If you are not sure if a list item applies to your research, read the appropriate section before selecting a response.

Materials & experimental systems

n/a	Involved in the study
☒	☐ Antibodies
☒	☐ Eukaryotic cell lines
☒	☐ Palaeontology and archaeology
☒	☐ Animals and other organisms
☒	☐ Clinical data
☒	☐ Dual use research of concern
☒	☐ Plants

Methods

n/a	Involved in the study
☒	☐ ChIP-seq
☒	☐ Flow cytometry
☒	☐ MRI-based neuroimaging

Plants

Seed stocks	*Report on the source of all seed stocks or other plant material used. If applicable, state the seed stock centre and catalogue number. If plant specimens were collected from the field, describe the collection location, date and sampling procedures.*
Novel plant genotypes	*Describe the methods by which all novel plant genotypes were produced. This includes those generated by transgenic approaches, gene editing, chemical/radiation-based mutagenesis and hybridization. For transgenic lines, describe the transformation method, the number of independent lines analyzed and the generation upon which experiments were performed. For gene-edited lines, describe the editor used, the endogenous sequence targeted for editing, the targeting guide RNA sequence (if applicable) and how the editor was applied.*
Authentication	*Describe any authentication procedures for each seed stock used or novel genotype generated. Describe any experiments used to assess the effect of a mutation and, where applicable, how potential secondary effects (e.g. second site T-DNA insertions, mosiacism, off-target gene editing) were examined.*
