# Supplementary material for: Scalable high resolution ancestry deconvolution for genomic data
Source: Nat Commun. 2026 Jul 25;17:9070. doi: 10.1038/s41467-026-75391-0 (PMC13518915; doi:10.1038/s41467-026-75391-0)
Supplement: Supplementary file 3 [file 41467_2026_75391_MOESM3_ESM.pdf]

nature portfolio

Peer Review File

High Resolution Ancestry Deconvolution for Next Generation Genomic Data

Corresponding Author: Professor Alexander Ioannidis

This file contains all reviewer reports in order by version, followed by all author rebuttals in order by version.

Version 0:

Reviewer comments:

Reviewer #1

(Remarks to the Author)
This manuscript presents a new set of algorithms for local ancestry inference (LAI). The G-Nomix LAI tool is reported to achieve higher accuracy and greater speed than the widely used RFMix LAI program as well as the ELAI and Loter methods. The speed increase in particular is crucially important for the research community, as RFMix is painfully slow and does not scale to large, biobank-scale genomic datasets. The companion Gnofix tool provides for rapid and accurate phase correction. The paper is very well written, and the figures are outstanding; the algorithm is comprehensively documented, and the choices are well justified; benchmarking appears to have been comprehensive. Accordingly, I recommend that the manuscript be accepted for publication once my comments are addressed. Since I am a user of ancestry inference methods, rather than a developer, I will focus my comments on the programs' utility as opposed to the details of the algorithm. In addition to my comments on the manuscript, I provide discretionary suggestions for additional information that could make the GitHub repository even more useful, thereby (potentially) increasing community uptake of the method.

Major points

1. The Brownings recently developed the LAI program FLARE https://www.biorxiv.org/content/10.1101/2022.08.02.502540v1, which reportedly also provides high accuracy and speed for large datasets. It would be helpful to compare the performance of Gnomix to FLARE.

2. Gnomix is benchmarked with highly divergent continental ancestry groups. It would be of great interest to know how the program performs on more closely related subcontinental ancestry groups. For example, could the method distinguish Northern European, Southern European, and Finnish haplotypes? This could be particularly useful given the motivation of local ancestry informed GWAS methods in light of the challenges that even fine-scale population structure pose to GWAS. Or is the method currently limited to continental-level ancestry deconvolution?

3. Gnomix is benchmarked using simulated datasets, which is standard and entirely reasonable given the need for a ground-truth against which to compare. Nevertheless, I wonder if it would be possible to test on at least a few trios of admixed genomes using the pre-trained models available on their GitHub repository. For example, AMR trios from the 1000 Genomes Project.

4. Gnomix is benchmarked using a single (relatively small) chromosome, which again is quite reasonable given the comprehensiveness of the benchmarking performed. Nevertheless, one of the big selling points of the method is its speed and applicability to biobank-scale datasets. It would be very nice to see the performance results of applying this method to a large biobank scale data set of whole genome data sets (either sequences or genotypes) such as UK Biobank or All of Us.

4a. A corollary point is that it is difficult to make out the actual speed of their two preferred methods from Figure 4, which is very dense as many methods are evaluated. A simple visual representation of the time of running their two best performing algorithms programs on X thousands of genomes, compared to ELAI and Loter (and perhaps FLARE), would be very helpful.

Minor points

5. How many genomes (chromosome 20s) were simulated for the benchmarking tables (Appendix D)? Is that what size

refers to in Table 1?

6. What were the computational resources used for benchmarking, e.g. RAM and cores used?

7. Why does the Latin American dataset contain so many more AFR founders, compared to EUR and NAT?

8. The manuscript mentions that larger windows may span ancestry switch points. Does the use of the smoother algorithm have the same potential resolution problem? In other words, how can one be sure that the smoother does not artificially concatenate same-ancestry haplotypes that actually come from different chromosomes? Is this one source of the error seen for higher generation time since admixture runs?

Discretionary GitHub suggestions

The GitHub is detailed and very useful, and the Jupyter notebook demo is a nice addition. Accordingly, these comments are purely discretionary for the authors' consideration.

9. It would be very nice to know what kind of data QC should be done before running the program, e.g. should LD and/or MAF pruning be performed?

10. Similarly, data QC recommendations for model training would be helpful. Should LD pruned reference datasets be used? Should 10M SNPs or 100,000 SNPs be used for training?

11. Large datasets often run into too much memory consumption (e.g. Beagle for phasing). It would be nice to know how program parameters affect memory consumption. Memory consumption is mentioned for two parameters, but not for window_size, smooth size etc.

12. For the window_size_cM parameter, it states "use larger windows if snp density is lower e.g. genotype data vs. sequence (default .5)". This is helpful but specific parameter recommendations based on the type of data would be nice. For example, what parameters should be used for WGS data from All of Us versus genotype data from the UK Biobank?

13. In general, recommendations on what parameter values to use according to the number of SNPs & samples, and perhaps what computational resources should be enough for them, would be helpful. For example, for running Gnomix on the All of Us platform, should 4 cores and 32 GB RAM or 40 cores and 350GB RAM be requested. Research of this kind is increasingly cloud-based, with charges by the hour for every resource used. How does running Gnomix on 10,000 samples vs 100,000 samples impact the computational resource requirement?

Reviewer #2

(Remarks to the Author)
Hilmarsson et al developed a new local ancestry inference method. The authors use simulated data to demonstrate that their new method is faster, and more accurate than the current state of the art local ancestry inference methods. The authors also present a new method to correct for phasing errors using local ancestry results, which has similar accuracy to RFMix but is significantly quicker. The authors also apply their method to a dataset of dogs, and demonstrated that admixture between European and pre-Columbian dogs occurred in a similar time frame to the admixture of Native American and European humans in Mexico. These improvements to local ancestry inference will be very beneficial due to the increasing size of diverse genetic datasets. Therefore, this method has the potential to be very impactful on the field of genomics and population genetics. I only have minor comments/suggestions for the authors to address.
1. Is there a figure that shows the results of the ADMIXTURE analysis mentioned in section 3.1? What cut-off was used to determine an individual as a single ancestry individual? Were any variant filters applied before running admixture, such as LD pruning or minor allele frequency filters?
2. When running RFMix in figure 4D, did you change the parameter for time since admixture to match the simulated value? If you kept this parameter as the RFMix default value, how might that impact the accuracy of RFMix on your simulated data?
3. I think more details are required in the methods or results section for the general reader to understand how fitting an exponential curve to the distribution of local ancestry segment lengths results in being able to estimate the time of admixture.
4. Page 8 lines 279-281. What figures/tables support this observation? Can you cite them here?
5. What parameters were used when phasing with Beagle? Was a genetic map used and/or a reference panel?
6. How were the human genetic datasets transferred to GRCh37? Was a program like liftover and the UCSC genome browser chain files used?
7. Can you estimate how much of the Chihuahua genome is identified as being from Coyotes, similar to how you estimate the amount of arctic ancestry in Figure 8a?

Reviewer #3

(Remarks to the Author)
Hilmarsson et al present a new method for local ancestry inference (LAI) which they describe as as " flexible collection of new local ancestry algorithms".
I agree with authors that the time is ripe for new LAI methods, especially ones that scale to the large size of current

population genomics datasets. So it is good to have new methods being proposed that can handle large genetic datasets.
I see many positive aspects to the proposed method and manuscript. It claims higher accuracy and faster performance than some other methods, especially in many way (e.g. 7) admixtures. It can work on array or sequence data, it includes a phase correction step, and is open source and available. The manuscript is clearly written and contains a number of useful explanatory figures.
Towards the goal of developing scalable LAI methods suitable for biobank data sets, I find two main contributions from the current manuscript. First, the authors have developed a flexible framework for LAI using a broad array of (supervised and discriminative) classification methods. They do this by applying classifiers in genomic windows, with an additional smoothing step to better identify local ancestry tracts that span multiple windows. Second, the authors present a new kernel for a support vector machine (SVM), CovRSK. This kernel tries to address the problem of how much more weight should be given to matching contiguous genomic regions as the length of the match increases. Intuitively longer matches carry more information about ancestry, but how much more? CovRSK employs stochastic sampling to thin redundant features. CovRSK turns out to be most useful when the SNP data is not dense where it helps avoid overfitting.
I see how the flexibility offered by Gnomix can allow researchers to select an ideal tradeoff between speed and accuracy for their specific task and resources. However I worry a bit that this flexibility will more often be an overall net detriment to users, as external users may either not be sophisticated enough or willing to devote enough time to get it right.
The manuscript presents analyses of Gnomix applied to canid species. I'm not familiar with the canid literature, but I agree with the authors that ~90% accuracy across 12 ancestries is doing pretty well. It is notable how often the broad ancestry categories show up in both chromosomes of the Xoloitzcuintle dog in Figure 8b.

Major comments
The manuscript applies and tests a large number of different machine learning methods but the details of how each method is applied is not described. Many of the methods are sensitive to hyperparameters, such as learning rate , tree depth, objective function, etc, but these are not described in the manuscript.
I'm a bit concerned with how the superpopulations are divided into test and training data sets. The superpopulations are made up of (sub)populations and the individuals within each of these pops will be more closely related than to others in the superpopulation. This can cause biases if the data are naively split within each superpopulation into test and training sets, and will increase the variance across splits. A number of these superpopulations are small with small (sub)populations which can exacerbate the issue. Perhaps one (sub)population could be left out each time, not sure. To me this is one of the chief difficulties when using real data to validate methods.
In a related issue, I don't see any CI around the estimates of accuracy in tables 2-7, why not? My initial thought is the data split and model generating and LAI procedure could be repeated to generate a CI around the mean accuracy. CIs would then allow significant differences to be highlighted in the table rather than then larger of 97.79 vs 97.70 which I don't think are likely to be significantly different.
The authors do not compare their method to recent Li and Stephens HMM methods, e.g. MOSAIC in 2019 (https://doi.org/10.1534/genetics.119.302139) or FLARE in 2022 (https://doi.org/10.1101/2022.08.02.502540) and instead compare to ELAI from 2014. This is especially important as the MOSAIC paper showed it outperformed ELAI, and FLARE showed it outperformed MOSAIC with large reference sizes.
I found the main simulated admixture scenario presented in the main text somewhat unrealistic - e.g. a simultaneous 7-way admixture of equal proportions. This likely presents a challenging LAI problem, and is fine to include, but it doesn't really represent the majority of common LAI analyses and is the only result presented in the main text. The results from tables 2-7 should all be present in the main text, as a figure or table, maybe in a simplified form.
In the abstract the authors (correctly) note other LAI methods are burdened by "large next generation datasets" and "massive, whole genome biobanks". One of the main benefits of these large datasets are huge reference panels for supervised tasks such as LAI. However, despite the current manuscript describing "biobanks consisting of hundreds of thousands of individuals" as common, it does not attempt to show how Gnomix could be applied with reference panels approaching this size. The largest number of reference individuals seems to be 1290. In comparison, FLARE (https://doi.org/10.1101/2022.08.02.502540) considered reference panels up to 30K, and showed increasing accuracy with increasing ref sizes. Including larger reference panels would strengthen the claims in the manuscript that Gnomix is the method best suited to tackle "large next generation datasets".

Minor comments
Depending on the dataset, there is a real potential for related individuals among the human data. Are steps taken to identify and remove related individuals? This could be a problem if related individuals are split across the test and training data sets.
Versions of the programs compared to were not noted - e.g. was RFMIx v1 or V2 used?
How does the DEV data set seem to have more samples than are available for some populations? (WAS and NAT). Maybe I am misunderstanding table 1.
The application to Canids and using the distribution of local ancestry tract lengths is a nice result.
What does the size column in table 1 mean?
The authors state that Gnomix enables "portable models that are particularly useful when training data are not shareable due to privacy or other restrictions". But it was not clear what privacy guarantees (if any) are able to be provided for models trained by Gnomix. Is it guaranteed that no training data could be inferred from examination or application of the model? Would biobanks accept this level of privacy for their data? If the authors wish to retain this statement, it should be supported.
Sections 2.2.2 and 2.2.3 could be moved to the supplement. As is they take up some of the main focus of the paper (e.g. Figure 2) for an aspect of the paper not as directly related to local ancestry deconvolution.
I would prefer to see all of the evaluations applied to data which is not perfectly phased. This would involve an extra statistical rephasing step of the entire reference and target samples. Other recent LAI methods have done this (MOSAIC and FLARE) by considering diploid ancestry.
I would like to see ELAI and Loter included in the 3-way admixture analysis (called "Latin American"), as these are the most

realistic admixture scenarios.
Figure A1: The histogram could be normalized to allow easier comparison
Figure A4:The uncalibrated curves are mirrored across the diagonal to visually illustrate the centrality of the calibrated ones. - this is not helpful. Maybe break this plot out by ancestry. And perhaps provide some additional commentary on if this is useful.
Figure A7 and A8: This is a nice demonstration - but from my understanding the same ranking could be achieved with a simple score based on the fraction of matching sites (Hamming kernel), so this isn't quite an example of where CovRSK outperforms.
Figure A11 and A12: should be combined into one figure and share a color scheme. Too hard to compare as is.
Table 2 is this "whole genome" or just chromosome 20?. does "whole genome" mean sequencing like data?

Version 1:

Reviewer comments:

Reviewer #1

(Remarks to the Author)
I am satisfied with the authors' responses to my comments and recommend that the revised manuscript be accepted for publication.

(Remarks on code availability)

Reviewer #2

(Remarks to the Author)
Hilmarsson et al sufficiently replied and addressed all of my comments. Their revised manuscript demonstrates the utility of Gnomix and how Gnomix improves on previously published local ancestry software. Gnomix will be a great local ancestry tool for the research community. I do not have additional comments for the authors to address.

(Remarks on code availability)
The authors provided an extensive README and guide to help others install and use their code. This will greatly help their resource be used by members of the research community.

Reviewer #3

(Remarks to the Author)
The authors have thoroughly updated the manuscript and address all the major and minor concerns I had with the first version.

I have a few small comments.

In table 1, the timing unit is not specified, and training time is not include (I don't think). This leads to an inaccurate picture of the resources needed for an analysis, as GNOMIX preprocesses the data, while the other methods do not.

In figure 2. The broken axes on panels a and b make it really hard to compare, I would suggest the break is removed.

FLARE seems to be the fastest method and has higher accuracy than RFMIX, so why was it not shown in Figure 2D?

(Remarks on code availability)
The open source repo is well maintained and available.

Open Access This Peer Review File is licensed under a Creative Commons Attribution 4.0 International License, which permits use, sharing, adaptation, distribution and reproduction in any medium or format, as long as you give appropriate credit to the original author(s) and the source, provide a link to the Creative Commons license, and indicate if changes were made.
In cases where reviewers are anonymous, credit should be given to 'Anonymous Referee' and the source.
The images or other third party material in this Peer Review File are included in the article's Creative Commons license, unless indicated otherwise in a credit line to the material. If material is not included in the article's Creative Commons license and your intended use is not permitted by statutory regulation or exceeds the permitted use, you will need to obtain permission directly from the copyright holder.
To view a copy of this license, visit https://creativecommons.org/licenses/by/4.0/

REVIEWER COMMENTS

Reviewer #1 (Remarks to the Author):

This manuscript presents a new set of algorithms for local ancestry inference (LAI). The G-Nomix LAI tool is reported to achieve higher accuracy and greater speed than the widely used RFMix LAI program as well as the ELAI and Loter methods. The speed increase in particular is crucially important for the research community, as RFMix is painfully slow and does not scale to large, biobank-scale genomic datasets. The companion Gnofix tool provides for rapid and accurate phase correction. The paper is very well written, and the figures are outstanding; the algorithm is comprehensively documented, and the choices are well justified; benchmarking appears to have been comprehensive. Accordingly, I recommend that the manuscript be accepted for publication once my comments are addressed. Since I am a user of ancestry inference methods, rather than a developer, I will focus my comments on the programs' utility as opposed to the details of the algorithm. In addition to my comments on the manuscript, I provide discretionary suggestions for additional information that could make the GitHub repository even more useful, thereby (potentially) increasing community uptake of the method.

Major points

1. The Brownings recently developed the LAI program FLARE https://www.biorxiv.org/content/10.1101/2022.08.02.502540v1, which reportedly also provides high accuracy and speed for large datasets. It would be helpful to compare the performance of Gnomix to FLARE.

We now include a benchmark with FLARE showing that Gnomix outperforms in both accuracy and speed on whole genome data. We have also benchmarked against Orchestra published in this journal last year and also show that we outperform it by a large margin, Lerga-Jaso, J., Novković, B., Unnikrishnan, D., Bamunusinghe, V., Hatorangan, M.R., Manson, C., Pedersen, H., Osama, A., Terpolovsky, A., Bohn, S. and De Marino, A., 2025. Tracing human genetic histories and natural selection with precise local ancestry inference. Nature Communications, 16(1), p.4576. These findings are in accord with other benchmarking studies published in the last year.

2. Gnomix is benchmarked with highly divergent continental ancestry groups. It would be of great interest to know how the program performs on more closely related subcontinental ancestry groups. For example, could the method distinguish Northern European, Southern European, and Finnish haplotypes? This could be particularly useful given the motivation of local ancestry informed GWAS methods in light of the challenges that even fine-scale population structure pose to GWAS. Or is the method currently limited to continental-level ancestry deconvolution?

We focus on continental populations as these are the simplest to separate using Gnomix out-of-the-box without parameter tuning, but we included West Asian (a population group closely related to European) in order to demonstrate the ability of Gnomix to resolve even neighboring populations that are on a genetic cline. As can be seen from the figure "Effect of

population structure on learnt models," the smoother weighs more windows on either side of the target window in this case, meaning that separating regional ancestries from one another is possible, but requires wider context, meaning haplotypes should be longer, which is to say admixture must be relatively recent for such related groups.

3. Gnomix is benchmarked using simulated datasets, which is standard and entirely reasonable given the need for a ground-truth against which to compare. Nevertheless, I wonder if it would be possible to test on at least a few trios of admixed genomes using the pre-trained models available on their GitHub repository. For example, AMR trios from the 1000 Genomes Project.

This would definitely be interesting. We will consider including such analyses in future work.

4. Gnomix is benchmarked using a single (relatively small) chromosome, which again is quite reasonable given the comprehensiveness of the benchmarking performed. Nevertheless, one of the big selling points of the method is its speed and applicability to biobank-scale datasets. It would be very nice to see the performance results of applying this method to a large biobank scale data set of whole genome data sets (either sequences or genotypes) such as UK Biobank or All of Us.

As shown in other work, GNOMIX has been applied at biobank scale across all of All of Us and in the Mexican Biobank project, demonstrating scalability [1][2].

[1] Sohail, M., Palma-Martínez, M. J., Chong, A. Y., Quinto-Cortés, C. D., Barberena-Jonas, C., Medina-Muñoz, S. G., ... & Moreno-Estrada, A. (2023). Mexican Biobank advances population and medical genomics of diverse ancestries. *Nature*, 622(7984), 775-783.

[2] Bonet, D., Yang, J., Hastie, T., & Ioannidis, A. G. (2026). Biobank-Scale Polygenic Prediction in Admixed Populations Using Local Ancestry via the Group Lasso. *bioRxiv*, 2026-03.

4a. A corollary point is that it is difficult to make out the actual speed of their two preferred methods from Figure 4, which is very dense as many methods are evaluated. A simple visual representation of the time of running their two best performing algorithms programs on X thousands of genomes, compared to ELAI and Loter (and perhaps FLARE), would be very helpful.

We have added Table 1 to the main text, summarizing accuracy and inference time across all methods for the Latin American, 5-ancestry, and 7-ancestry datasets. This provides explicit numerical comparisons, making it easier to directly assess the performance and runtime of our two best-performing methods relative to competing approaches.

Minor points

5. How many genomes (chromosome 20s) were simulated for the benchmarking tables (Appendix D)? Is that what size refers to in Table 1?

For the benchmarking tables in Appendix D, we simulated 378 (5-ancestry), 406 (7-ancestry), and 182 (Latin American) admixed individuals, corresponding to three times the

number of testing founders, as described in the methods. The “Size” column in Table 1 instead refers to the total number of simulated individuals (training + testing). We have clarified this and explicitly reported the number of simulated test individuals in the main text.

6. What were the computational resources used for benchmarking, e.g. RAM and cores used?

All benchmarking experiments were conducted using 32 CPUs and 256 GB of RAM, as now clarified in the revised manuscript.

7. Why does the Latin American dataset contain so many more AFR founders, compared to EUR and NAT?

We aimed to create a realistic three-way admixture example by mimicking admixture proportions in the Latin American Caribbean, where populations with substantial proportions of three ancestries (AFR, EUR, and NAT) are common. African-like ancestry is much more prevalent on average in these populations, so we used more AFR founders (about 2x) than EUR and fewer still (½ as many) of NAT to reflect these Caribbean proportions. We included all available Spanish samples from 1000 Genomes and HGDP for the European component.

8. The manuscript mentions that larger windows may span ancestry switch points. Does the use of the smoother algorithm have the same potential resolution problem? In other words, how can one be sure that the smoother does not artificially concatenate same-ancestry haplotypes that actually come from different chromosomes? Is this one source of the error seen for higher generation time since admixture runs?

The smoother may in principle blur nearby switch points, but in general only the window containing the switchpoint itself is guaranteed to be blurred. The manuscript shows that errors are concentrated near true ancestry switch points and increase at higher generations since admixture, where switch points are more frequent and segments become shorter.

Discretionary GitHub suggestions

The GitHub is detailed and very useful, and the Jupyter notebook demo is a nice addition. Accordingly, these comments are purely discretionary for the authors’ consideration.

9. It would be very nice to know what kind of data QC should be done before running the program, e.g. should LD and/or MAF pruning be performed?

The most important preprocessing and QC steps are careful variant harmonization between reference and query data, retention of high-quality biallelic SNPs, and phasing before inference. We have added this in both the manuscript and the GitHub repository.

10. Similarly, data QC recommendations for model training would be helpful. Should LD pruned reference datasets be used? Should 10M SNPs or 100,000 SNPs be used for training?

In general, we would not recommend LD pruning of the reference panel, since Gnomix is designed particularly to harness the local haplotype structure and correlation patterns of

nearby SNPs. Denser SNP sets allow for smaller windows and greater resolution. We would favor using as many high-quality harmonized SNPs as practical. We have added this in both the manuscript and the GitHub repository.

11. Large datasets often run into too much memory consumption (e.g. Beagle for phasing). It would be nice to know how program parameters affect memory consumption. Memory consumption is mentioned for two parameters, but not for window_size, smooth size etc.

Larger window sizes increase the number of SNPs processed by each model and therefore increase memory usage. We have clarified this point in the manuscript.

12. For the window_size_cM parameter, it states “use larger windows if snp density is lower e.g. genotype data vs. sequence (default .5)”. This is helpful but specific parameter recommendations based on the type of data would be nice. For example, what parameters should be used for WGS data from All of Us versus genotype data from the UK Biobank?

As a practical starting point, we would recommend smaller windows for high-density WGS data such as All of Us (e.g. around 0.2 cM) and larger windows for array data such as UK Biobank (e.g. around 0.5 cM), with final tuning based on the SNP density of the dataset and validation performance.

13. In general, recommendations on what parameter values to use according to the number of SNPs & samples, and perhaps what computational resources should be enough for them, would be helpful. For example, for running Gnomix on the All of Us platform, should 4 cores and 32 GB RAM or 40 cores and 350GB RAM be requested. Research of this kind is increasingly cloud-based, with charges by the hour for every resource used. How does running Gnomix on 10,000 samples vs 100,000 samples impact the computational resource requirement?

In general, computational cost scales approximately linearly with the number of windows induced by the chosen window size. For moderate datasets (~10,000 samples), runs are feasible on modest resources (a few cores and tens of GB of RAM), whereas larger datasets (~100,000 samples) benefit from increased parallelization and higher memory. Gnomix is designed to scale efficiently with available CPUs due to its window-based parallelism, so users can trade off runtime against compute resources depending on their environment. We have added some guidance on resource requirements.

Reviewer #2 (Remarks to the Author):

Hilmarsson et al developed a new local ancestry inference method. The authors use simulated data to demonstrate that their new method is faster, and more accurate than the current state of the art local ancestry inference methods. The authors also present a new method to correct for phasing errors using local ancestry results, which has similar accuracy to RFMix but is significantly quicker. The authors also apply their method to a dataset of dogs, and demonstrated that admixture between European and pre-Columbian dogs occurred in a similar time frame to the admixture of Native American and European humans in Mexico. These improvements to local ancestry inference will be very beneficial due to the increasing size of diverse genetic datasets. Therefore, this method has the potential to be very impactful on the field of genomics and population genetics. I only have minor comments/suggestions for the authors to address.

1. Is there a figure that shows the results of the ADMIXTURE analysis mentioned in section 3.1? What cut-off was used to determine an individual as a single ancestry individual? Were any variant filters applied before running admixture, such as LD pruning or minor allele frequency filters?

We did not include a figure showing the ADMIXTURE results, but we have clarified the cut-off used to determine single-ancestry individuals; namely, those with ≥95% assignment probability to a single ancestry cluster. Prior to running ADMIXTURE, we applied liftOver to a common genome build, retained high-quality biallelic SNPs, removed second-degree and closer relatives, and performed phasing. We did not apply LD pruning, as Gnomix is designed to harness the local haplotype structure and correlation across nearby SNPs. These details have now been clarified in both the manuscript and the GitHub repository.

2. When running RFMix in figure 4D, did you change the parameter for time since admixture to match the simulated value? If you kept this parameter as the RFMix default value, how might that impact the accuracy of RFMix on your simulated data?

In our experiments, we used the default RFMix settings and did not tune the time-since-admixture parameter to match the simulated values. While providing the true value would likely improve performance, this parameter is typically unknown in real-world applications.

3. I think more details are required in the methods or results section for the general reader to understand how fitting an exponential curve to the distribution of local ancestry segment lengths results in being able to estimate the time of admixture.

We thank the reviewer for this suggestion and agree that additional clarification would be helpful. We have expanded the manuscript to better explain that, under a standard recombination model, local ancestry segment lengths follow an approximately exponential distribution [1], where the rate parameter is directly related to the time since admixture. By fitting an exponential curve to the observed segment length distribution, one can therefore estimate the admixture time.

[1] Gravel, S. (2012). Population genetics models of local ancestry. Genetics, 191(2), 607-619.

4. Page 8 lines 279-281. What figures/tables support this observation? Can you cite them here?

Robustness across generation time regimes is demonstrated in Figure 2d, while robustness to the population set and ancestry complexity is supported by Table 1 and Figure 2a–b, which show consistent performance across Latin American, 5-ancestry, and 7-ancestry cohorts. Additionally, the challenge of closely related populations such as West Asian and European is illustrated in Figure A13 and Figure 3, where confusion patterns and model behavior are analysed. We have now added explicit citations to these figures and tables in the revised manuscript.

5. What parameters were used when phasing with Beagle? Was a genetic map used and/or a reference panel?

Phasing was performed using Beagle 5.1 with default parameters. No reference panel was used, but all 1000 Genomes and HGDP samples were phased together, and a sex-averaged genetic map was applied. This is now in the methods.

6. How were the human genetic datasets transferred to GRCh37? Was a program like liftover and the UCSC genome browser chain files used?

Yes, liftover was used with the UCSC chain files. This is now in the methods.

7. Can you estimate how much of the Chihuahua genome is identified as being from Coyotes, similar to how you estimate the amount of arctic ancestry in Figure 8a?

The percentage of Chihuahua genome identified as being from Coyotes is 6.2%.

Reviewer #3 (Remarks to the Author):

Hilmarsson et al present a new method for local ancestry inference (LAI) which they describe as as “ flexible collection of new local ancestry algorithms”.
I agree with authors that the time is ripe for new LAI methods, especially ones that scale to the large size of current population genomics datasets. So it is good to have new methods being proposed that can handle large genetic datasets.
I see many positive aspects to the proposed method and manuscript. It claims higher accuracy and faster performance than some other methods, especially in many way (e.g. 7) admixtures. It can work on array or sequence data, it includes a phase correction step, and is open source and available. The manuscript is clearly written and contains a number of useful explanatory figures.
Towards the goal of developing scalable LAI methods suitable for biobank data sets, I find two main contributions from the current manuscript. First, the authors have developed a flexible framework for LAI using a broad array of (supervised and discriminative) classification methods. They do this by applying classifiers in genomic windows, with an additional smoothing step to better identify local ancestry tracts that span multiple windows. Second, the authors present a new kernel for a support vector machine (SVM), CovRSK. This kernel tries to address the problem of how much more weight should be given to matching contiguous genomic regions as the length of the match increases. Intuitively longer matches carry more information about ancestry, but how much more? CovRSK employs stochastic sampling to thin redundant features. CovRSK turns out to be most useful when the SNP data is not dense where it helps avoid overfitting.
I see how the flexibility offered by Gnomix can allow researchers to select an ideal tradeoff between speed and accuracy for their specific task and resources. However I worry a bit that this flexibility will more often be an overall net detriment to users, as external users may either not be sophisticated enough or willing to devote enough time to get it right.
The manuscript presents analyses of Gnomix applied to canid species. I’m not familiar with the canid literature, but I agree with the authors that ~90% accuracy across 12 ancestries is doing pretty well. It is notable how often the broad ancestry categories show up in both chromosomes of the Xoloitzcuintle dog in Figure 8b.

Major comments
The manuscript applies and tests a large number of different machine learning methods but the details of how each method is applied is not described. Many of the methods are sensitive to hyperparameters, such as learning rate , tree depth, objective function, etc, but these are not described in the manuscript.

We now report the key hyperparameters in Appendix I.

I’m a bit concerned with how the superpopulations are divided into test and training data sets. The superpopulations are made up of (sub)populations and the individuals within each of these pops will be more closely related than to others in the superpopulation. This can cause biases if the data are naively split within each superpopulation into test and training sets, and will increase the variance across splits. A number of these superpopulations are small with small (sub)populations which can exacerbate the issue. Perhaps one (sub)population could be left out each time, not sure. To me this is one of the chief difficulties when using real data to validate methods.

We agree that population structure can introduce bias if not handled carefully. To mitigate this, we split data at the founder level of the simulation and evaluate on independently simulated admixed individuals, reducing leakage between training and test sets.

In a related issue, I don't see any CI around the estimates of accuracy in tables 2-7, why not? My initial thought is the data split and model generating and LAI procedure could be repeated to generate a CI around the mean accuracy. CIs would then allow significant differences to be highlighted in the table rather than then larger of 97.79 vs 97.70 which I don't think are likely to be significantly different.

Repeating the full pipeline across multiple splits is computationally prohibitive. Instead, we report mean accuracy over large simulated test sets, which already reduces variance.

The authors do not compare their method to recent Li and Stephens HMM methods, e.g. MOSAIC in 2019 (https://doi.org/10.1534/genetics.119.302139) or FLARE in 2022 (https://doi.org/10.1101/2022.08.02.502540) and instead compare to ELAI from 2014. This is especially important as the MOSAIC paper showed it outperformed ELAI, and FLARE showed it outperformed MOSAIC with large reference sizes.

We now include a benchmark with FLARE showing that Gnomix outperforms. We have also benchmarked against Orchestra published in this journal last year and also show that we outperform it by a large margin, Lerga-Jaso, J., Novković, B., Unnikrishnan, D., Bamunusinghe, V., Hatorangan, M.R., Manson, C., Pedersen, H., Osama, A., Terpolovsky, A., Bohn, S. and De Marino, A., 2025. Tracing human genetic histories and natural selection with precise local ancestry inference. Nature Communications, 16(1), p.4576. These findings are in accord with other benchmarking studies published in the last year.

I found the main simulated admixture scenario presented in the main text somewhat unrealistic - e.g. a simultaneous 7-way admixture of equal proportions. This likely presents a challenging LAI problem, and is fine to include, but it doesn't really represent the majority of common LAI analyses and is the only result presented in the main text. The results from tables 2-7 should all be present in the main text, as a figure or table, maybe in a simplified form.

We have added Table 1 to the main text, summarizing accuracy and inference time across all methods for the Latin American, 5-ancestry, and 7-ancestry datasets. Full results remain in the Appendix D.

In the abstract the authors (correctly) note other LAI methods are burdened by "large next generation datasets" and "massive, whole genome biobanks". One of the main benefits of these large datasets are huge reference panels for supervised tasks such as LAI. However, despite the current manuscript describing "biobanks consisting of hundreds of thousands of individuals" as common, it does not attempt to show how Gnomix could be applied with reference panels approaching this size. The largest number of reference individuals seems to be 1290. In comparison, FLARE (https://doi.org/10.1101/2022.08.02.502540) considered reference panels up to 30K, and showed increasing accuracy with increasing ref sizes. Including larger reference panels would strengthen the claims in the manuscript that Gnomix is the method best suited to tackle "large next generation datasets".

As shown in other work, GNOMIX has been applied at biobank scale across all of All of Us and in the Mexican Biobank project, demonstrating scalability [1] [2].

[1] Sohail, M., Palma-Martínez, M. J., Chong, A. Y., Quinto-Cortés, C. D., Barberena-Jonas, C., Medina-Muñoz, S. G., ... & Moreno-Estrada, A. (2023). Mexican Biobank advances population and medical genomics of diverse ancestries. Nature, 622(7984), 775-783.

[2] Bonet, D., Yang, J., Hastie, T., & Ioannidis, A. G. (2026). Biobank-Scale Polygenic Prediction in Admixed Populations Using Local Ancestry via the Group Lasso. bioRxiv, 2026-03.

Minor comments

Depending on the dataset, there is a real potential for related individuals among the human data. Are steps taken to identify and remove related individuals? This could be a problem if related individuals are split across the test and training data sets.

KING was used to filter out second degree and closer related individuals from the reference data, as now clarified in the revised manuscript.

Versions of the programs compared to were not noted - e.g. was RFMIx v1 or V2 used?

We used RFMIx V2 and have added it to the manuscript.

How does the DEV data set seem to have more samples than are available for some populations? (WAS and NAT). Maybe I am misunderstanding table 1.

The dev dataset consists of simulated admixed individuals generated from random recombination amongst the available founders, rather than the founders themselves. As a result, the number of samples in the dev dataset can exceed the number of individuals in specific source populations such as WAS and NAT somewhat analogous to bootstrapping with replacement.

What does the size column in table 1 mean?

The “Size” column in Table 1 refers to the total number of simulated individuals (training + testing). We have clarified this and explicitly reported the number of simulated test individuals in the main text.

The authors state that Gnomix enables “portable models that are particularly useful when training data are not shareable due to privacy or other restrictions”. But it was not clear what privacy guarantees (if any) are able to be provided for models trained by Gnomix. Is it guaranteed that no training data could be inferred from examination or application of the model? Would biobanks accept this level of privacy for their data? If the authors wish to retain this statement, it should be supported.

We do not claim formal privacy guarantees such as differential privacy or immunity to model inversion. Rather, portability refers to the practical ability to share trained models without distributing individual-level genomic data, which is often sufficient in real-world settings. We have clarified this statement in the manuscript.

Sections 2.2.2 and 2.2.3 could be moved to the supplement. As is they take up some of the main focus of the paper (e.g. Figure 2) for an aspect of the paper not as directly related to local ancestry deconvolution.

We have moved the content previously presented in Sections 2.2.2 and 2.2.3 to the Appendix E.

I would prefer to see all of the evaluations applied to data which is not perfectly phased. This would involve an extra statistical rephasing step of the entire reference and target samples. Other recent LAI methods have done this (MOSAIC and FLARE) by considering diploid ancestry.

Our simulations make use of real sequences of founders that have been statistically phased using Beagle. As statistical phasing has been used it will include phasing errors. Furthermore, our discussion of the Gnofix technique explores how statistical phasing provides locally correct phasing but fails to capture long range phasing relationships, leading to distant variants being incorrectly phased. This is where Gnofix provides an improvement by leveraging ancestry information to correct phasing errors.

I would like to see ELAI and Loter included in the 3-way admixture analysis (called ”Latin American”), as these are the most realistic admixture scenarios.

Due to computational constraints, ELAI and Loter were only benchmarked with the 7-ancestry dataset. However, we have included benchmarks for Flare and Orchestra, two of the latest techniques, with Flare being considered by many the new gold standard method and Orchestra being published in this journal last year. We outperform both in accuracy and runtime on whole genome data.

Figure A1: The histogram could be normalized to allow easier comparison

We have updated Figure A1 to show normalized histograms (percentages), enabling clearer comparison between the training and validation distributions.

Figure A4:The uncalibrated curves are mirrored across the diagonal to visually illustrate the centrality of the calibrated ones. - this is not helpful. Maybe break this plot out by ancestry. And perhaps provide some additional commentary on if this is useful.

The mirroring was intentionally included as a visual aid to highlight deviations from perfect calibration by symmetrizing the uncalibrated curves around the diagonal.

Figure A7 and A8: This is a nice demonstration - but from my understanding the same ranking could be achieved with a simple score based on the fraction of matching sites (Hamming kernel), so this isn’t quite an example of where CovRSK outperforms.

These examples are intended primarily to illustrate differences between CovRSK and the standard string kernel, rather than to establish a case where CovRSK necessarily outperforms all simpler similarity measures such as Hamming similarity. We have revised the text accordingly to clarify the purpose of these figures.

Figure A11 and A12: should be combined into one figure and share a color scheme. Too hard to compare as is.

We agree and have combined Figures A11 and A12 into a single figure with a shared color scale to improve comparability. This makes it clear that, while both methods show similar error patterns, Gnomix exhibits fewer misclassifications overall.

Table 2 is this “whole genome” or just chromosome 20?. Does “whole genome” mean sequencing like data?

Table 2 refers to experiments performed on whole-genome sequencing–like data, but restricted to chromosome 20 rather than the entire genome.

** See Nature Portfolio's author and referees' website at www.nature.com/authors for information about policies, services and author benefits.

This email has been sent through the Springer Nature Tracking System NY-610A-NPG&MTS

Confidentiality Statement:

This e-mail is confidential and subject to copyright. Any unauthorised use or disclosure of its contents is prohibited. If you have received this email in error please notify our Manuscript Tracking System Helpdesk team at http://platformsupport.nature.com .

Details of the confidentiality and pre-publicity policy may be found here http://www.nature.com/authors/policies/confidentiality.html

Privacy Policy | Update Profile

DISCLAIMER: This e-mail is confidential and should not be used by anyone who is not the original intended recipient. If you have received this e-mail in error please inform the sender and delete it from your mailbox or any other storage mechanism. Springer Nature Limited does not accept liability for any statements made which are clearly the sender's own and not expressly made on behalf of Springer Nature Ltd or one of their agents.

Please note that Springer Nature Limited and their agents and affiliates do not accept any responsibility for viruses or malware that may be contained in this e-mail or its attachments and it is your responsibility to scan the e-mail and attachments (if any).

Springer Nature Ltd. Registered office: The Campus, 4 Crinan Street, London, N1 9XW. Registered Number: 00785998 England.

Reviews from manuscript "**High Resolution Ancestry Deconvolution for Next Generation Genomic Data**" from Nature Communications

Reviewer #1:

I am satisfied with the authors' responses to my comments and recommend that the revised manuscript be accepted for publication.

Author Remarks: **We thank the reviewer for their positive evaluation of our revised manuscript.**

Reviewer #2:

Hilmarsson et al sufficiently replied and addressed all of my comments. Their revised manuscript demonstrates the utility of Gnomix and how Gnomix improves on previously published local ancestry software. Gnomix will be a great local ancestry tool for the research community. I do not have additional comments for the authors to address.

The authors provided an extensive README and guide to help others install and use their code. This will greatly help their resource be used by members of the research community.

Author Remarks: **We appreciate the reviewer's support for the utility of Gnomix as a local ancestry tool for the research community.**

Reviewer #3:

The authors have thoroughly updated the manuscript and address all the major and minor concerns I had with the first version.

I have a few small comments.

1) In table 1, the timing unit is not specified, and training time is not included (I don't think). This leads to an inaccurate picture of the resources needed for an analysis, as GNOMIX preprocesses the data, while the other methods do not.

Author Remarks: **We thank the reviewer for pointing this out. We have added the timing unit to Table 1 to clarify the reported values. Training time is included separately in Supplementary Tables 4–9; we did not include it in Table 1 due to space limitations.**

2) In figure 2. The broken axes on panels a and b make it really hard to compare, I would suggest the break is removed.

Author Remarks: **We have removed the broken axes from panels 2a and 2b to improve readability and facilitate direct comparison across methods.**

3) FLARE seems to be the fastest method and has higher accuracy than RFMIX, so why was it not shown in Figure 2D?

Author Remarks: **We thank the reviewer for this helpful suggestion. We agree that FLARE is the fastest alternative to Gnomix and a valuable comparator to include in the generation-time analysis and have added it to Figure 2. For completeness, and so as not to privilege just RFMix and FLARE, we have also included other recent methods in this comparison. This expanded analysis shows that Gnomix maintains the highest accuracy across generation times, with the slowest decline in accuracy as ancestry segments become shorter. Since the figure became cluttered with all these methods, we have separated the array results into one panel and the whole genome into the other: Figure 2c presents the whole-genome results, and Figure 2d presents the array-genotype results. The previous classifier analysis in Figure 2c has been moved to the Supplementary Information.**

4) The open source repo is well maintained and available.

Author Remarks: **We appreciate the comment, and we commit to keeping the repository publicly available and properly maintained.**
